# Trends and risk factors of global incidence, mortality, and disability of genitourinary cancers from 1990 to 2019: Systematic analysis for the Global Burden of Disease Study 2019

**DOI:** 10.3389/fpubh.2023.1119374

**Published:** 2023-02-22

**Authors:** Yi-Qun Tian, Jin-Cui Yang, Jun-Jie Hu, Rong Ding, Da-Wei Ye, Ji-Wen Shang

**Affiliations:** ^1^Department of Urology, Tongji Hospital, Tongji Medical College, Huazhong University of Science and Technology, Wuhan, China; ^2^Cancer Center, Tongji Hospital, Tongji Medical College, Huazhong University of Science and Technology, Wuhan, China; ^3^Department of Geriatrics, Tongji Hospital, Tongji Medical College, Huazhong University of Science and Technology, Wuhan, China; ^4^Department of Ambulatory Surgery, Shanxi Bethune Hospital, Shanxi Academy of Medical Science, Tongji Shanxi Hospital, Third Hospital of Shanxi Medical University, Taiyuan, China

**Keywords:** genitourinary cancers, incidence, disability-adjusted life years (DALYs), mortality, global burden of disease (GBD)

## Abstract

**Background:**

The incidence of kidney, bladder, and prostate cancer ranked ninth, sixth, and third in male cancers respectively, meanwhile, the incidence of testicular cancer also increased gradually in the past 30 years.

**Objective:**

To study and present estimates of the incidence, mortality, and disability of kidney, bladder, prostate, and testicular cancer by location and age from 1990 to 2019 and reveal the mortality risk factors of them.

**Materials:**

The Global Burden of Diseases Study 2019 was used to obtain data for this research. The prediction of cancer mortality and incidence was based on mortality-to-incidence ratios (MIRs). The MIR data was processed by logistic regression and adjusted by Gaussian process regression. The association between the socio-demographic index and the incidence or disease burden was determined by Spearman's rank order correlation.

**Results:**

Globally in 2019, there were 371,700 kidney cancer cases with an age-standardized incidence rate (ASIR) of 4.6 per 100,000, 524,300 bladder cancer cases, with an ASIR of 6.5 per 100,000, 1,410,500 prostate cancer cases with an ASIR of 4.6 per 100,000 and 109,300 testicular cancer incident cases with an ASIR of 1.4 per 100,000, the ASIR of these four cancers increased by 29.1, 4, 22, and 45.5% respectively. The incidence rate of the four cancers and the burden of kidney cancer were positively correlated with the socio-demographic index (SDI), regions with a higher SDI faced more of a burden attributable to these four cancers. High body-mass index has surpassed smoking to be the leading risk factor in the past thirty years for kidney cancer mortality. Smoking remained the leading risk factor for cancer-related mortality for bladder cancer and prostate cancer and the only risk factor for prostate cancer. However, the contribution of high fasting plasma glucose to bladder cancer mortality has been increasing.

**Conclusion:**

The incidence of bladder, kidney, prostate, and testicular cancer is ever-increasing. High-income regions face a greater burden attributable to the four cancers. In addition to smoking, metabolic risk factors may need more attention.

## 1. Introduction

Cancer poses a major public health problem and may surpass cardiovascular disease to be the leading cause of premature death caused by non-communicable diseases in most countries this century (1). It is estimated that 19.3 million new cancer cases and almost 10.0 million cancer deaths occurred in 2020 (2). With population growth and aging, the incidence and mortality of kidney, bladder, prostate, and testicular cancer are increasing rapidly (3). According to the latest data from the International Agency for Research on Cancer, the incidence of kidney, bladder, and prostate cancer ranked ninth, sixth, and third, respectively, among males (2). Prostate cancer has surpassed lung cancer as the most commonly diagnosed cancer among males in 112 countries, with an estimated 1.4 million (7.3%) new cases in 2020 (2). Revealing the spatial, temporal prevalence and disease burden, as well as the major risk factors for these cancers by age, sex, and economic conditions will provide a better understanding of the epidemiology, thus guiding policy-making and medical decisions in the prevention and management of these cancers.

Previous studies of genitourinary cancer have described the disease burden confined to a single kind of cancer or failed to reveal the differentiation of incidence, mortality, and risk factors between males and females for kidney and bladder cancer (4–7). In this study, we describe the up-to-date trends of national, regional, and global level mortality and disability-adjusted life years (DALYs) from 1990 to 2019 by age, sex, and socio-demographic index for kidney, bladder, prostate, and testicular cancer, according to the Global Burden of Diseases Study 2019 (GBD, 2019). We aim to reveal the changes over time in the incidence and burden of the four genitourinary cancers to provide references for health policy-making and medical management decisions.

## 2. Materials and methods

### 2.1. Data source diseases definition

Input data from GBD 2019 was utilized to generate the estimation of genitourinary cancers incidence and burden. In the study, the data source for genitourinary cancers included hospital records, emergency department records, insurance claims, surveys, and vital registration systems globally. The methodology of data inputting, mortality estimation, and modeling for GBD 2019 has been comprehensively reviewed in previously published articles (8, 9). The definition of genitourinary cancers counts on the International Statistical Classification of Diseases and Related Health Problems 10th Revision (ICD-10). In the GBD study, genitourinary cancers in the study have four dimensions: kidney cancer, bladder cancer, prostate cancer, and testicular cancer, their ICD-10 codes are stated as follows: C64-C65.9, D30.0-D30.1, and D41.0-D41.1 for kidney cancer; C67-C67.9, D09.0, D30.3, D41.4-D41.8, and D49.4 for bladder cancer; C61-C61.9, D07.5, D29.1, and D40.0 for prostate cancer; C62-C62.9, D29.2-D29.8, and D40.1-D40.8 for testicular cancer (Table 1S).

### 2.2. Socio-demographic index

The burden of genitourinary cancers was evaluated against country-level development restrained with the SDI (10, 11) which is a composite indicator of three indicators: lag-distributed income per capita, average educational attainment for people aged 15 years and older, and the total fertility rate (in people aged <25 years). The 204 countries and territories were classified into five groups: low SDI (<0·45), low-middle SDI (≥0·45 and <0·61), middle SDI (≥0·61 and <0·69), high-middle SDI (≥0·69 and <0·80), and high SDI (≥0·80) on the basis of the SDI values.

### 2.3. Risk factors

GBD risk factors are estimated based on a comparative risk assessment framework which includes six steps. First, the identification of risk-outcome pairs: only those risk-outcomes, which have convincing or plausible evidence according to the World Cancer Research Fund criteria (12), will be involved in GBD risk factor estimation. Second, relative risk (RR) as a function of exposure for each risk-outcome pair is estimated. Third, exposure for each risk factor is distributed by age, sex, location, and year. Fourth, the theoretical minimum risk exposure level (TMREL) is demonstrated. Fifth, the population attributable fraction (PAF) and attributable burden are measured. The PAF is modeled by the RR for each risk-outcome pair, exposure levels, and TMRE L (9). The PAF of a particular risk factor is compounded by genitourinary cancer mortality to engender the mortality attributable to that risk factor. Finally, the PAF and attributable burden for the combination of risk factors are estimated. The methodology of these steps has been comprehensively reviewed in previously published articles (9). Four [high body-mass index (BMI) for kidney cancer, occupational exposure to trichloroethylene for kidney cancer, high fasting plasma glucose for bladder cancer, and smoking for kidney, bladder and prostate cancer] of the 87 risk factors included in this GBD iteration have a non-zero contribution to the mortality of genitourinary cancers deaths. The percentage contribution of these four risk factors to genitourinary cancers is assessed.

### 2.4. Statistical analysis

In this study, the age-standardized incidence rate (ASIR), mortality rate (ASMR), and DALYs rate (ASDR), as well as the 95% uncertain intervals (95% UI) are presented to show the epidemiology and burden of genitourinary cancers. We perform the 95% UI in the estimation process with the Bayesian-based model DisMod-MR 2.1. The estimated annual percentage change (EAPC) was calculated *via* age-standardized rates (ASR) in each year from 1990 to 2019 to indicate the trends of ASIR, ASMRand ASYR with time. Supported by the assumption that the ASRs are linearly correlated with time, the estimation of EAPC was represented by the model y = a + bx + e. In this model, y represents log10 (ASR), while x indicates calendar year, and b stands for regression coefficient. The EAPC was calculated as EAPC = 100^*^ (10∧b-1) based on this model. If the EAPC value and its lower bound of 95% CI are above zero, ASR is considered to be in an upward trend, and vice versa. Spearman's rank order correlation was used to determine the correlation between the SDI and ASIR, ASMR and ASDR. Statistical significance was defined as the *p*-value < 0.05. R software (version 3.6.3) was used to perform all statistical analyses.

## 3. Results

### 3.1. The incidence and temporal trends of four genitourinary cancers

**Figure 1:**
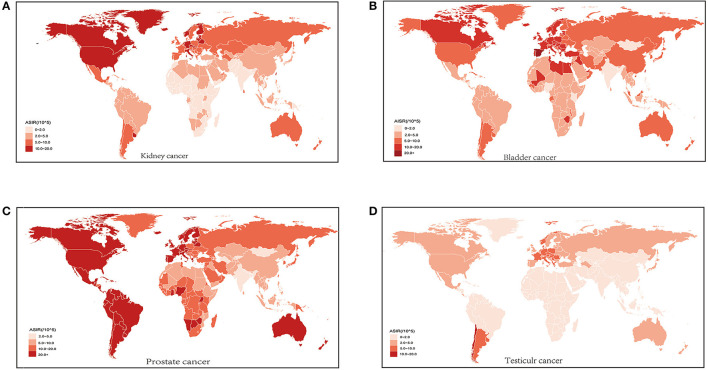
Age standardized incidence rate (ASIR) of kidney cancer **(A)** bladder cancer **(B)** prostate cancer **(C)** and testicular cancer **(D)** for both sexes in 204 countries and territories in 2019.

Globally in 2019, there were 371,700 (131,700 females and 240,000 males) (95%UI, 344,600 to 402,300) kidney cancer cases with an ASIR of 4.6 per 100,000, 524,300 (116,500 females and 407,008 males) (95% UI, 476,000 to 569,400) bladder cancer cases, with an ASIR of 6.5 per 100,000, 1,410,500 (95% UI, 1,227,900 to 1,825,800) prostate cancer cases with an ASIR of 4.6 per 100,000 and 109,300 (95% UI, 93,300 to 129,400) testicular cancer incident cases with an ASIR of 1.4 per 100,000 (1). Compared to 1990, the ASIR increased for all four cancers in 2019, with the largest increase seen for testicular cancer (45.5%; 95% UI, 27.4 to 71.3%). At the regional level, Western European had the most incident cases and the largest ASIR for bladder cancer, despite a decreased incident rate. High-income North America had the most incidence cases in 2019 for both kidney and prostate cancer. The most incident cases of testicular cancer occurred in Western European, while the largest ASIR was in Southern Latin America in 2019. However, the largest increase in new cases for the four cancers was observed in East Asia (Table 1). Geographically, countries with high incidence rates of the four cancers were mainly located in high-income regions, such as North America, Europe, and Australia (Figure 1; Supplementary Figure 2S), while countries in Africa, the Middle East, and East Asia had relatively large increases of incidence rates, compared to those in developed regions (Supplementary Figure 1S).

**Table 1:** Incident number, age standardized incident rate in 2019 and the percentage change of incident rate for bladder, kidney, prostate and testicular cancer between 1990 and 2019.

**Region**	**Bladder cancer**			**Kidney cancer**			**Prostate cancer**			**Testicular cancer**		
	**Incident number (×1000) in 2019**	**Age standardized incident rate per 100,000 in 2019**	**Change of age standardized incident rate between 1990 and 2019**	**Incident number (×1000) in 2019**	**Age standardized incident rate per 100,000**	**Change of age standardized incident rate between 1990 and 2019**	**Incident number (×1000) in 2019**	**Age standardized incident rate per 100,000**	**Change of age standardized incident rate between 1990 and 2019**	**Incident number (×1000) in 2019**	**Age standardized incident rate per 100,000**	**Change of age standardized incident rate between 1990 and 2019**
Global	524.3 (476 to 569.4)	6.5 (5.9 to 7.1)	4% (-4.3% to 13.5%)	371.7 (344.6 to 402.3)	4.6 (4.2 to 4.9)	29.1% (18.7% to 40.7%)	1410.5 (1227.9 to 1825.8)	17.4 (15.1 to 22.5)	22% (9.8% to 45.9%)	109.3 (93.3 to 129.4)	1.4 (1.2 to 1.7)	45.5%(27.4% to 71.3%)
East Asia	105.4 (88.4 to 124.6)	5.3 (4.4 to 6.2)	55.6% (26.1% to 95.8%)	64.3 (54.1 to 75.6)	3.3 (2.8 to 3.9)	177.2% (126.8% to 244%)	162 (126.3 to 213.7)	7.9 (6.2 to 10.4)	111.6% (63.7% to 177.4%)	17.8 (14.4 to 22.1)	1.2 (1 to 1.5)	321.7% (182.4% to 500%)
Southeast Asia	16.3 (14.2 to 19.1)	2.8 (2.5 to 3.3)	26.1% (7.1% to 50.5%)	16.4 (13.4 to 20.8)	2.6 (2.1 to 3.3)	78.6% (44.1% to 119.5%)	44.5 (34.3 to 52.6)	8.3 (6.4 to 9.8)	61.9% (35.6% to 94.1%)	4.1 (3.1 to 5.5)	0.6 (0.5 to 0.8)	142.2%(78.8% to 231%)
Oceania	0.2 (0.1 to 0.2)	2.5 (2 to 3.1)	32% (8.8% to 61.3%)	0.1 (0.1 to 0.1)	1.1 (0.9 to 1.4)	14.7% (-6.4% to 39.9%)	0.7 (0.5 to 0.8)	12.9 (9.6 to 16.2)	28.2% (2.5% to 61%)	0.05 (0.03 to 0.07)	0.5 (0.4 to 0.7)	−3.2%(-30.3% to 34.2%)
Central Asia	3.3 (2.9 to 3.6)	4.6 (4.1 to 5)	30.5% (13.8% to 56.5%)	3.9 (3.5 to 4.3)	4.7 (4.3 to 5.2)	60.9% (34.9% to 92.6%)	4.8 (3.8 to 5.7)	7.3 (5.8 to 8.8)	62.3% (43.4% to 84.9%)	1.2 (0.8 to 1.9)	1.3 (0.9 to 2)	74.6% (29.8% to 141%)
Central Europe	27.1 (23.6 to 30.7)	12.6 (11 to 14.3)	50.3% (30.3% to 70.7%)	17.7 (15.5 to 20.1)	8.9 (7.8 to 10.1)	114.5% (88.5% to 141.5%)	43.8 (32.1 to 51.7)	19.5 (14.2 to 23)	92.3% (49.3% to 127.5%)	7.2 (5.2 to 9.3)	7 (4.6 to 9.7)	115.4% (50.9% to 194.7%)
Eastern Europe	24.4 (21.6 to 27.4)	7.1 (6.3 to 7.9)	29% (14.4% to 44.8%)	32.5 (29.2 to 36.3)	10 (9 to 11.2)	51.4% (33.4% to 71.8%)	64.7 (45.8 to 79.3)	18.2 (12.9 to 22.3)	137.6% (72.1% to 188.4%)	5.7 (4.1 to 7.7)	2.8 (1.8 to 4.1)	110.5% (70.7% to 189.7%)
High-income Asia Pacific	35.4 (29.6 to 41.2)	7.5 (6.4 to 8.7)	5.3% (-8.2% to 20.8%)	17.7 (15.1 to 20.2)	4.5 (3.9 to 5.1)	48.5% (30% to 68.9%)	68 (52.4 to 91.3)	14 (10.9 to 18.9)	84.2% (49.4% to 124.3%)	3.2 (2.1 to 4.3)	2.1 (1.3 to 3.1)	3.6% (-38.7% to 83.8%)
Australasia	4.4 (3.6 to 5.5)	8.7 (7 to 10.9)	−16.9% (-32.5% to 3.2%)	4.1 (3.3 to 5.1)	9 (7.2 to 11.2)	20.8% (-3% to 51.5%)	25.7 (19.1 to 38)	51 (37.8 to 75.3)	41.6% (7.6% to 96.9%)	1.2 (0.7 to 2.0)	4.7 (2.5 to 8.1)	38.6% (-18.8% to 122.4%)
Western Europe	138.2 (119.2 to 159.3)	14.9 (12.8 to 17.3)	−0.9% (-14.5% to 14.5%)	75.6 (66.6 to 85.7)	9.3 (8.2 to 10.6)	28.1% (13.1% to 45.6%)	325.5 (267.1 to 469.9)	35.8 (29.3 to 51.5)	53.9% (29.1% to 104.8%)	20.5 (14.7 to 27.0)	5.4 (3.5 to 7.7)	25.4% (2.8% to 66.8%)
Southern Latin America	6 (4.7 to 7.5)	7.1 (5.6 to 8.9)	−1.1% (−21.8% to 24.3%)	7.3 (5.6 to 9.3)	9.1 (6.9 to 11.6)	11.8% (−15.3% to 43.4%)	19.5 (14.6 to 27.2)	22.9 (17.2 to 31.9)	54.2% (21.7% to 94%)	4.9 (2.9 to 8.2)	7.5 (4.2 to 13.3)	152.5% (53.1% to 314.1%)
High-income North America	57 (49.4 to 65.6)	9 (7.8 to 10.4)	3.3% (−10.5% to 18.6%)	68.2 (58.8 to 78.4)	11.7 (10.1 to 13.5)	10.9% (−4.6% to 27.9%)	331.9 (262.4 to 494.6)	52.1 (41.2 to 77.5)	−2.2% (−24.5% to 45.7%)	14.1 (10.1 to 17.8)	4.2 (2.9 to 5.7)	10.8% (−11.4% to 45.4%)
Caribbean	2.8 (2.4 to 3.3)	5.5 (4.7 to 6.4)	20.7% (2.5% to 41%)	1.9 (1.5 to 2.2)	3.7 (3.1 to 4.4)	3% (−13.5% to 21.1%)	22.8 (17.7 to 28.1)	44.1 (34.3 to 54.4)	54.4% (24.7% to 83.4%)	0.3 (0.2 to 0.4)	0.6 (0.3 to 0.9)	245.2% (31.9% to 446.6%)
Andean Latin America	1.4 (1.1 to 1.7)	2.5 (2.1 to 3.1)	21% (−3.4% to 50.8%)	2 (1.6 to 2.5)	3.5 (2.8 to 4.3)	46.8% (14.3% to 86.7%)	12.1 (9.2 to 15.6)	22.5 (17.1 to 29)	75.5% (35.7% to 125.6%)	1.0 (0.7 to 1.5)	1.6 (1 to 2.3)	120.1% (−20% to 352.7%)
Central Latin America	6.2 (5.3 to 7.3)	2.7 (2.3 to 3.1)	16.3% (0.2% to 35%)	10.4 (8.9 to 12.1)	4.3 (3.7 to 5)	63.4% (40% to 89.4%)	65.1 (51.3 to 86.1)	28.4 (22.5 to 37.5)	79.6% (50.5% to 114.9%)	5.140 (3.0 to 7.239)	2.1 (1.2 to 3)	205.1% (89.8% to 290.6%)
Tropical Latin America	9.7 (9 to 10.4)	4.1 (3.8 to 4.4)	7.3% (−0.3% to 14.6%)	8.4 (7.9 to 8.9)	3.5 (3.3 to 3.7)	58.1% (47.6% to 68.4%)	56.9 (49.6 to 84.1)	24 (20.9 to 35.5)	46.3% (36.2% to 57.4%)	2.6 (1.7 to 3.5)	1.2 (0.7 to 1.7)	136.4% (77.1% to206.2%)
North Africa and Middle East	41.3 (34.7 to 50)	9.6 (8.1 to 11.4)	52.5% (21.3% to 107.1%)	15.7 (13.6 to 18)	3.2 (2.8 to 3.6)	98% (55.5% to 157.4%)	47.5 (37 to 55.8)	12 (9.3 to 14.1)	83.2% (55.7% to 123.8%)	8.5 (6.1 to 11.6)	1.4 (1 to 1.8)	244% (106.8% to 440%)
South Asia	31.6 (27.9 to 35.7)	2.4 (2.1 to 2.7)	9.4% (−7.7% to 33.2%)	16.8 (14.7 to 19.4)	1.1 (1 to 1.3)	81.4% (42.8% to 142.7%)	54 (44.2 to 69.7)	4.4 (3.6 to 5.7)	8.5% (-10.7% to 39.6%)	9.3 (7.4 to 11.8)	0.5 (0.4 to 0.6)	78.3% (40.3% to 133.1%)
Central Sub-Saharan Africa	1.7 (1.1 to 2.6)	3.8 (2.4 to 5.6)	−16.7% (−36.3% to 13.9%)	0.8 (0.6 to 1)	1.1 (0.8 to 1.6)	23% (-12.2% to 65.8%)	4.5 (3.2 to 5.8)	11.4 (7.9 to 14.6)	0.9% (-18.1% to 22.8%)	0.3 (0.2 to 0.5)	0.3 (0.2 to 0.4)	21.5% (−36.3% to 86.8%)
Eastern Sub-Saharan Africa	4.6 (4 to 5.4)	3.2 (2.7 to 3.7)	−1.6% (−24.9% to 21.7%)	3.3 (2.7 to 4.1)	1.6 (1.3 to 2)	54.5% (20.1% to 97.8%)	16.3 (12.9 to 20.2)	12.1 (9.6 to 14.8)	21.6% (6.5% to 38.3%)	1.1 (0.7 to 1.7)	0.2 (0.2 to 0.3)	48% (−20% to 123.7%)
Southern Sub-Saharan Africa	2.2 (2 to 2.4)	4 (3.6 to 4.5)	1.5% (−13.4% to 20%)	1.1 (1 to 1.2)	1.9 (1.7 to 2)	40.2% (20.2% to 58.2%)	10.1 (8.1 to 11.9)	20 (15.9 to 23.2)	30.6% (8.2% to 63.3%)	0.4 (0.3 to 0.5)	0.5 (0.4 to 0.6)	24.6% (−1.9% to 55.7%)
Western Sub-Saharan Africa	5.1 (3.5 to 6.1)	2.9 (2.1 to 3.5)	−3.7% (−39.3% to 24.9%)	3.6 (2.7 to 4.7)	1.2 (1 to 1.5)	45.2% (13.5% to 87%)	30.2 (16.8 to 40.6)	20.6 (11.5 to 27.4)	50.4% (16.7% to 92.7%)	0.8 (0.3 to 2.6)	0.1 (0.1 to 0.4)	−2.2% (−33% to 64.6%)

### 3.2. The estimates of the disease burden attributable to four genitourinary cancers

Kidney, bladder, prostate, and testicular cancer were associated with 166,440; 228,730; 486,840; and 10,820 deaths, respectively (Supplementary Table 2S) worldwide in 2019. From 1990 to 2019, the ASMR has decreased for bladder cancer (−15.71%; 95% UI, −21.04 to −8.59%), prostate cancer (−9.46%; 95% UI, −15.19 to −0.05%), and testicular cancer (−7.53%;95% UI, −15.88 to 1.35%) but has increased by 11.61% (95% UI, 4.64 to 19.98%) for kidney cancer. This increase mainly came from males (19.35%; 95% UI, 10.46 to 30.02%). At the regional level, East Asia had the largest increase in ASMR (85.13%; 95% UI, 47.58 to 131.41%) between 1990 and 2019 for kidney cancer, followed by Central Europe (83.17%; 95% UI, 60.8 to 105.76%). Australia had the largest decrease in ASMR of bladder cancer (−24.56%; 95% UI, −31.37 to −17.12%). High-income North America had the largest decrease for prostate cancer (−24.92%; 95% UI, −32.59 to 0.51%) and High-income Asia-Pacific had the largest decrease for testicular cancer (−41.12%; 95% UI, −46.8 to −33.8%) (Supplementary Figure 4S). At the country level, Uruguay, Greenland, and Czechia were the three countries with the largest ASMR and ASDR in 2019 attributable to kidney cancer (Supplementary Figure 3S, Supplementary Table 3S). Mali led other countries in ASMR of bladder cancer (10.06 per 100,000; 95% UI, 4.35 ~ 13.53 per 100,000) (Supplementary Figure 3S), Egypt was the leading country in bladder cancer attributable ASDR (201.75 per 100,000; 95% UI, 132.14 ~ 294.39 per 100,000), meanwhile, Tonga had the largest ASDR (190.62 per 100,100;91% UI, 129.3~278.5 per 100,000) and ASMR (6.89 per 100,000; 95% UI, 4.9~5.01 per 100,000) of testicular cancer. Countries with relatively high ASMR and ASDR associated with prostate cancer were mainly seen in Africa (Supplementary Figures 3S, 5S). The percentage change of ASMR between 1990 and 2019 showed the same geographical pattern as that of ASDR for all four cancers (Supplementary Figures 4S, 6S).

### 3.3. The association between incidences, the burden of the four genitourinary cancers, and SDI

**Figure 2:**
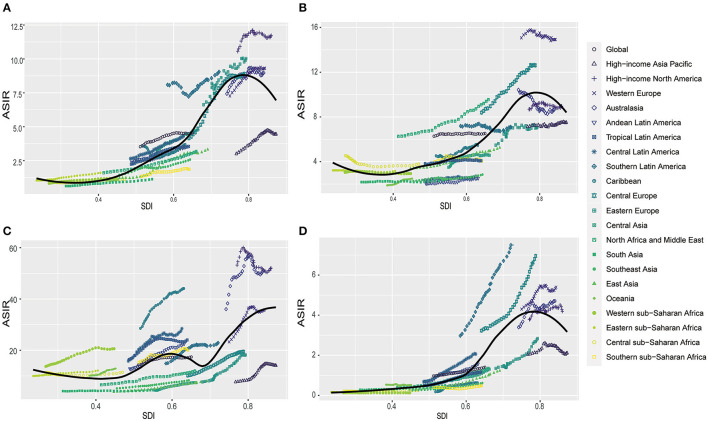
The correlation between global age standardized incidence rate (ASIR) and socio-demographic index (SDI) for kidney cancer **(A)** bladder cancer **(B)** prostate cancer **(C)** and testicular cancer **(D)** for both sexes in different regions.

**Figure 3:**
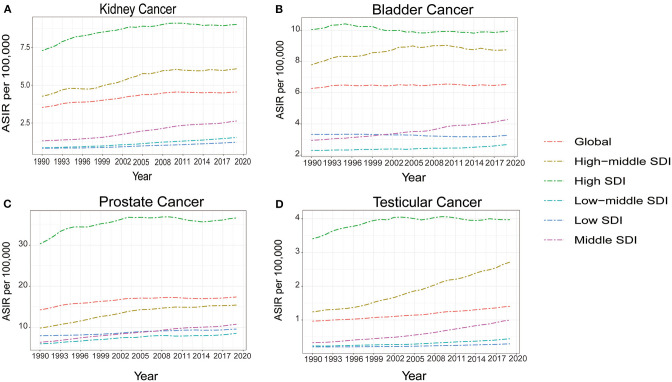
The temporal trends of age standardized incidence rate (ASIR) for kidney cancer **(A)** bladder cancer **(B)** prostate cancer **(C)** and testicular cancer **(D)** from 1990 to 2019, for both sexes by socio-demographic index (SDI).

From 1990 to 2019, the ASIR in different SDI regions gradually increased for kidney, bladder, prostate, and testicular cancer. During this period, high SDI regions had the largest ASIR each year compared to other regions. Spearman's rho correlation tests revealed a significantly positive association between ASIR and SDI for kidney cancer (rho, 0.8675; *p* < 0.001), bladder cancer (rho, 0.6928; *p* < 0.001), prostate cancer (rho, 0.551; *p* < 0.001), and testicular cancer (rho, 0.215; *p* < 0.001) (Figures 2, 3). For the disease burden, only in kidney cancer were the ASMR and ASDR positively correlated to SDI (Supplementary Figure 7S). In the past 30 years, the ASMR and ASDR of kidney cancer remained stable or slightly increased in different SDI regions, and gradual decreases were seen in most SDI regions for bladder and prostate cancer. As for testicular cancer, the ASMR and ASDR decreased gradually in the high and high-middle SDI regions but increased in the middle, low-middle, and low SDI regions (Supplementary Figure 8S).

### 3.4. ASMR and ASDR for both sexes and different ages.

**Figure 4:**
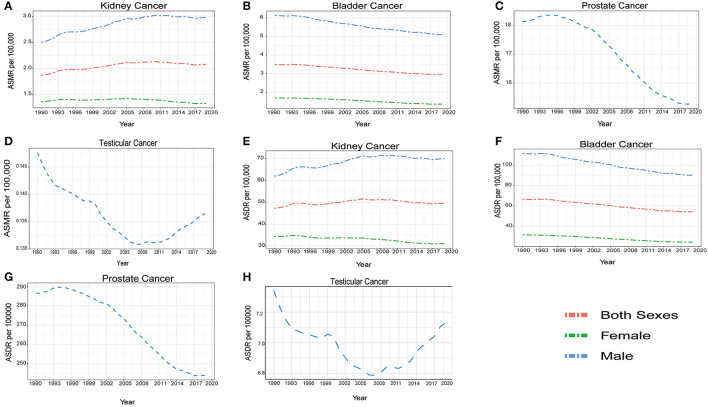
The temporal trends of global age standardized mortality rate (ASMR) and age standardized disability adjusted life year rate (ASDR) by sexes for kidney cancer **(A, E)** bladder cancer **(B, F)** prostate cancer **(C, G)** and testicular cancer **(D, H)** from 1990 to 2019.

**Figure 5:**
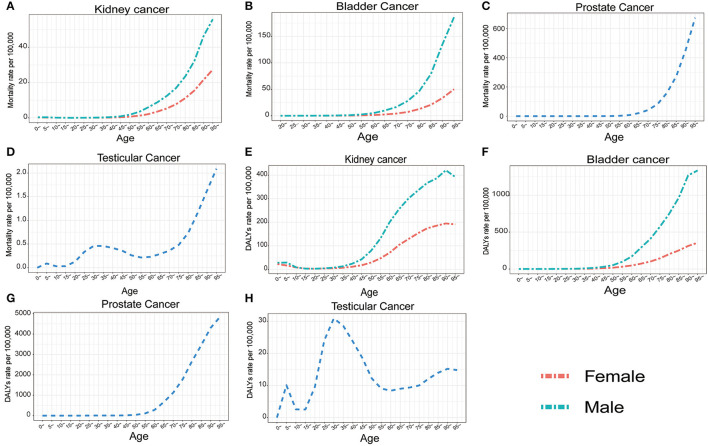
The change of global mortality rate and DALY rate per 100,000 people with age for kidney cancer **(A, E)** bladder cancer **(B, F)** prostate cancer **(C, G)** and testicular cancer **(D, H)** for both sexes in 2019.

From 1990 to 2019, the ASMR and ASDR for bladder cancer were higher in males, and the trends decreased for both sexes in this period (Figure 4). A downward trend was also observed in males for prostate cancer. However, for kidney cancer, as the ASMR remained relatively stable and the ASDR slightly decreased in females, they both increased in males during this period. Meanwhile, an obvious downward trend in the ASMR and ASDR was observed in testicular cancer from 1990 to 2008. However, both ASMR and ASDR increased gradually over the next 11 years (Figure 4). For different ages, more deaths and DALYs were seen in males for both bladder and kidney cancer (Supplementary Figure 9S). The mortality rates and DALYs increased with age for the two cancers, accompanied by large differences between the two sexes (Figure 5). Prostate cancer mainly affected people older than 40 years of age, and the number of deaths and DALYs increased with age until 75 to 85 years of age, and then decreased (Supplementary Figure 9S). However, the death rates and DALYs always increased from the age of 40 years (Figure 5). As for testicular cancer, the number of deaths mainly increased in men aged 15 to 30 years old and older than 70 years old, and the number of DALYs increased in children aged 0 to 5 years old and men of 15 to 30 years old. However. they mainly decreased from the age of 40 years old (Figure 5).

### 3.5. Risk factors for ASMR of the kidney, bladder, and prostate cancers

**Figure 6:**
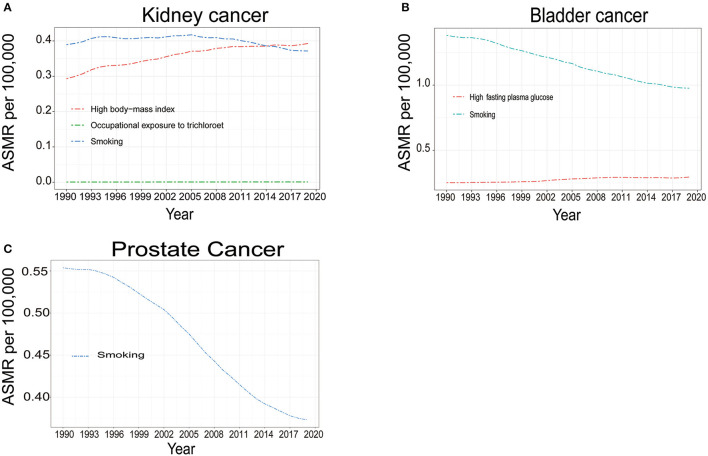
The temporal trends of global age standardized mortality rate (ASMR) attributable to risk factors of kidney cancer **(A)** bladder cancer **(B)** and prostate cancer **(C)** for both sexes from 1990 to 2019.

Figure 6; Supplementary Figure 11S show the risk factors for the ASMR of bladder, kidney, and prostate cancer in 2019. Smoking was a risk factor for mortality of all three cancers and was the leading risk factor for bladder cancer, and the only risk factor for prostate cancer. High fasting plasma glucose was another risk factor for bladder cancer and the trend of ASMR and ASDR caused by high fasting plasma glucose increased slightly in the past 15 years. For kidney cancer in both sexes, a high body-mass index had surpassed smoking to be the leading risk factor for ASMR and ASDR worldwide, as well as in all SDI regions, and remained the most important risk factor for ASMR and ASDR of kidney cancer in woman worldwide. However, smoking was still the top risk factor for ASMR and ASDR in male kidney cancer all over the world since 1990. Occupational exposure to trichloroethylene also accounted for a small portion of deaths in all SDI regions for kidney cancer (Figure 6; Supplementary Figures 10S, 11S). Globally, smoking-related deaths gradually decreased for the three cancers, but other risk factors related to death and DALYs increased for bladder and kidney cancer, indicating the important role of metabolic health in the management of bladder and kidney cancer.

## 4. Discussion

In this study, we found an increased age-standardized incidence rate for kidney, prostate, and testicular cancer in 2019, compared to 1990, indicating an increased incidence of these three cancers. In addition, the ASMR and ASDR increased for kidney cancer but decreased for bladder, prostate, and testicular cancer. As kidney, bladder, and prostate cancer are more likely to affect older people, the process of aging and the increased life expectancy might partially explain the growth in the incidences and the burden, especially in developed countries where population aging is more significant. In developing countries, the increase in the population also contributes to the increased cases of the four cancers. The total number of incidences of testicular cancer increased over the study period, probably as a result of population growth and the change in environments.

Of the four cancers evaluated, prostate cancer had more incident cases and a higher ASIR. From 1990 to 2019, the ASIR of prostate cancer in high SDI regions each year was greatly higher than that in the other SDI regions, which could be a result of the broadened prostate-specific antigen (PSA) screening for prostate cancer in developed countries that started in the 1990s (13). However, the role of PSA screening in the detection and treatment of prostate cancer is still controversial (14). A comprehensive meta-analysis suggested that PSA screening does not improve overall mortality, so the benefit of PSA screening and the risk of overdiagnosis and overtreatment should be balanced. Despite the increased incident cases and ASIR, the age-standardized mortality rate and DALYs both decreased for prostate cancer, as well as bladder and testicular cancer. These results revealed the advances in the management of the three cancers above, including advanced surgical strategies, novel chemotherapy agents, immune checkpoint inhibitors, and more effective hormone-based treatments (15, 16). Unlike bladder, prostate, and testicular cancer, the mortality and DALYs burden increased in 2019, compared to 1990, for kidney cancer. Kidney cancer also caused considerable disability for younger patients in childhood compared to bladder and prostate cancers. Generally, the incidence rate of kidney cancer is high in more developed regions, such as Europe and North America, and low in less developed regions, such as Asia and South America (17). Accordingly, this study also suggested that the ASMR and ASDR burden was also the largest in each year from 1990 to 2019 in high SDI regions. There was a significantly positive correlation between SDI and the ASDR or ASMR attributable to kidney cancer. Racial/ethnic factors and environmental exposures might have a role in the different kidney cancer incident patterns (18). If the trends of kidney cancer incidence, and associated disability and mortality, continue to increase, additional medical and economic resources will be required, especially in low-income countries that lack adequate numbers of appropriately trained urologists and oncologists. Also, due to limited economic resources, most patients will have less access to standard and advanced treatment strategies. For bladder and prostate cancer, although the ASMR and ASDR remained stable or decreased in low and low-middle SDI regions, the curative and palliative care for cancer patients is still limited due to population growth and the ever-increasing incidence rate. The ASMR and ASDR of testicular cancer decreased gradually in high and high-middle SDI regions but they increased in the middle, low-middle, and low SDI regions. This was perhaps because of new therapeutic regimens and the implementation of evidence-based approaches that were widely adopted in higher SDI regions but not in the middle, low-middle, and low SDI regions (19).

Smoking is the main modifiable risk factor for bladder cancer and kidney cancer (20). From 1990 to 2019, decreasing trends for smoking-related bladder, kidney, and prostate cancer mortality were observed in this study. However, smoking was still the leading risk factor for male kidney cancer death and bladder cancer death in all the years evaluated and was the only risk factor for prostate cancer identified in this study. The reduced smoking-related cancer death can be explained by the decreased smoking prevalence worldwide since 1990 (21). Nevertheless, smoking remains the top risk factor for ASMR and ASDR in male kidney cancer all over the world since 1990, so there is still a vast base of smokers and one in four men worldwide smokes every day. Efforts to reduce smoking and their effectiveness also differ between countries and regions and may be affected by tobacco industry targeting (22–24). There is still much work to be done in reducing tobacco consumption and cancer prevention. Additional tobacco control and education on smoking cessation should be made to avoid adolescent initiation of smoking, as high rates still exist in this population in many countries (21).

Previous studies have reported obesity and a high BMI to be risk factors for kidney cancer incidences and mortality (25, 26). Compared with people without obesity, the risk of kidney cancer increases by 1.32-fold in those with general and abdominal obesity (27). In this study, the kidney cancer mortality rate attributable to high BMI has been increasing and persists as the leading risk factor for female kidney cancer deaths since 1999. In 2019, the contribution of a high BMI to kidney cancer death in both sexes even exceeded that of smoking. The high incidence mortality rate in high SDI regions might also be explained by the high proportion of people with obesity. As the obese population continues to grow worldwide (28), the prevention and management of kidney cancer will be more difficult. Obesity also contributed to bladder cancer incidence and related death (29, 30). A gradual increase was observed in high fasting plasma glucose-associated bladder cancer mortality since 1990. Maintenance of metabolic health could be a reasonable strategy to reduce the incidence and burden of genitourinary cancers.

To our knowledge, this is the most updated estimate on genitourinary cancers epidemiology globally, which includes 204 countries and some that have not been assessed before. Furthermore, the risk factors attributable to kidney cancer and bladder cancer in different sexes were revealed for the first time. It is worth noticing that metabolic health plays an important role in the prevention of kidney cancer and bladder cancer, especially for females. However, this study has some limitations which are common for all GBD burden estimates (8, 9). The major limitation is the availability and completeness of the source data. In addition, the source data were lacking in some regions and were predicted by covariates or trends from neighboring locations, leading to discrepant accuracy of estimates among different countries. Finally, the estimates in the most recent years also had large uncertainty due to lag in data availability, reflected in the broad uncertainty intervals of many estimates, which decreases the reliability of calculated incidences and burdens of specific cancers (31).

## 5. Conclusions

The past thirty years have seen an increased incidence of bladder, kidney, prostate, and testicular cancer, as well as increased disability and mortality to kidney cancer. High-income regions experience higher incidences and burdens attributable to these four cancers. The mortality rate of kidney, bladder, and prostate cancer increases with age and the majority of deaths are seen in elderly people, with smoking and obesity being the major causes. Smoking is still the leading risk factor for mortality for bladder, kidney, and prostate cancers, however, the role of obesity in genitourinary cancer mortality is becoming more and more important. Thus, additional tobacco control and education on smoking cessation, as well as healthy lifestyles, should be provided. Finally, the maintenance of metabolic health could be a reasonable strategy to reduce the incidence and burden of genitourinary cancers.

## Data availability statement

The original contributions presented in the study are included in the article/Supplementary material, further inquiries can be directed to the corresponding authors.

## Author contributions

D-WY had full access to all the data in the study, takes responsibility for the integrity of the data, and the accuracy of the data analysis. Study concept and design: RD and D-WY. Acquisition of data: J-CY and J-JH. Analysis and interpretation of data: Y-QT and J-CY. Drafting of the manuscript: Y-QT, J-JH, and J-CY. Critical revision of the manuscript for important intellectual content: J-JH, RD, and J-WS. Statistical analysis: Y-QT and J-WS. Supervision: D-WY and J-WS. Contributed equally to this work as co-first author: Y-QT, J-CY, J-JH, and RD. All authors contributed to the article and approved the submitted version.
