# Supplementary figures and images for: Trends and risk factors of global incidence, mortality, and disability of genitourinary cancers from 1990 to 2019: Systematic analysis for the Global Burden of Disease Study 2019
Source: Front Public Health. 2023 Feb 22;11:1119374. doi: 10.3389/fpubh.2023.1119374 (PMC9992434; doi:10.3389/fpubh.2023.1119374)

A

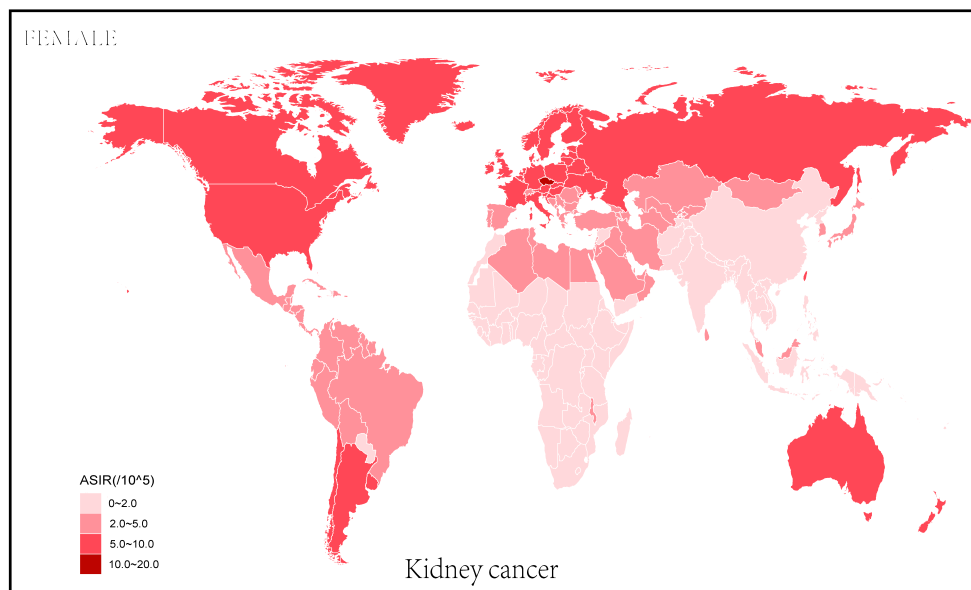

B

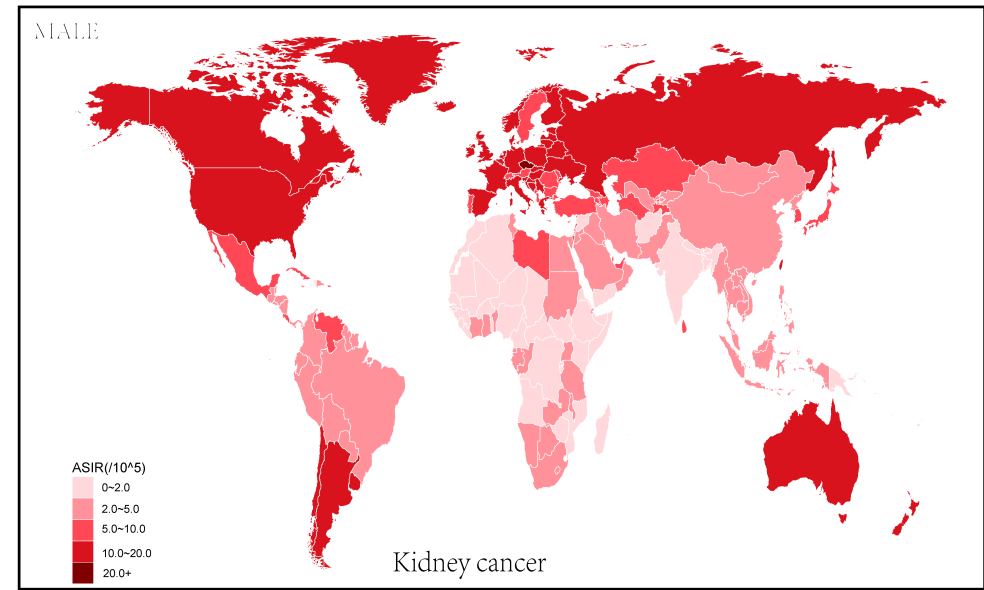

C

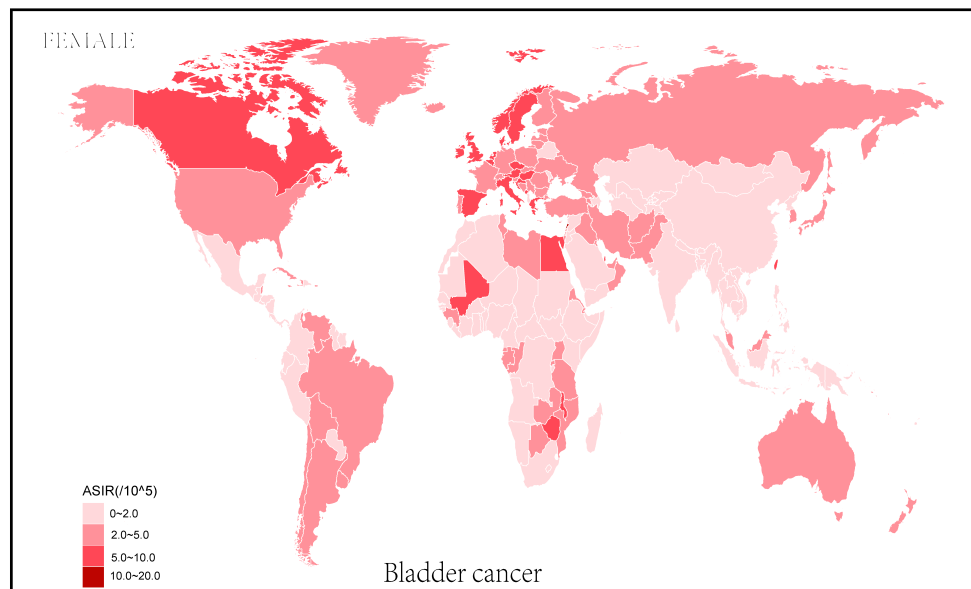

D

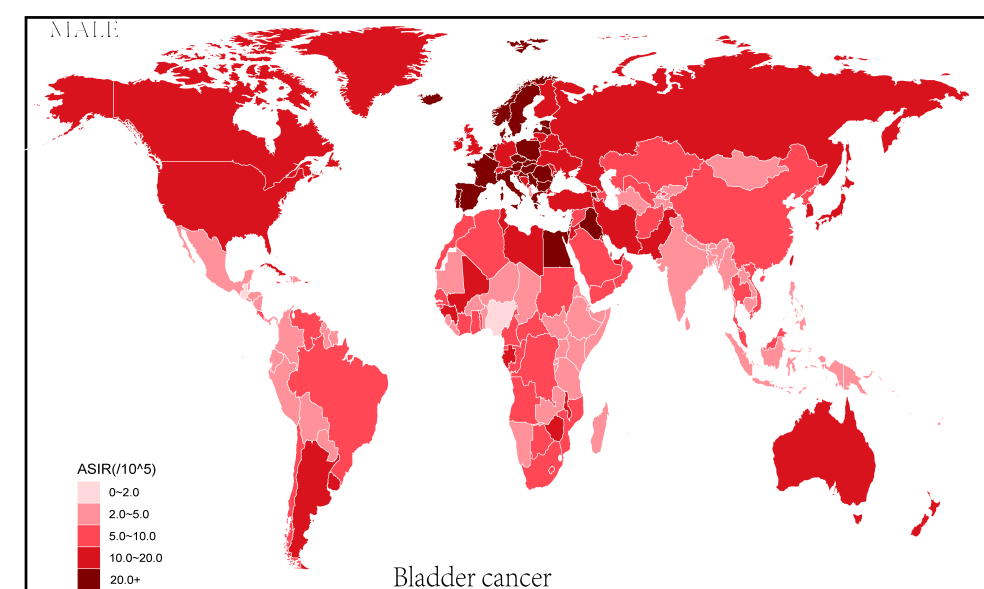

Supplement: Supplementary file 1 [file Image_1.pdf]

A

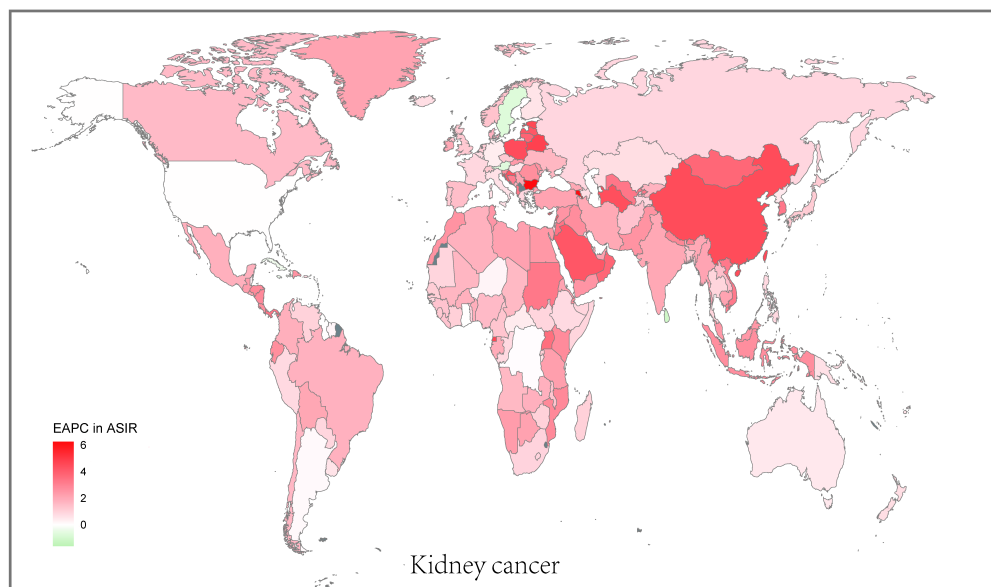

B

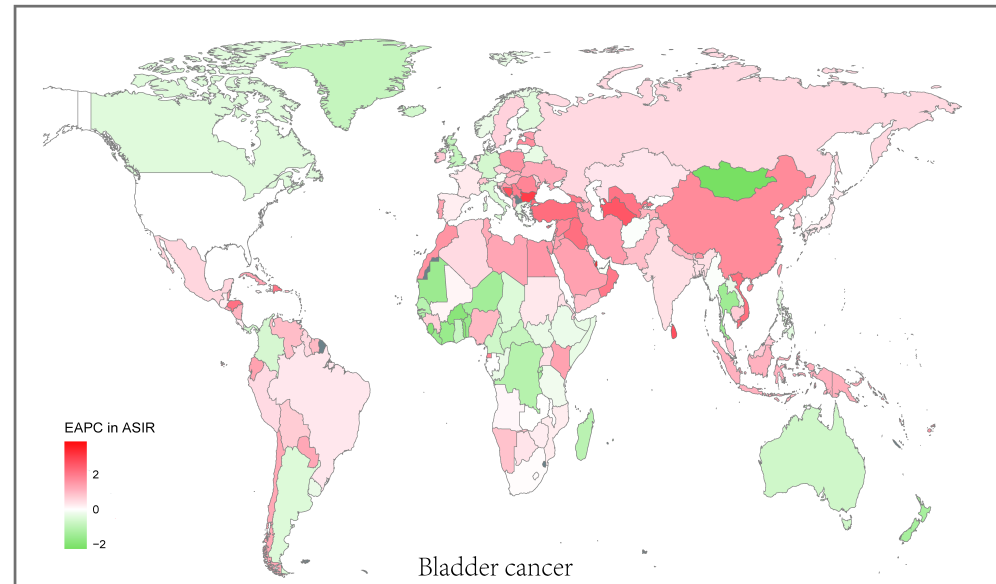

C

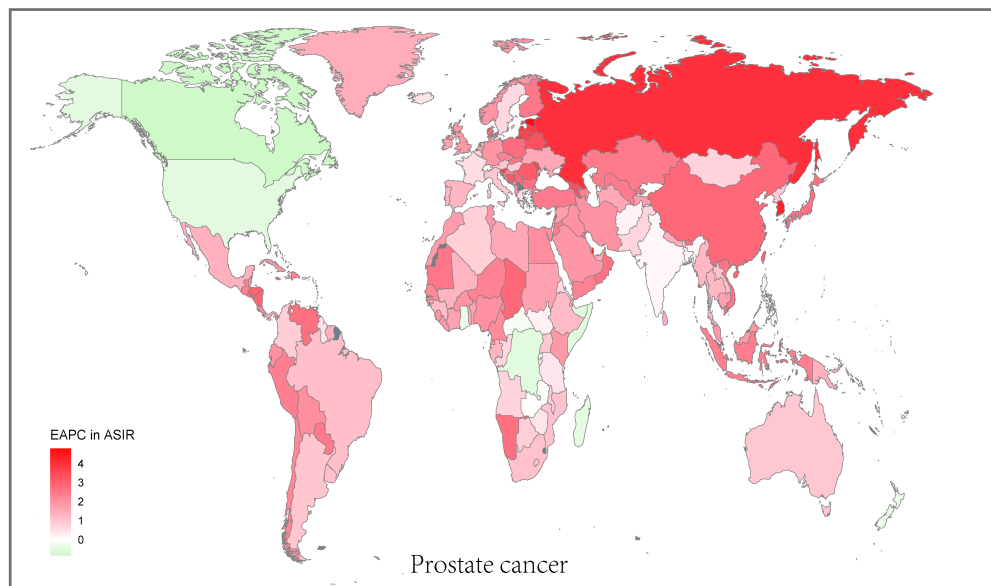

D

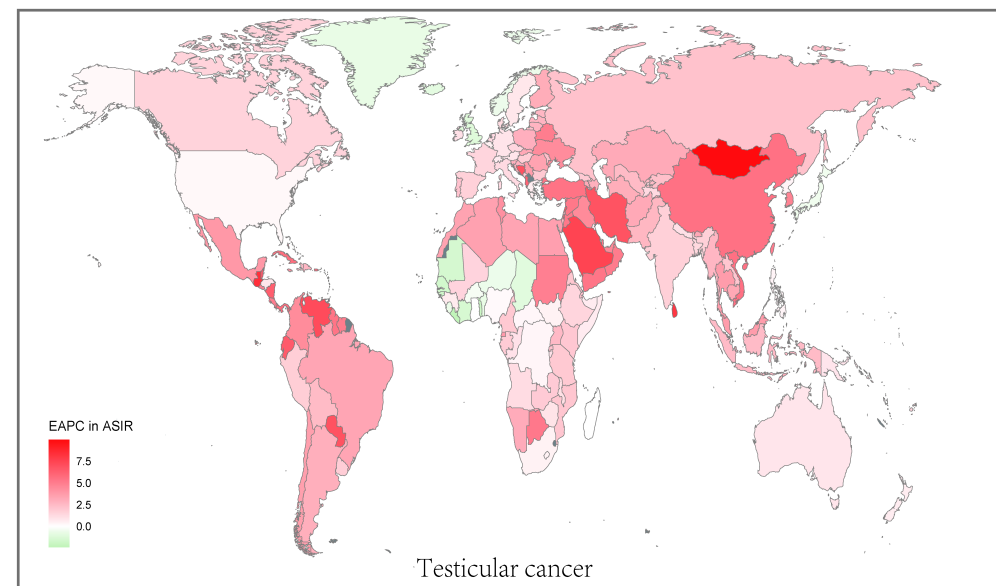

Supplement: Supplementary file 2 [file Image_2.pdf]

A

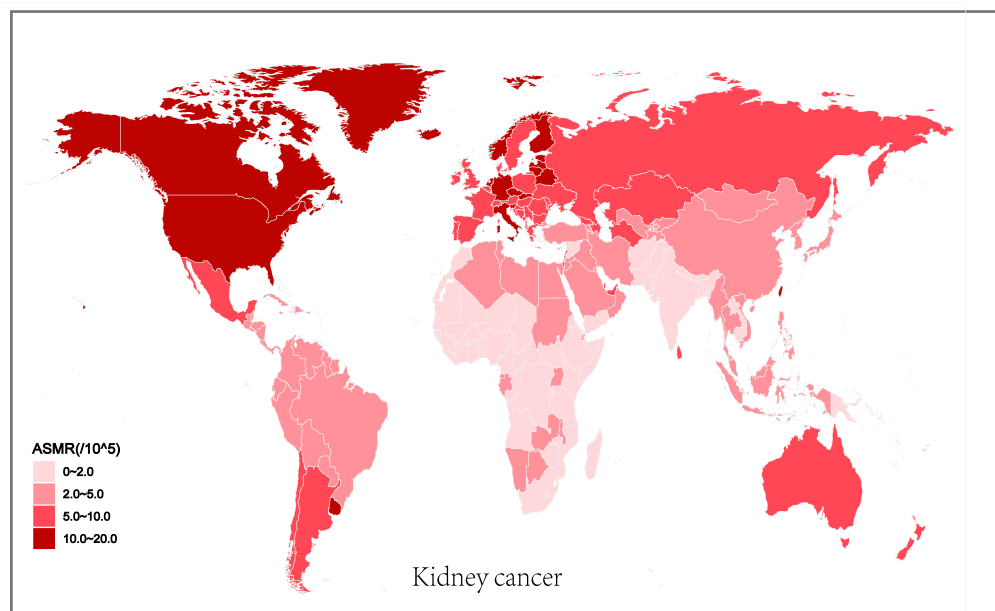

B

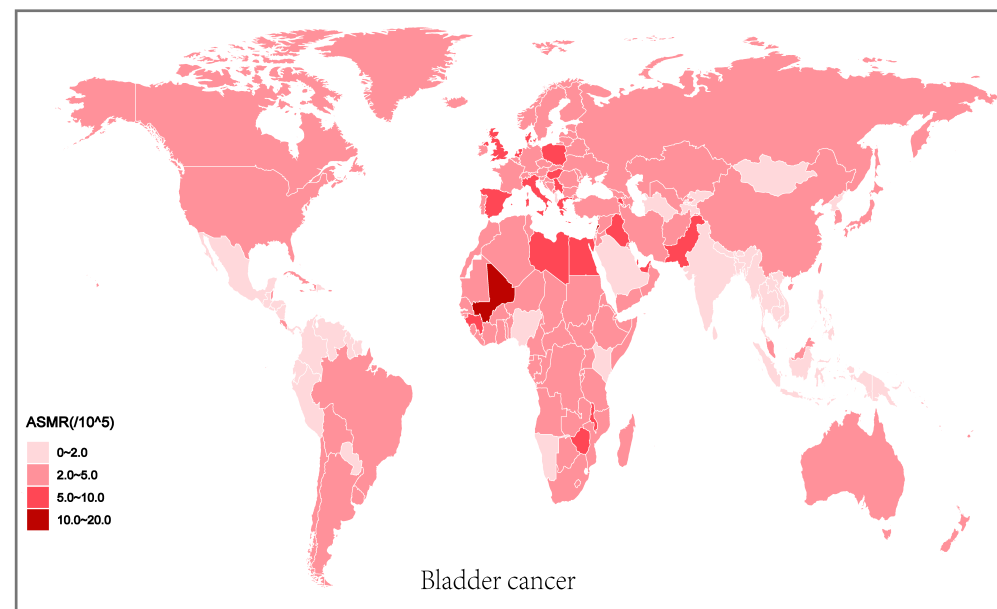

C

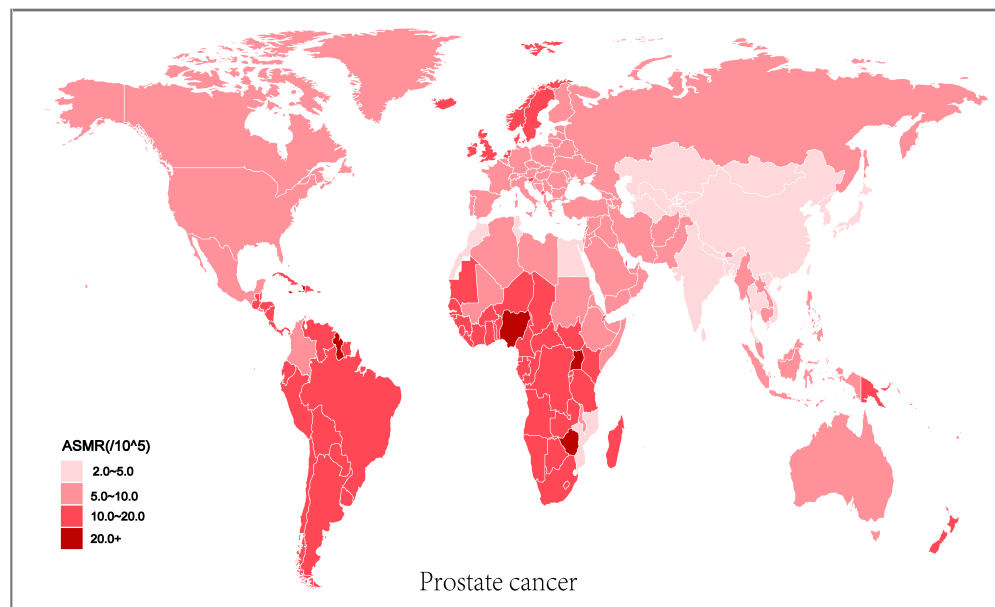

D

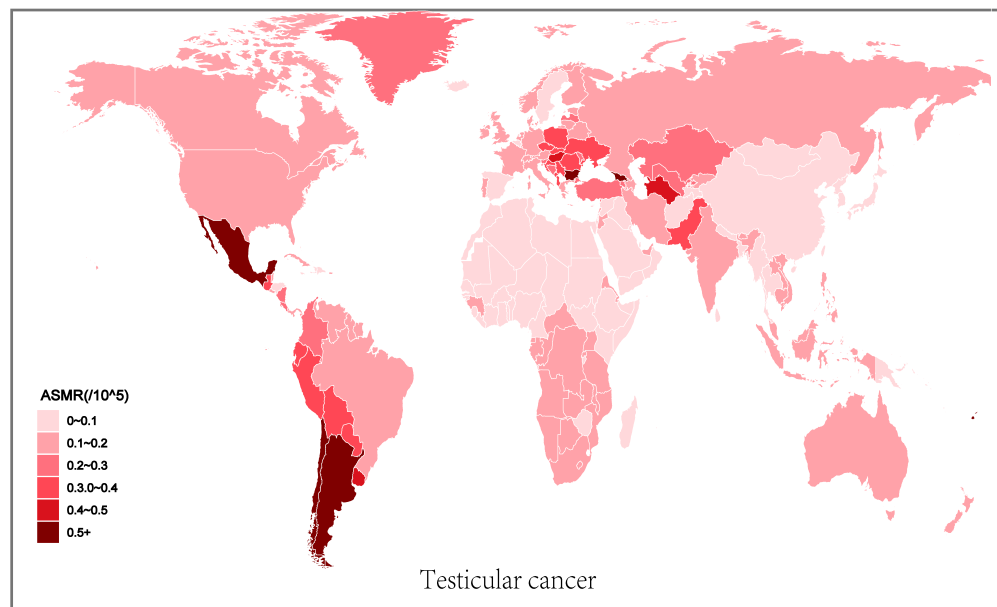

Supplement: Supplementary file 3 [file Image_3.pdf]

A

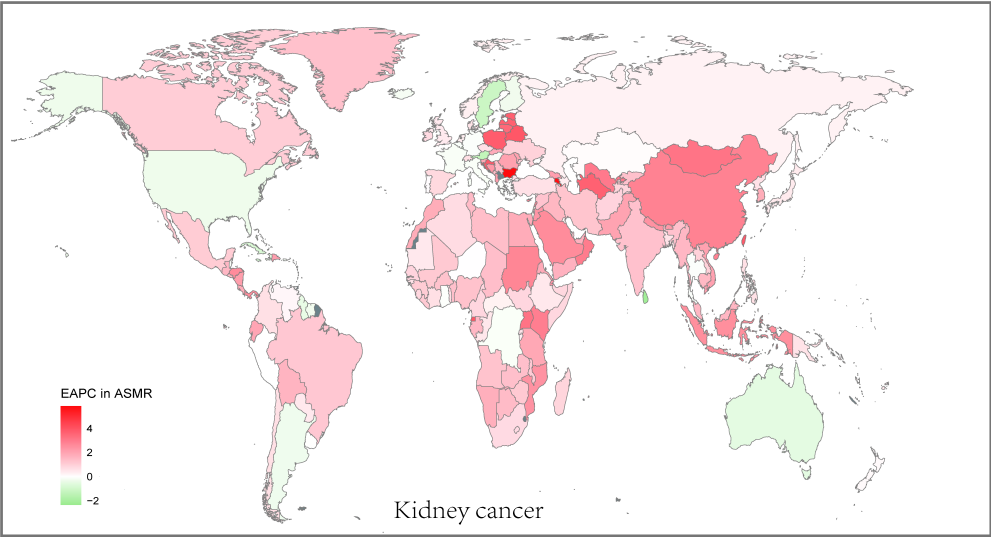

B

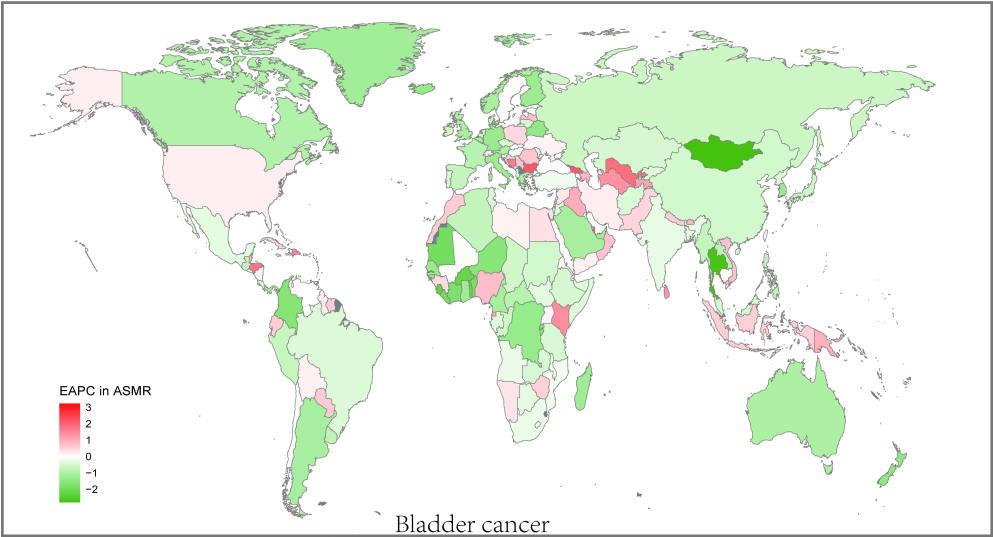

C

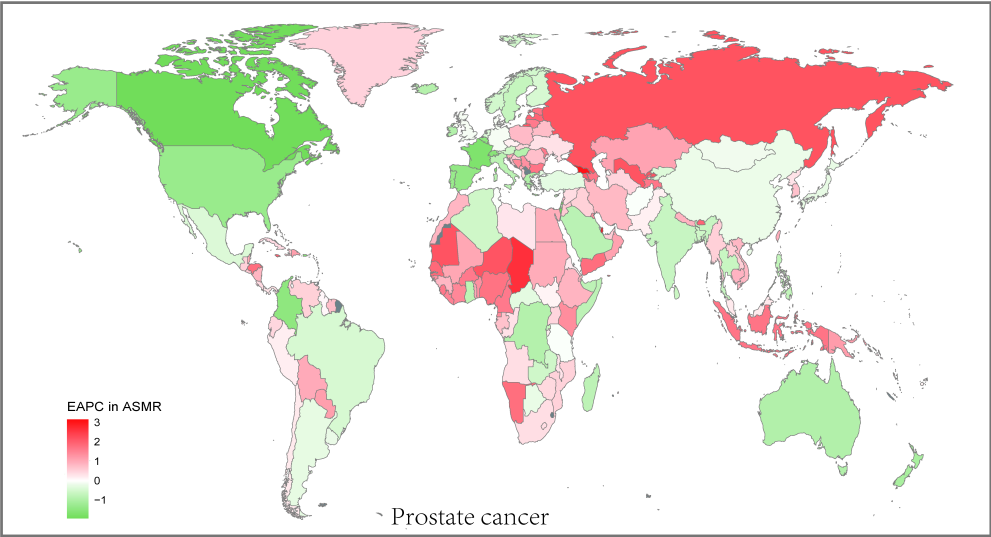

D

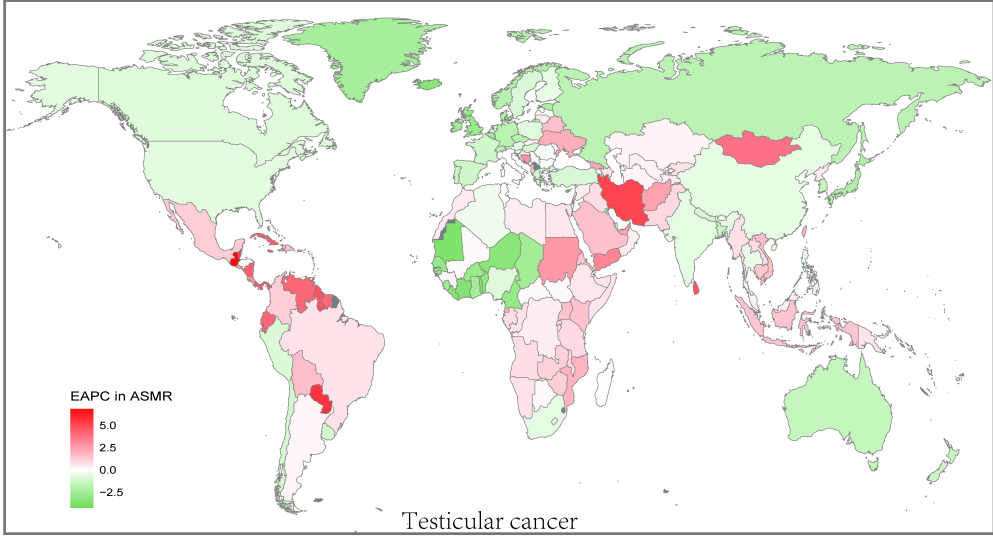

Supplement: Supplementary file 4 [file Image_4.pdf]

A

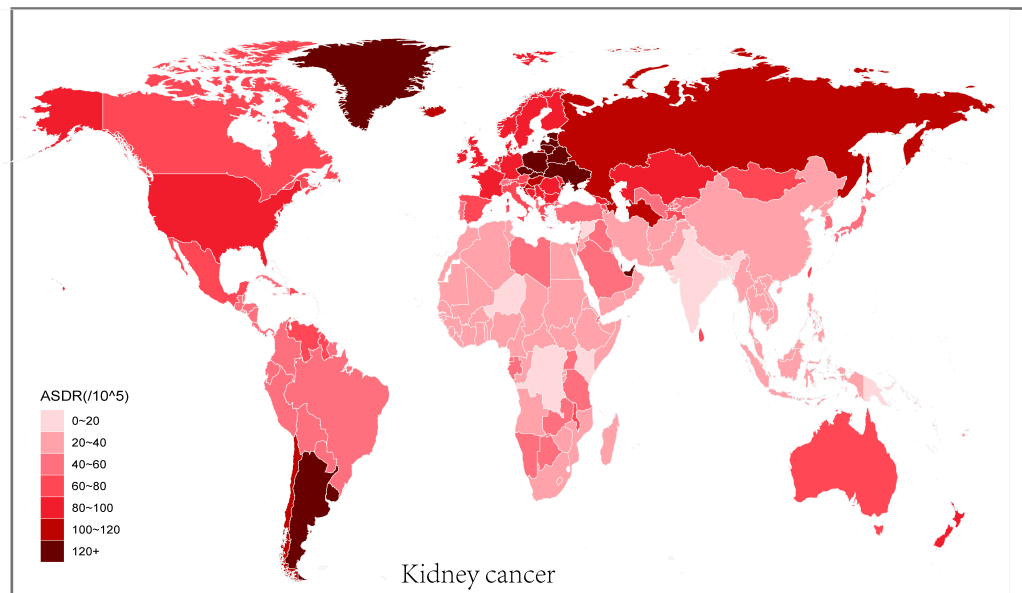

B

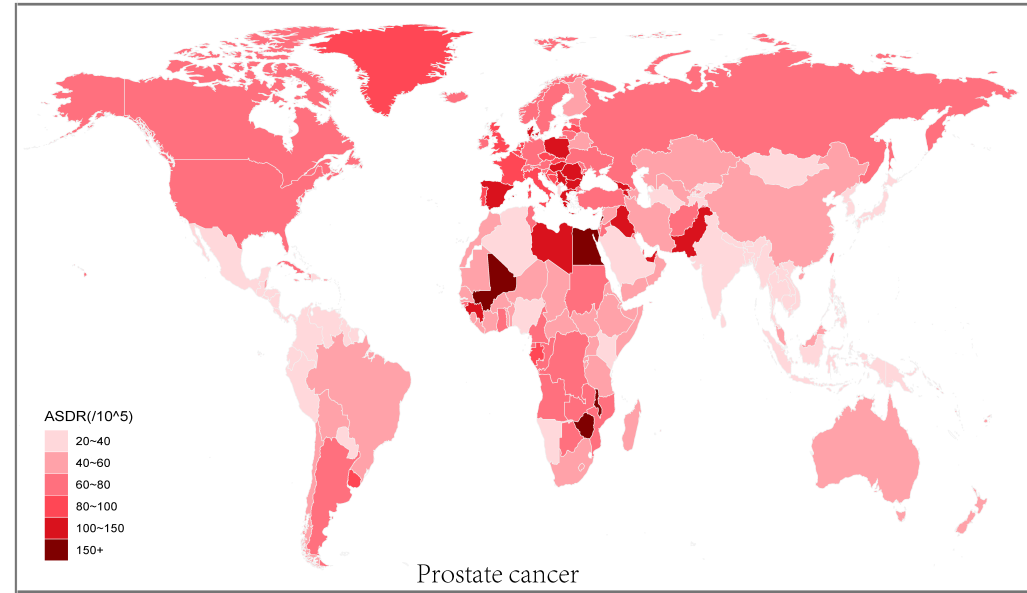

C

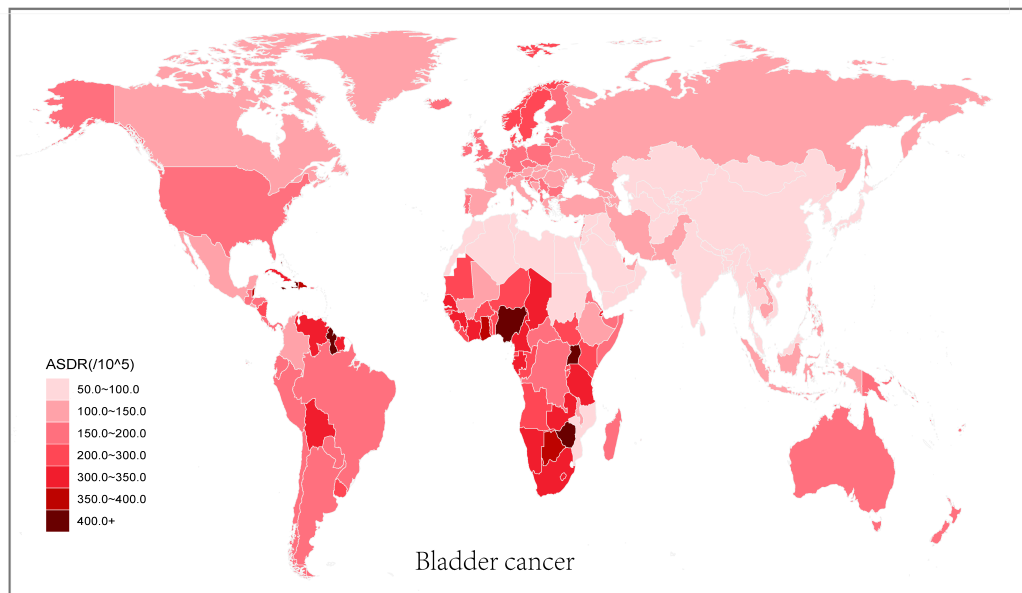

D

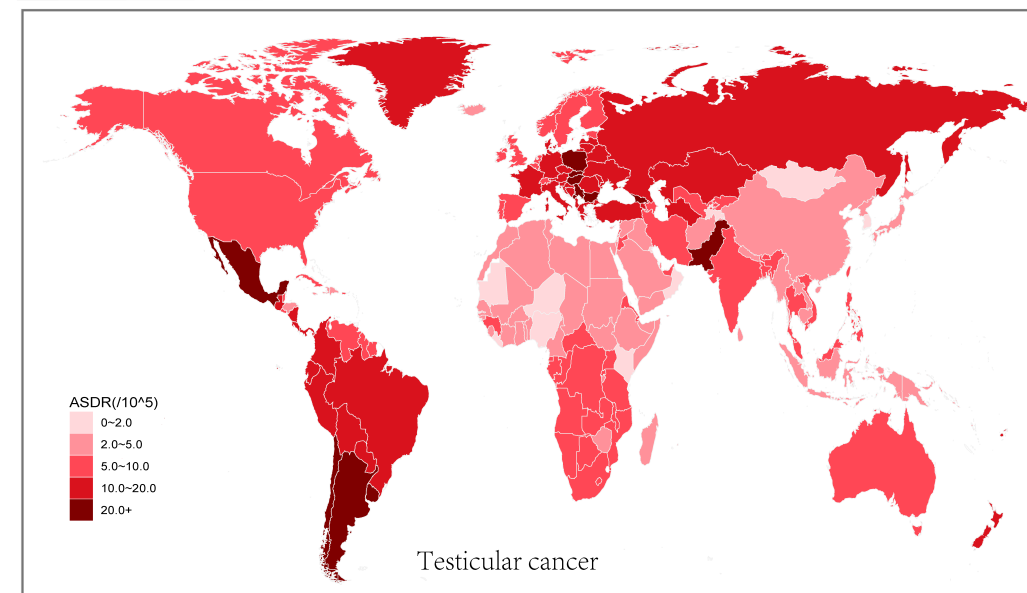

Supplement: Supplementary file 5 [file Image_5.pdf]

A

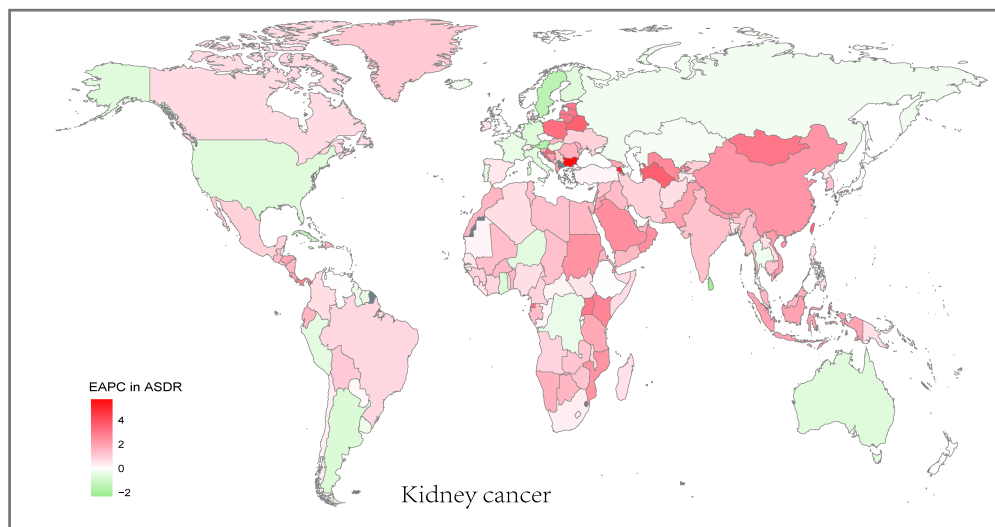

B

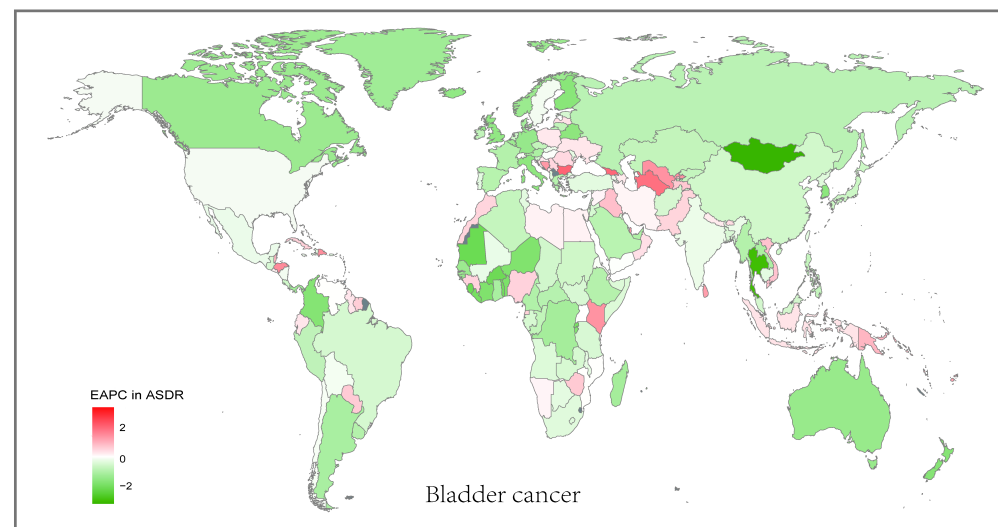

C

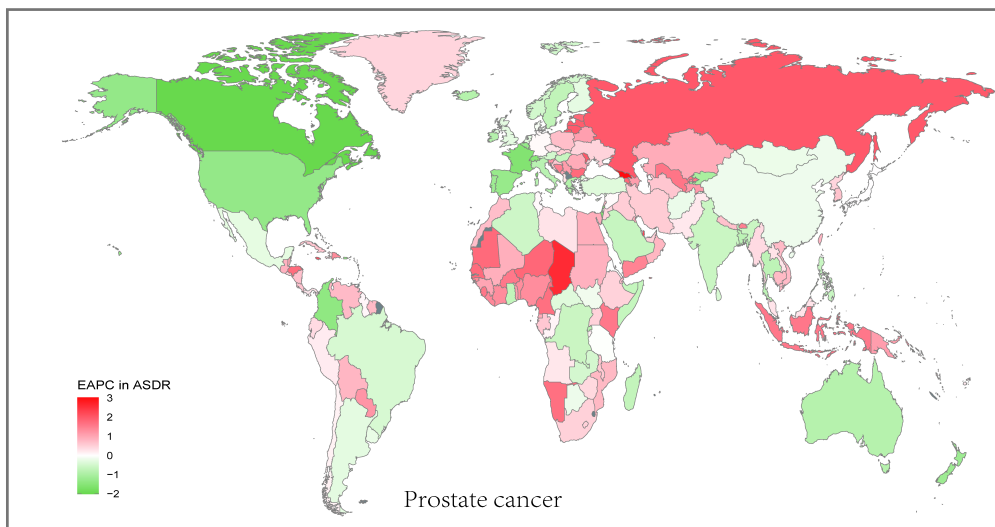

D

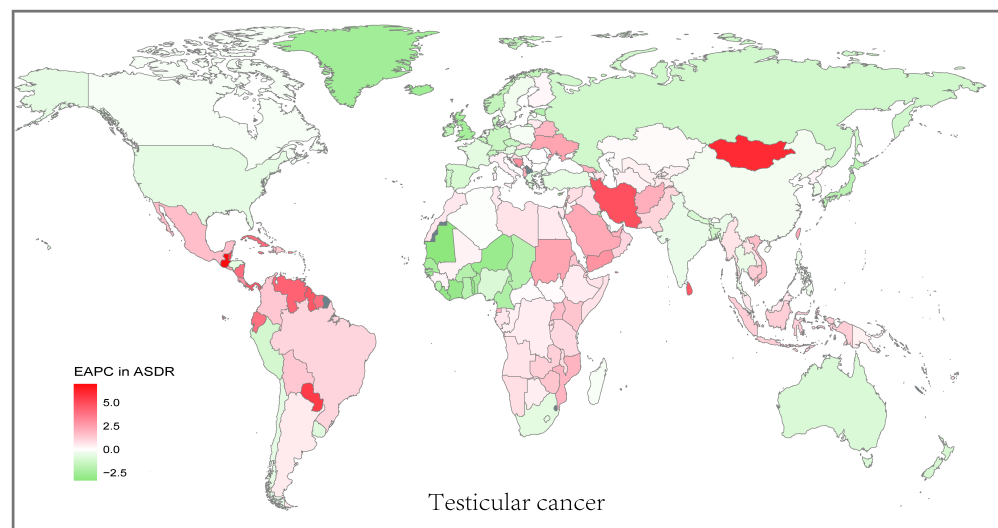

Supplement: Supplementary file 6 [file Image_6.pdf]

A

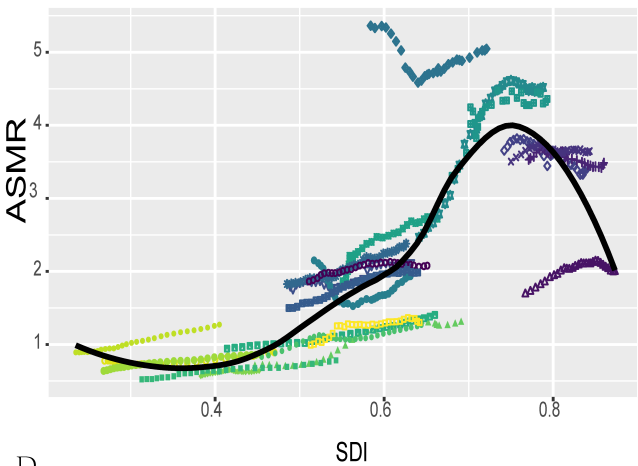

B

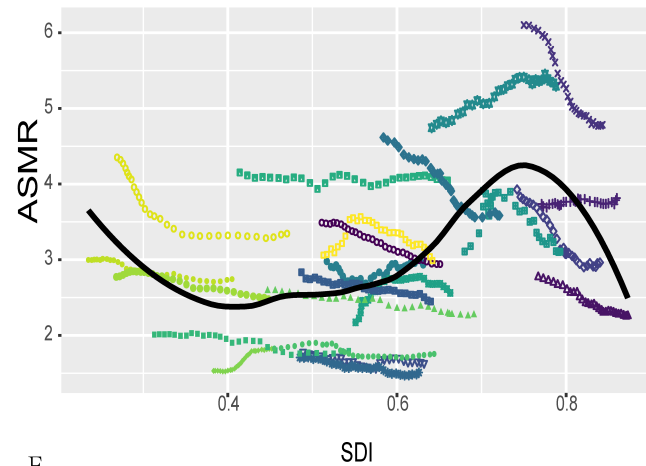

C

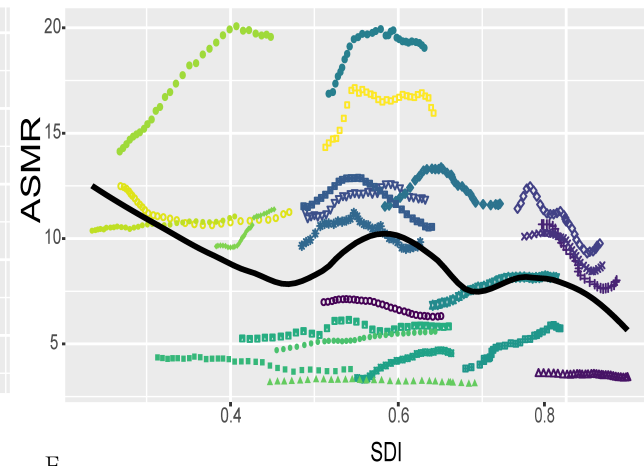

D

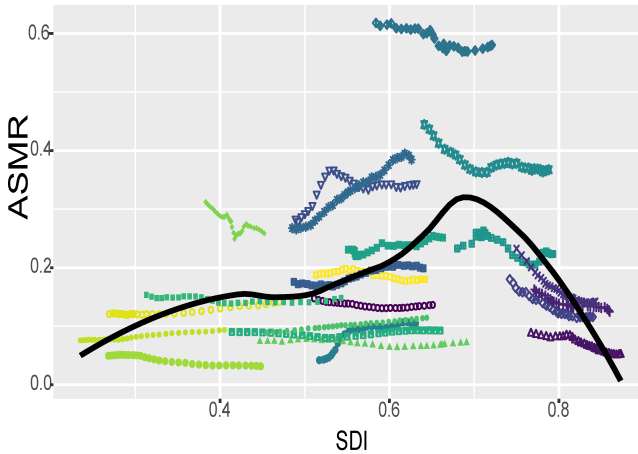

E

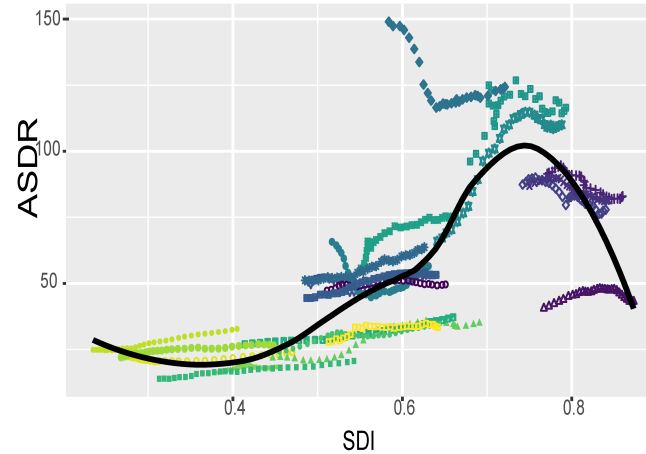

F

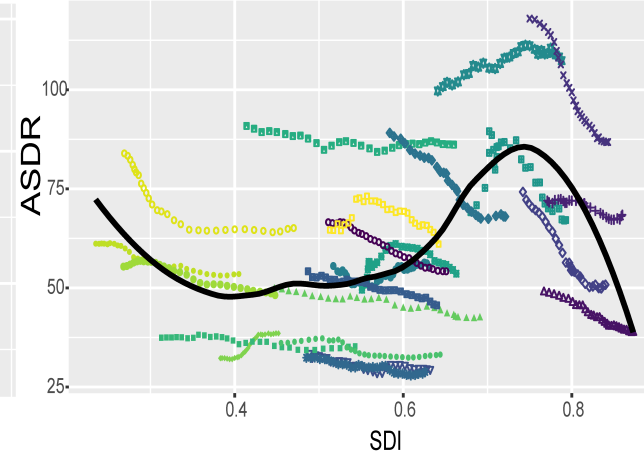

G

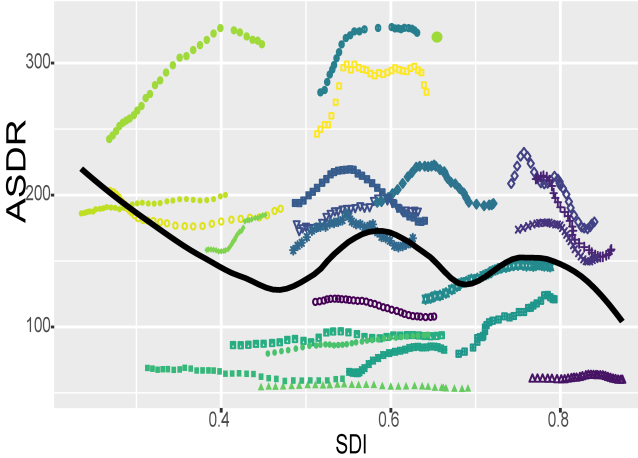

H

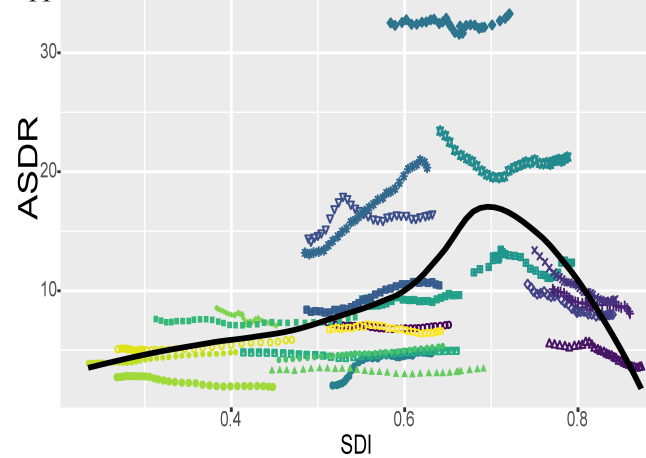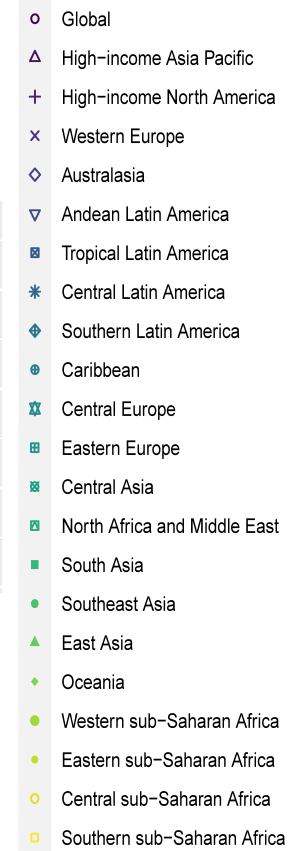

Supplement: Supplementary file 7 [file Image_7.pdf]

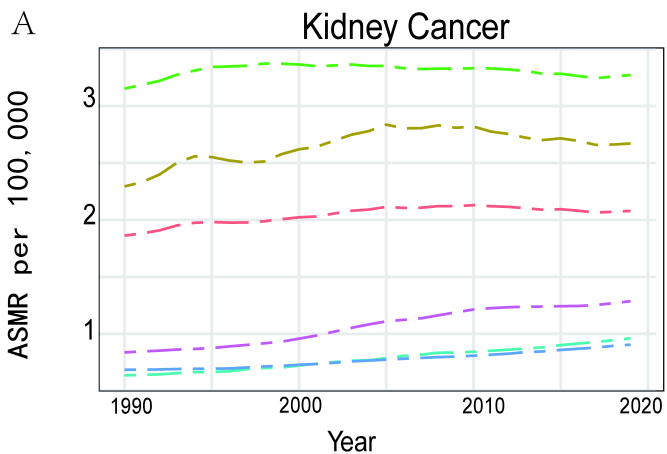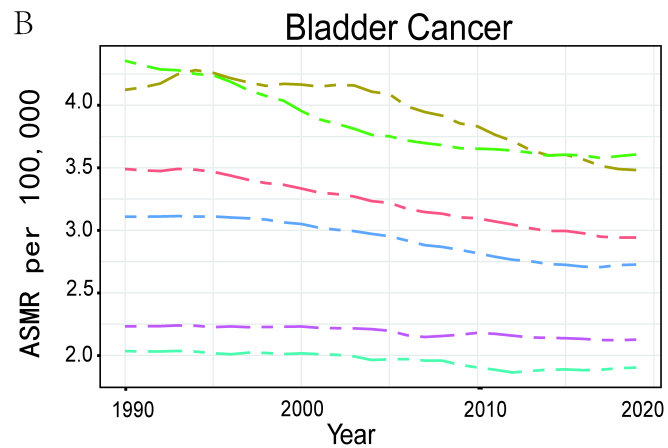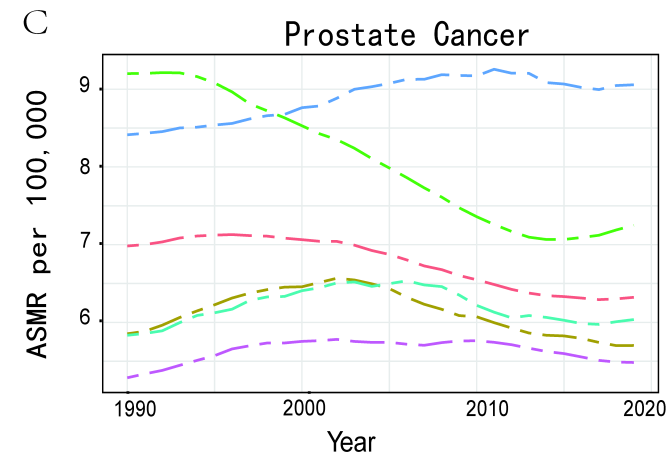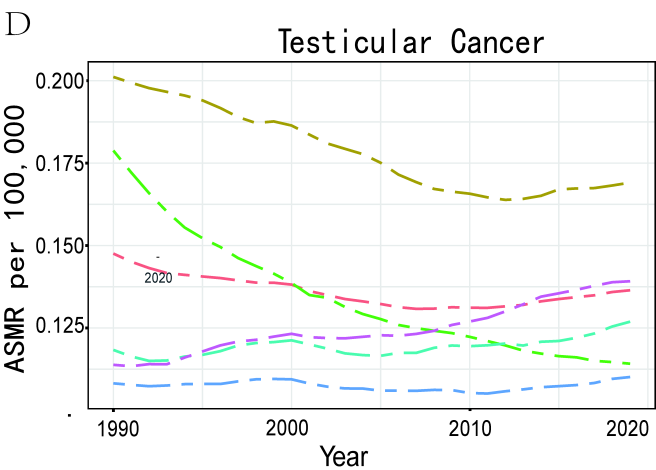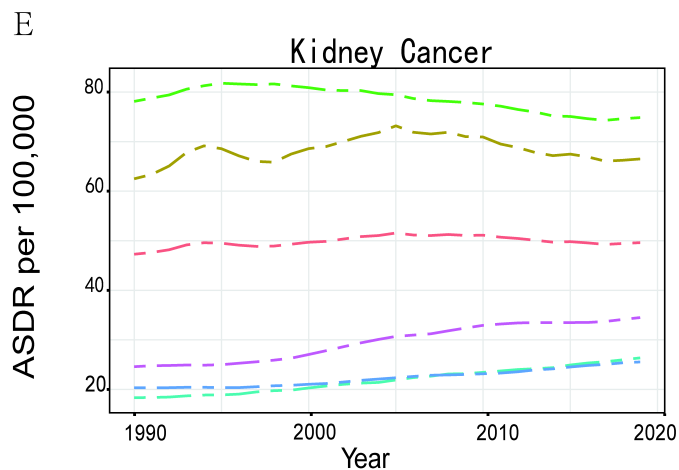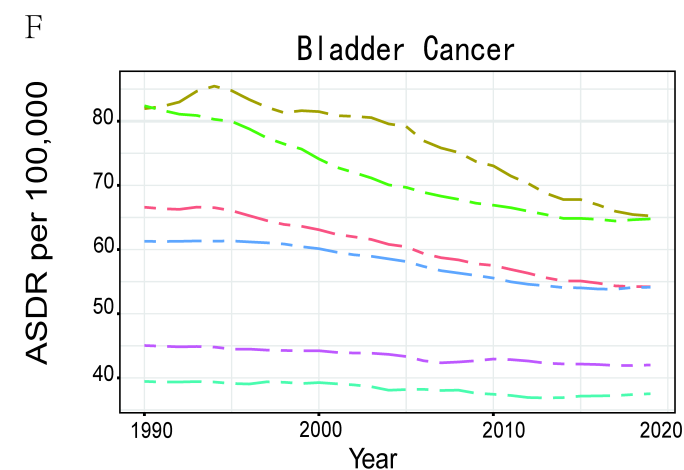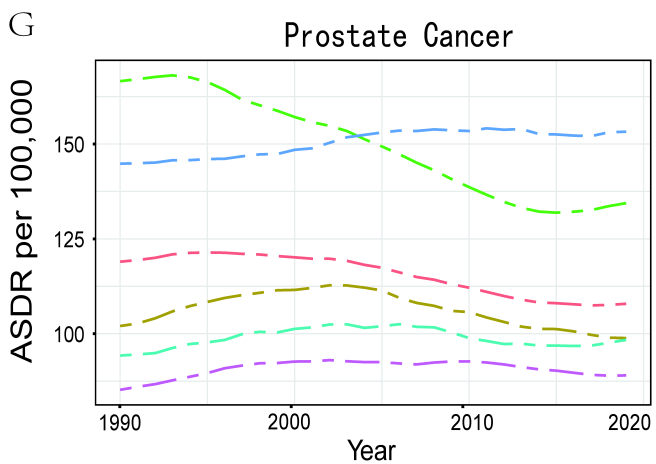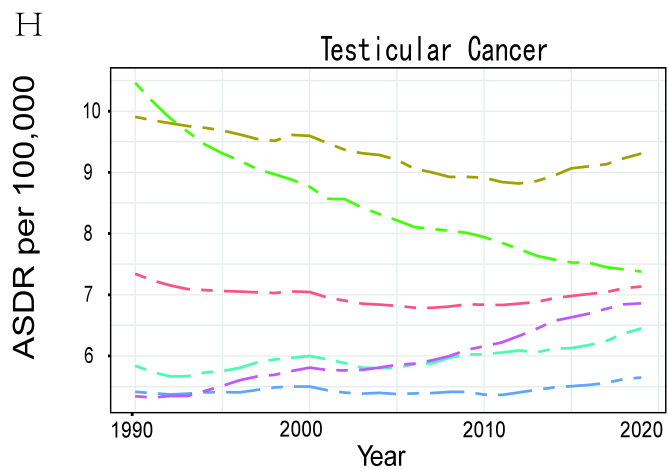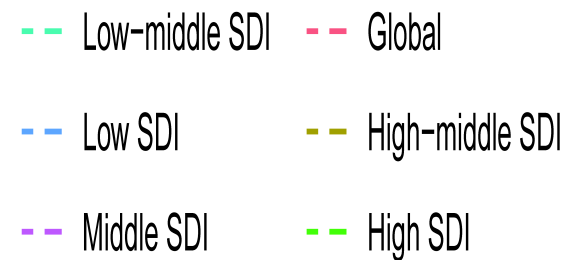

Supplement: Supplementary file 8 [file Image_8.pdf]

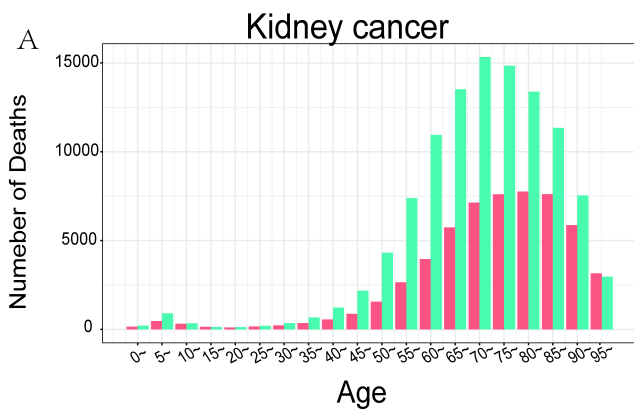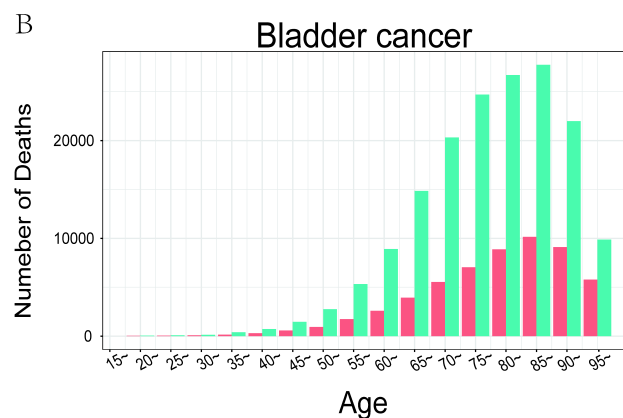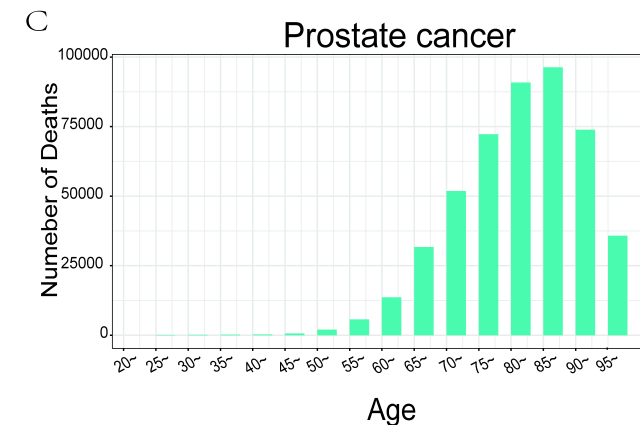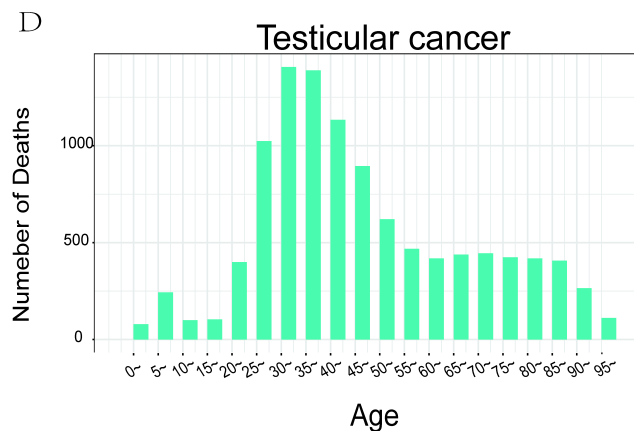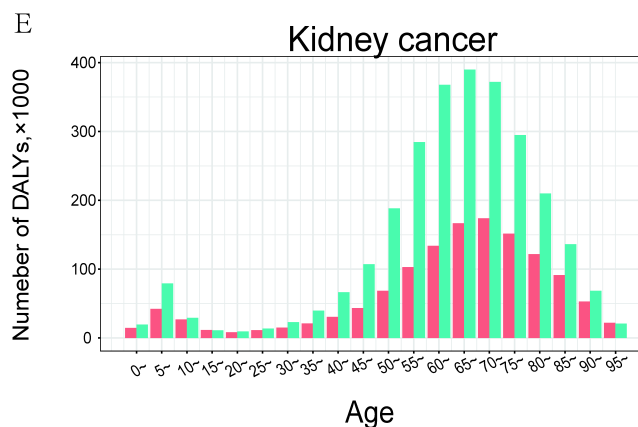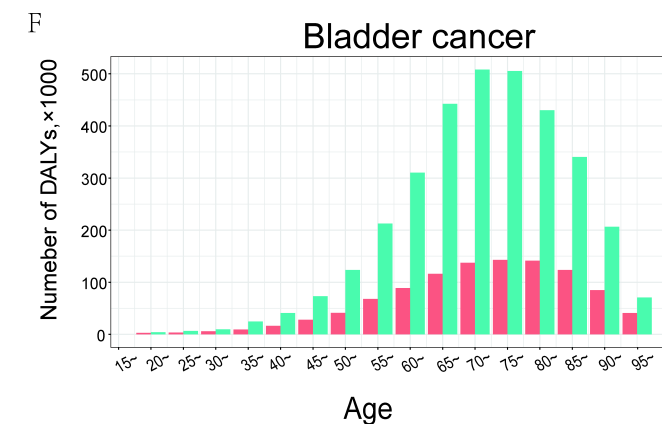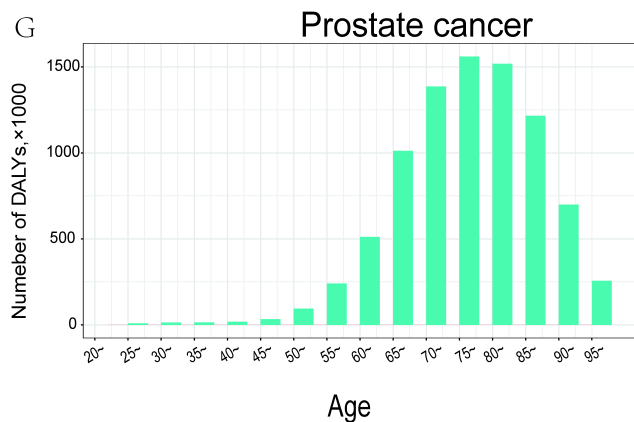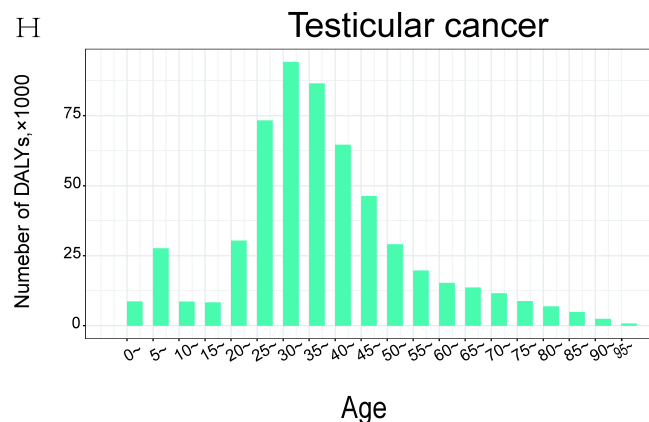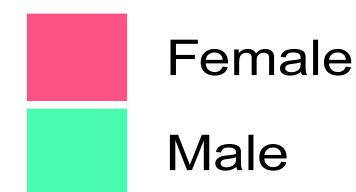

Supplement: Supplementary file 9 [file Image_9.pdf]

A

## Kidney cancer

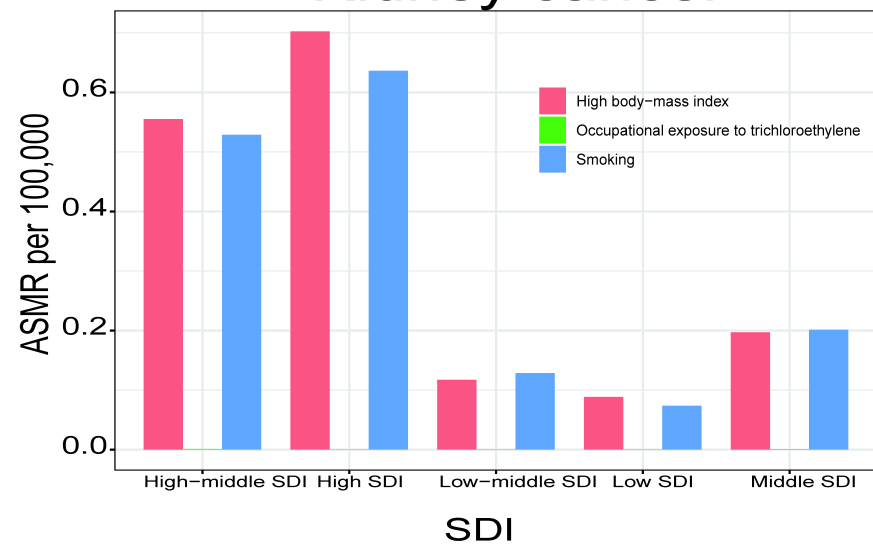

B

## Bladder cancer

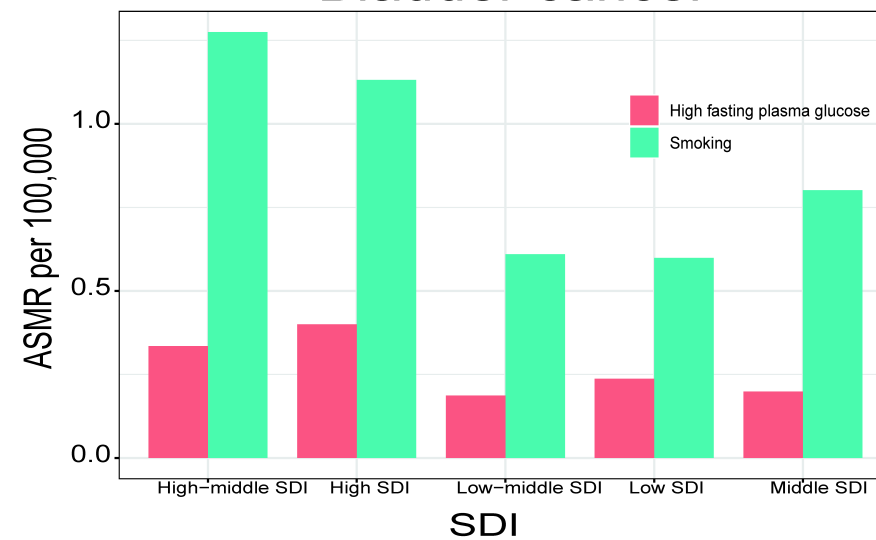

C

## Prostate cancer

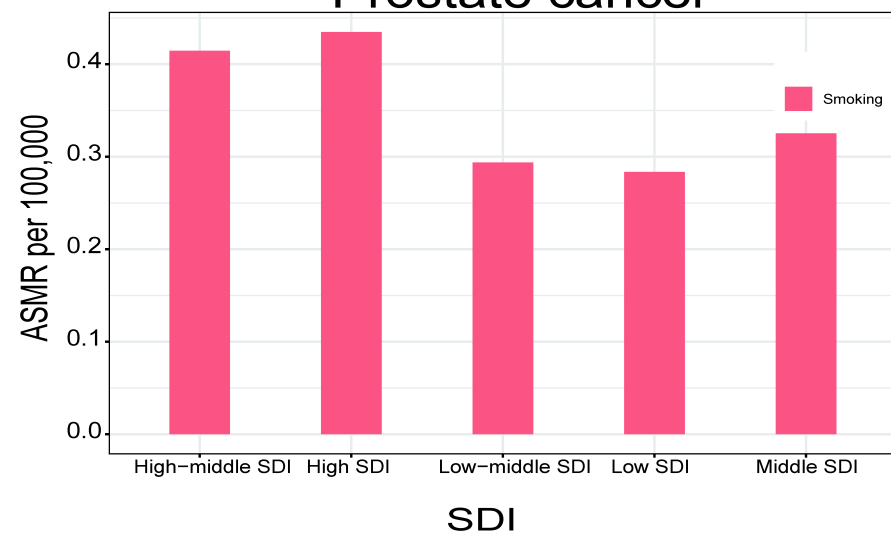

Supplement: Supplementary file 10 [file Image_10.pdf]

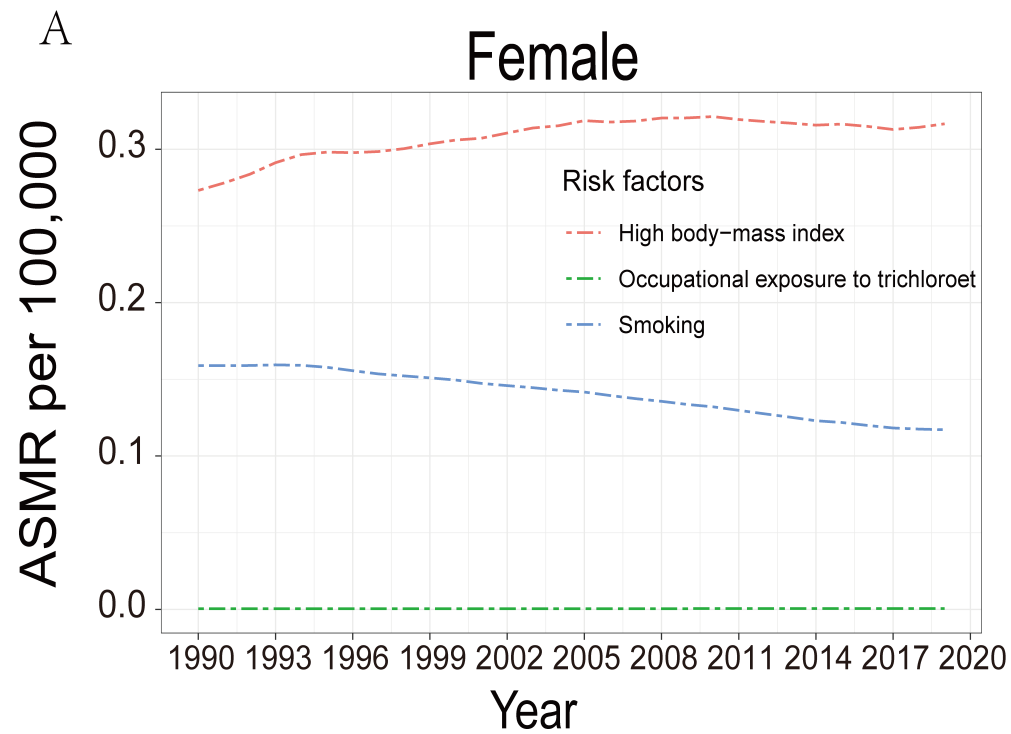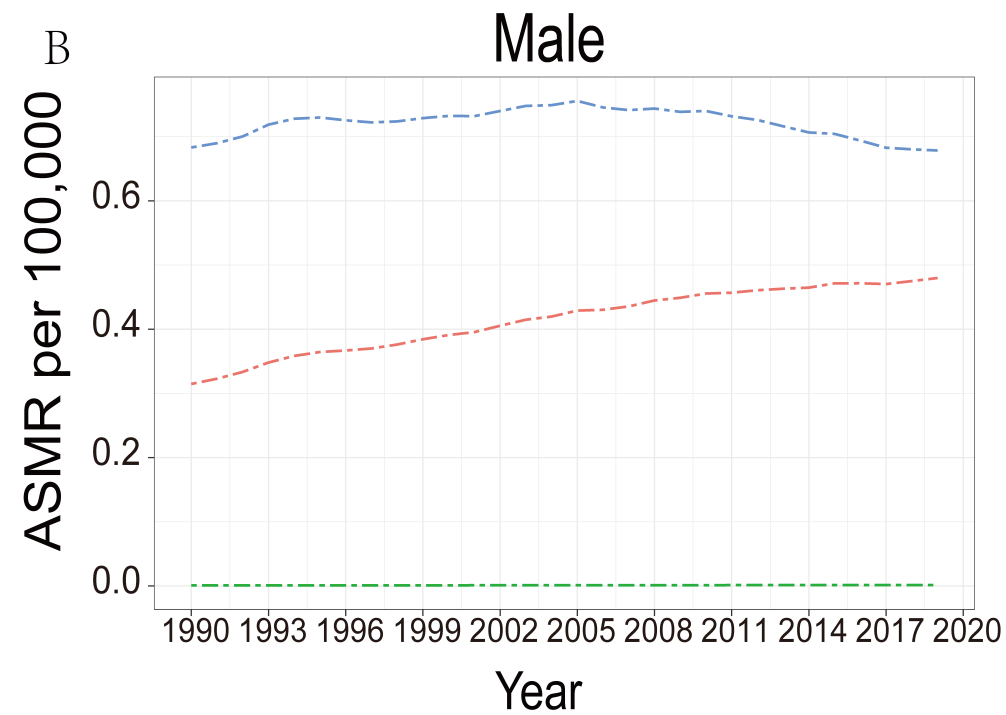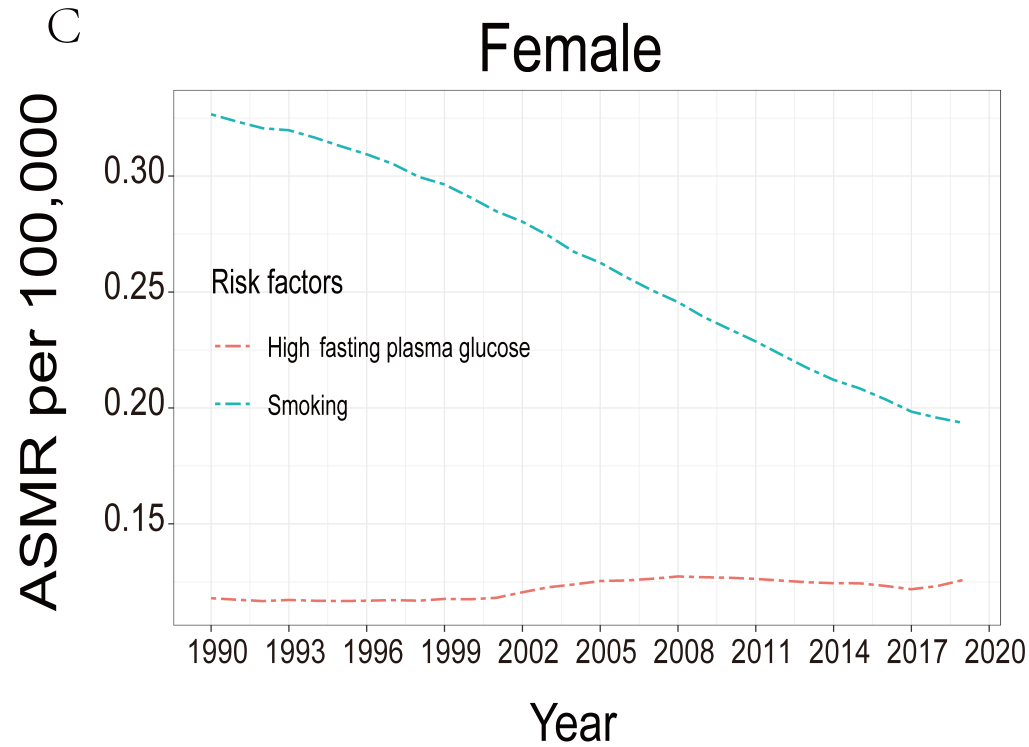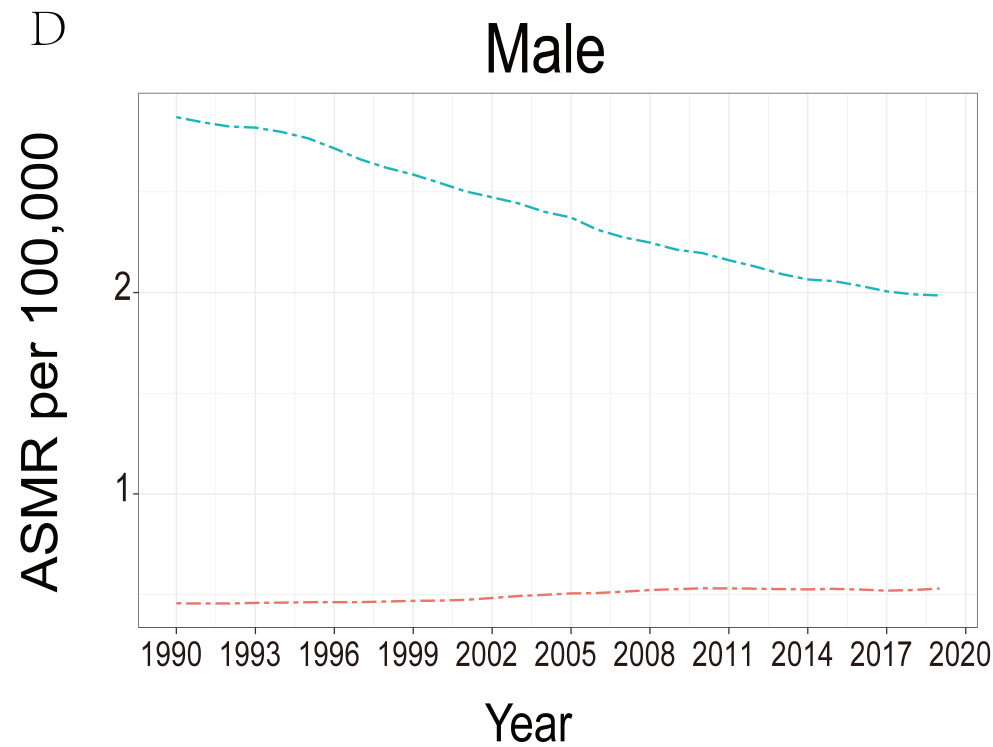

Supplement: Supplementary file 11 [file Image_11.pdf]
