# Supplementary material for: Trends and risk factors of global incidence, mortality, and disability of genitourinary cancers from 1990 to 2019: Systematic analysis for the Global Burden of Disease Study 2019
Source: Front Public Health. 2023 Feb 22;11:1119374. doi: 10.3389/fpubh.2023.1119374 (PMC9992434; doi:10.3389/fpubh.2023.1119374)
Supplement: Supplementary file 12 [file Table_1.DOCX]

Table 1S. Genitourinary cancers included in GBD 2019 and their ICD-10 codes

|  | ICD-10 Codes | conditions |
| --- | --- | --- |
| Kidney cancer | C64-C65.9 | Malignant neoplasm of kidney and renal pelvis |
|  | D30.0-D30.1 | Benign neoplasm of kidney and renal pelvis |
|  | D41.0-D41.1 | Neoplasm of kidney and renal pelvis |
| Bladder cancer | C67-C67.9 | Malignant neoplasm of bladder |
|  | D09.0 | Carcinoma in situ of bladder |
|  | D30.3 | Benign neoplasm of bladder |
|  | D41.4-D41.8 | Neoplasm of bladder |
|  | D49.4 | - |
| Prostate cancer | C61-C61.9 | Malignant neoplasm of prostate |
|  | D07.5 | Carcinoma in situ of prostate |
|  | D29.1 | Benign neoplasm of prostate |
|  | D40.0 | Neoplasm of prostate |
| Testicular cancer | C62-C62.9 | Malignant neoplasm of testis |
|  | D29.2-D29.8 | Benign neoplasm of testis, epididymis, scrotum, seminal vesicle, spermatic cord and tunica vaginalis |
|  | D40.1-D40.8 | Neoplasm of testis and Skin of male genital organs |

**Abbreviations:** GBD: Global Burden and Disease Study 2019; ICD-10: International Statistical Classification of Diseases and Related Health Problems 10th Revision
