# Supplementary material for: Trends and risk factors of global incidence, mortality, and disability of genitourinary cancers from 1990 to 2019: Systematic analysis for the Global Burden of Disease Study 2019
Source: Front Public Health. 2023 Feb 22;11:1119374. doi: 10.3389/fpubh.2023.1119374 (PMC9992434; doi:10.3389/fpubh.2023.1119374)
Supplement: Supplementary file 13 [file Table_2.DOCX]

| Table 2S. Death number, age standardized mortality rate in 2019 and the percentage change of mortality rate for bladder, kidney, prostate and testicular cancer between 1990 and 2019 | | | | | | | | | | | | | | | | |
| --- | --- | --- | --- | --- | --- | --- | --- | --- | --- | --- | --- | --- | --- | --- | --- | --- |
|  | | Kidney cancer | | | Bladder cancer | | | | | Prostate cancer | | | Testicular cancer | | | |
|  | Death number (×1000) in 2019 | | Age standardized mortality rate per 100,000 in 2019 | Change of age standardized mortality rate between 1990 and 2019 | | Death number (×1000) in 2019 | Age standardized mortality rate per 100,000 in 2019 | Change of age standardized mortality rate between 1990 and 2019 | Death number (×1000) in 2019 | | Age standardized mortality rate per 100,000 in 2019 | Change of age standardized mortality rate between 1990 and 2019 | | Death number (×1000) in 2019 | Age standardized mortality rate per 100,000 in 2019 | Change of age standardized mortality rate between 1990 and 2019 |
| Global |  | |  |  | |  |  |  |  | |  |  | |  |  |  |
| Both sexes | 166.44 (155.46 to 176.3) | | 2.08 (1.93 to 2.2) | 11.61% (4.64% to 19.98%) | | 228.73 (210.74 to 243.19) | 2.94 (2.7 to 3.13) | -15.71% (-21.04% to -8.59%) | 486.84 (420.5 to 593.69) | | 6.32 (5.42 to 7.71) | -9.46% (-15.19% to -0.05%) | | 10.8 (10 to 12) | 0.14 (0.13 to 0.15) | -7.53%(-15.88% to 1.35%) |
| Females | 57.67 (52.24 to 61.9) | | 1.33 (1.2 to 1.42) | -2.29% (-8.24% to 4.63%) | | 59.53 (52.33 to 64.58) | 1.36 (1.19 to 1.47) | -20.02% (-27.27% to -12.43%) | / | | / | / | | / | / | / |
| Males | 108.77 (101.48 to 115.82) | | 2.99 (2.78 to 3.18) | 19.35% (10.46% to 30.02%) | | 169.21 (156.92 to 180.66) | 5.09 (4.69 to 5.44) | -16.96% (-22.54% to -9.35%) | 486.84 (420.5 to 593.69) | | 15.28 (13 to 18.57) | -15.72% (-20.85% to -7.61%) | | 10.8 (10 to 12) | 0.14 (0.13 to 0.15) | -7.53%(-15.88% to 1.35%) |
| Region |  | |  |  | |  |  |  |  | |  |  | |  |  |  |
| Western Sub-Saharan Africa | 2.05 (1.58 to 2.57) | | 0.86 (0.71 to 1.02) | 34.43% (5.6% to 77.38%) | | 3.81 (2.66 to 4.52) | 2.48 (1.74 to 2.91) | -10.59% (-43.44% to 14.31%) | 26.3 (14.73 to 35.42) | | 19.57 (11 to 26.13) | 38.45% (6.32% to 79.53%) | | 0.15 (0.1 to 0.29) | 0.03 (0.02 to 0.05) | -36.26%(-50% to -19.33%) |
| Southern Sub-Saharan Africa | 0.72 (0.65 to 0.78) | | 1.3 (1.18 to 1.4) | 31.1% (12.96% to 50.92%) | | 1.49 (1.31 to 1.66) | 2.99 (2.64 to 3.32) | -2.32% (-17.55% to 15.77%) | 7.41 (5.82 to 8.41) | | 15.96 (12.32 to 18.04) | 11.34% (-8.49% to 40.33%) | | 0.12 (0.1 to 0.13) | 0.18 (0.16 to 0.2) | -3.98%(-17.7% to 19.48%) |
| Eastern Sub-Saharan Africa | 2.17 (1.8 to 2.68) | | 1.27 (1.05 to 1.56) | 42.6% (13.04% to 80.07%) | | 3.63 (3.11 to 4.24) | 2.74 (2.35 to 3.19) | -8.46% (-29.91% to 12.11%) | 13.81 (10.91 to 16.67) | | 11.05 (8.69 to 13.22) | 6.52% (-5.77% to 20.22%) | | 0.32 (0.25 to 0.4) | 0.09 (0.07 to 0.11) | 23.53%(-8.26% to 62.22%) |
| Central Sub-Saharan Africa | 0.51 (0.38 to 0.69) | | 0.89 (0.66 to 1.25) | 14.53% (-17.05% to 54.56%) | | 1.38 (0.86 to 2.03) | 3.35 (2.06 to 4.93) | -23.13% (-40.11% to 5.85%) | 4.04 (2.81 to 5.17) | | 11.26 (7.77 to 14.45) | -9.86% (-25.92% to 9%) | | 0.13 (0.09 to 0.16) | 0.14 (0.1 to 0.18) | 17.23%(-11.05% to 55.7%) |
| South Asia | 10.86 (9.5 to 12.53) | | 0.78 (0.68 to 0.9) | 49.53% (18.95% to 102.23%) | | 21.99 (19.47 to 24.71) | 1.8 (1.59 to 2.01) | -10.72% (-24.89% to 9.34%) | 42.22 (35.17 to 54.87) | | 3.8 (3.13 to 4.97) | -12.76% (-28.22% to 13.12%) | | 2.68 (2.28 to 3.17) | 0.15 (0.13 to 0.17) | -3.32%(-23.3% to 24.4%) |
| North Africa and Middle East | 6 (5.16 to 6.89) | | 1.4 (1.21 to 1.61) | 48.87% (16.83% to 109.07%) | | 15.46 (13.17 to 18.42) | 4.05 (3.49 to 4.81) | -2.51% (-22.41% to 32.63%) | 19.09 (15.24 to 22.5) | | 5.83 (4.68 to 6.95) | 10.9% (-4.46% to 34.66%) | | 0.52 (0.44 to 0.62) | 0.09 (0.08 to 0.11) | 2.64%(-24% to 35.84%) |
| Tropical Latin America | 4.72 (4.41 to 4.99) | | 1.99 (1.85 to 2.1) | 32.42% (24.22% to 40.46%) | | 5.64 (5.12 to 6.03) | 2.45 (2.21 to 2.62) | -13.64% (-19.57% to -7.68%) | 23.9 (20.37 to 34.73) | | 10.55 (8.96 to 15.35) | -8.51% (-15.2% to -2.24%) | | 0.47 (0.44 to 0.52) | 0.2 (0.18 to 0.22) | 13.12%(-5.07% to 24.02%) |
| Central Latin America | 5.6 (4.8 to 6.51) | | 2.38 (2.04 to 2.76) | 30.32% (12.09% to 50.81%) | | 3.4 (2.93 to 3.95) | 1.51 (1.3 to 1.76) | -11.65% (-24.03% to 2.61%) | 21.67 (16.87 to 28.46) | | 9.83 (7.68 to 12.97) | 1.79% (-12.83% to 20.06%) | | 0.98 (0.8 to 1.2) | 0.38 (0.31 to 0.48) | 43.75%(14.24% to 70.02%) |
| Andean Latin America | 1.18 (0.94 to 1.46) | | 2.12 (1.69 to 2.63) | 17.96% (-7.95% to 48.87%) | | 0.87 (0.72 to 1.05) | 1.63 (1.33 to 1.97) | -8.37% (-25.84% to 12.67%) | 6.26 (4.85 to 7.86) | | 11.87 (9.18 to 14.92) | 5.13% (-18.66% to 32.77%) | | 0.21 (0.16 to 0.27) | 0.34 (0.26 to 0.44) | 21.33%(-23.53% to 86.55%) |
| Caribbean | 0.99 (0.83 to 1.16) | | 1.93 (1.62 to 2.27) | -10.11% (-24.23% to 4.92%) | | 1.52 (1.31 to 1.75) | 2.93 (2.54 to 3.38) | -1.5% (-14.76% to 13.48%) | 9.83 (7.85 to 12.06) | | 19.05 (15.23 to 23.38) | 12.89% (-7.58% to 31.27%) | | 0.05 (0.04 to 0.06) | 0.1 (0.08 to 0.13) | 149.2%(97.45% to 202%) |
| High-income North America | 22 (20.41 to 23.15) | | 3.49 (3.25 to 3.66) | -1.24% (-7.17% to 3.95%) | | 25.76 (23.43 to 27.45) | 3.81 (3.49 to 4.05) | 2.09% (-2.34% to 6.64%) | 54.85 (46.83 to 79.75) | | 8.02 (6.89 to 11.67) | -24.92% (-32.59% to 0.51%) | | 0.53 (0.5 to 0.59) | 0.12 (0.12 to 0.14) | -23.33%(-28.43% to -17.91%) |
| Southern Latin America | 4.19 (3.81 to 4.58) | | 5.05 (4.59 to 5.51) | -5.89% (-14.53% to 3.08%) | | 3.07 (2.81 to 3.3) | 3.58 (3.28 to 3.86) | -22.35% (-28.48% to -15.91%) | 10.1 (8.38 to 13.38) | | 11.65 (9.67 to 15.42) | 1.09% (-7.78% to 10.55%) | | 0.42 (0.37 to 0.46) | 0.58 (0.52 to 0.65) | -6.11%(-15.94% to 6.4%) |
| Western Europe | 34.36 (31.51 to 36.3) | | 3.65 (3.4 to 3.83) | 4.08% (-0.93% to 8.83%) | | 50.51 (45.16 to 54.46) | 4.78 (4.32 to 5.14) | -21.62% (-26.09% to -17.09%) | 95.77 (79.23 to 133.05) | | 8.73 (7.25 to 12.1) | -13.5% (-20.04% to 6.36%) | | 0.82(0.7 to 0.87) | 0.14 (0.13 to 0.15) | -38.75%(-43.9% to -34%) |
| Australasia | 1.71 (1.54 to 1.89) | | 3.41 (3.08 to 3.75) | -6.72% (-14.62% to 2.53%) | | 1.63 (1.43 to 1.81) | 2.96 (2.61 to 3.29) | -24.56% (-31.37% to -17.12%) | 5.41 (4.43 to 7.64) | | 9.77 (8.1 to 13.93) | -14.5% (-24.55% to 8.74%) | | 0.04 (0.03-0.04) | 0.12 (0.1 to 0.13) | -35.67%(-43.6% to -33.8%) |
| High-income Asia Pacific | 9.71 (8.42 to 10.48) | | 2 (1.79 to 2.13) | 18.24% (9.49% to 24.63%) | | 12.88 (10.68 to 14.23) | 2.26 (1.92 to 2.47) | -18.57% (-26.17% to -13.3%) | 19.38 (14.97 to 23.66) | | 3.39 (2.7 to 4.24) | -5.36% (-23.02% to 0.36%) | | 0.13 (0.12 to 0.14) | 0.05 (0.05 to 0.06) | -41.12%(-46.8% to -33.8%) |
| Eastern Europe | 14.9 (13.4 to 16.55) | | 4.36 (3.92 to 4.84) | 31.79% (15.28% to 49.85%) | | 10.93 (9.7 to 12.2) | 3.11 (2.75 to 3.47) | -0.07% (-10.62% to 12.37%) | 20.46 (15.2 to 25.07) | | 5.74 (4.26 to 7.02) | 49.83% (8.76% to 79.28%) | | 0.55 (0.47 to 0.63) | 0.22 (0.19 to 0.26) | -4.07%(-17.16% to 11.73%) |
| Central Europe | 9.63 (8.5 to 10.84) | | 4.52 (3.99 to 5.11) | 83.17% (60.8% to 105.76%) | | 11.88 (10.47 to 13.44) | 5.29 (4.66 to 5.99) | 11.56% (-1.15% to 25.39%) | 18.83 (14.1 to 22.05) | | 8.15 (6.11 to 9.56) | 19.2% (-8.53% to 40.18%) | | 0.5 (0.43 to 0.58) | 0.37 (0.31 to 0.42) | -17.34%（-29.77% to -3.82%） |
| Central Asia | 2.06 (1.87 to 2.29) | | 2.72 (2.48 to 3) | 45.72% (20.58% to 76.72%) | | 1.6 (1.44 to 1.77) | 2.56 (2.3 to 2.82) | 17.9% (1.82% to 42.66%) | 2.57 (2.06 to 3.06) | | 4.56 (3.59 to 5.42) | 34.82% (15.73% to 55.99%) | | 0.2 (0.18 to 0.24) | 0.25 (0.22 to 0.3) | 8.91%(-7.2% to 29%) |
| Oceania | 0.05 (0.04 to 0.06) | | 0.64 (0.5 to 0.85) | 12.67% (-7.27% to 36.69%) | | 0.1 (0.08 to 0.13) | 1.82 (1.44 to 2.27) | 19.12% (-1.07% to 42.83%) | 0.51 (0.38 to 0.65) | | 11.39 (8.52 to 14.39) | 18.22% (-4.61% to 46.16%) | | 0.02(0.01to 0.02) | 0.26 (0.21 to 0.32) | -17.08%(-39% to 11.79%) |
| Southeast Asia | 7.53 (6.15 to 9.48) | | 1.27 (1.04 to 1.6) | 52.72% (24.08% to 90.59%) | | 9.03 (7.88 to 10.5) | 1.76 (1.53 to 2.03) | -3.87% (-17.91% to 13.51%) | 27.21 (20.47 to 32.07) | | 5.59 (4.24 to 6.57) | 18.75% (-0.64% to 42.4%) | | 0.76 (0.64 to 0.9) | 0.11 (0.1 to 0.14) | 28.73%(5.71% to 56.41%) |
| East Asia | 25.49 (21.43 to 29.95) | | 1.3 (1.1 to 1.52) | 85.13% (47.58% to 131.41%) | | 42.16 (36.02 to 49.35) | 2.27 (1.95 to 2.64) | -12.33% (-27.88% to 7.3%) | 57.21 (45.38 to 74.04) | | 3.11 (2.49 to 3.98) | -1.7% (-21.87% to 24.12%) | | 1.25 (1.01 to 1.52) | 0.07 (0.06 to 0.09) | -2.69%(-25.15 to 25.6%) |
