# Supplementary material for: Trends and risk factors of global incidence, mortality, and disability of genitourinary cancers from 1990 to 2019: Systematic analysis for the Global Burden of Disease Study 2019
Source: Front Public Health. 2023 Feb 22;11:1119374. doi: 10.3389/fpubh.2023.1119374 (PMC9992434; doi:10.3389/fpubh.2023.1119374)
Supplement: Supplementary file 14 [file Table_3.DOCX]

| Table 3S. Number of disability-adjusted life-year (DALY), age standardized DALY rate in 2019 and the percentage change of DALY rate for bladder, kidney， prostate and testicular cancer between 1990 and 2019 | | | | | | | | | | | | |
| --- | --- | --- | --- | --- | --- | --- | --- | --- | --- | --- | --- | --- |
|  | **Kidney cancer** | | | **Bladder cancer** | | | **Prostate cancer** | | | **Testicular cancer** | | |
| location | DALY number (×1000) in 2019 | Age standardized DALY rate per 100,000 in 2019 | Change of age standardized DALY rate between 1990 and 2019 | DALY number (×1000) in 2019 | Age standardized DALY rate per 100,000 in 2019 | Change of age standardized DALY rate between 1990 and 2019 | DALY number (×1000) in 2019 | Age standardized DALY rate per 100,000 in 2019 | Change of age standardized DALY rate between 1990 and 2019 | DALY number (×1000) in 2019 | Age standardized DALY rate per 100,000 in 2019 | Change of age standardized DALY rate between 1990 and 2019 |
| Global |  |  |  |  |  |  |  |  |  |  |  |  |
| Both sexes | 4052.82 (3801.04 to 4317.49) | 49.62 (46.46 to 52.94) | 4.94% (-2.28% to 13.61%) | 4392.58 (4090.44 to 4702.73) | 54.2 (50.35 to 57.97) | -18.61% (-24.29% to -11.2%) | 8644.87 (7548.02 to 10559.87) | 107.89 (94.03 to 131.7) | -9.33% (-15.44% to 1.16%) | 562.19(627.98 to 510.77) | 7.13(8.02 to 6.46) | -2.83%  (7.83% to -11.7%) |
| Females | 1317.11 (1228.35 to 1413.53) | 31.08 (28.95 to 33.38) | -9.39% (-15.58% to -3.53%) | 1066.08 (962.71 to 1150.12) | 24.43 (22.1 to 26.35) | -22.85% (-30.55% to -14.14%) | / | / | / | / | / | / |
| Males | 2735.71 (2544.82 to 2931.33) | 70.06 (65.14 to 75.07) | 13.08% (2.74% to 24.94%) | 3326.5 (3093.91 to 3570.17) | 90.17 (83.63 to 96.61) | -19.26% (-25.26% to -11.41%) | 8644.87 (7548.02 to 10559.87) | 244.07 (211.78 to 297.72) | -14.75% (-20.33% to -5.26%) | 562.19(627.98 to 510.77) | 7.13(8.02 to 6.46) | -2.83%  (7.83% to -11.7%) |
| China | 642.8 (533.66 to 763.98) | 34.28 (28.95 to 40.16) | 58.82% (26.07% to 95.65%) | 816.12 (691.35 to 967.38) | 41.88 (35.59 to 49.34) | -15.25% (-31.2% to 5.65%) | 1002.59 (794.01 to 1322.63) | 52.08 (41.49 to 68.25) | -2.9% (-24.71% to 27.85%) | 51.15(61.19 to 42.02) | 3.38(2.78 to 3.99) | 3.93%(33.03% to -19.48%) |
| Democratic People's Republic of Korea | 8.85 (6.05 to 11.7) | 28.5 (19.8 to 37.44) | 6.42% (-30.45% to 48%) | 12.52 (10.31 to 15.17) | 38.9 (32.35 to 46.83) | -10.99% (-33.68% to 23.51%) | 17.09 (13.01 to 20.75) | 54.16 (41.37 to 65.62) | 11.18% (-15.23% to 49.14%) | 0.97(1.41 to 0.65) | 3.44(2.3 to 4.91) | 4.97%(61.25% to -31.37%) |
| Taiwan (Province of China) | 27.91 (21.58 to 36.45) | 75.44 (58.84 to 97.7) | 193.37% (127.36% to 276.76%) | 28.33 (21.75 to 36.87) | 71.73 (55.08 to 93.47) | -3.63% (-25.33% to 26%) | 33.54 (22.37 to 44.2) | 84.17 (55.99 to 110.09) | 21.82% (-14.88% to 60.49%) | 1.37(1.8 to 1) | 5.7(4.19 to 7.61) | 48.2%(99.55% to 8.24%) |
| Cambodia | 4.14 (3.2 to 5.15) | 30.64 (23.54 to 37.96) | 38.76% (-8.35% to 106.65%) | 4.42 (3.34 to 5.52) | 38.36 (29.09 to 47.68) | -6.11% (-30.23% to 22.32%) | 12.37 (8.74 to 15.4) | 119.93 (85.14 to 148.19) | 21.86% (-5.76% to 55.47%) | 0.79(1.05 to 0.58) | 4.72(3.58 to 6.19) | 40.1%(117.49% to -3.92%) |
| Indonesia | 88.81 (63.29 to 128.19) | 37.6 (27.06 to 54.17) | 75.17% (29.99% to 125.8%) | 70.99 (53.04 to 101.43) | 34.6 (26.12 to 48.95) | 9.93% (-16.62% to 39.27%) | 215.9 (149.9 to 270.17) | 120.06 (84.24 to 149.79) | 55.05% (18.05% to 102.43%) | 12.64(17.04 to 9.7) | 4.79(3.69 to 6.39) | 41.39%(97.82% to 5.76%) |
| Lao People's Democratic Republic | 1.67 (1.21 to 2.33) | 31.1 (23.01 to 42.67) | 20.47% (-23.09% to 95.67%) | 1.54 (1.1 to 1.98) | 36.35 (26.43 to 45.45) | -21.98% (-42.57% to 6.84%) | 3.93 (2.81 to 4.94) | 108.12 (77.64 to 134.72) | 13.87% (-12.44% to 46.71%) | 0.31(0.41 to 0.21) | 4.39(3.01 to 5.73) | 19.77%(100.67% to -25.73%) |
| Malaysia | 10.99 (8.05 to 14.21) | 39.12 (28.89 to 50.43) | 58.81% (11.74% to 127.95%) | 13.94 (10.8 to 17.61) | 55.42 (43.41 to 69.45) | -2.01% (-27.85% to 39.04%) | 21.07 (14.54 to 27.75) | 89.01 (61.43 to 117.87) | 16.15% (-11.5% to 58.2%) | 2.46(3.56 to 1.62) | 7.21(4.85 to 10.32) | 18.92%(89.29% to -31.09%) |
| Maldives | 0.09 (0.08 to 0.11) | 26.59 (21.69 to 31.8) | 20.75% (-20.7% to 86.94%) | 0.09 (0.07 to 0.11) | 33.31 (26.73 to 40.1) | -30.67% (-50.76% to -4.64%) | 0.17 (0.13 to 0.23) | 67.18 (51.99 to 88.79) | -12.73% (-35.63% to 15.11%) | 0(0.01 to 0) | 0.85(0.62 to 1.15) | 20.83%(100.46% to -26.99%) |
| Myanmar | 16.61 (12.45 to 22.95) | 33.05 (25.12 to 45.27) | 39.43% (-13.05% to 132.26%) | 15.6 (12.53 to 19.49) | 34.51 (27.96 to 42.58) | -22.7% (-42.15% to 7.16%) | 40.08 (28.68 to 50.85) | 98.09 (70.62 to 123.58) | 10.35% (-13.47% to 44.36%) | 2.42(3.44 to 1.67) | 4.39(3.03 to 6.26) | 18.58%(95.99% to -24.32%) |
| Philippines | 36.94 (30.44 to 45.08) | 38.95 (31.94 to 47.71) | 22.38% (-0.93% to 48.15%) | 18.5 (15.17 to 22.34) | 23.47 (19.45 to 28.08) | -13.81% (-32.36% to 10.11%) | 83.15 (55.8 to 111.34) | 121.39 (81.5 to 160.83) | -10.31% (-35.4% to 31.18%) | 7.17(9.3 to 5.57) | 6.57(5.11 to 8.51) | -6.87%(22.03% to -27.88%) |
| Sri Lanka | 16.88 (11.97 to 23.1) | 64.74 (46.54 to 87.95) | -6.73% (-36.92% to 34.52%) | 6.28 (4.66 to 8.36) | 25.12 (18.78 to 32.89) | 16.72% (-14.85% to 57.42%) | 12.36 (8.91 to 16.86) | 50.36 (36.76 to 67.84) | -21.08% (-42.6% to 7.27%) | 0.8(1.18 to 0.52) | 3.68(2.37 to 5.45) | 180.42%(368.1% to 68.9%) |
| Thailand | 23.14 (16.78 to 30.33) | 24.3 (18.08 to 31.67) | 16.74% (-14.7% to 57.45%) | 30.18 (22.57 to 39.29) | 30.05 (22.57 to 39.04) | -45.22% (-60.43% to -26.03%) | 62.55 (43.75 to 85.56) | 62.64 (43.94 to 85.27) | -12.12% (-36.73% to 22.09%) | 3.51(4.96 to 2.33) | 5.21(3.54 to 7.25) | 13.48%(74.36% to -27.12%) |
| Timor-Leste | 0.26 (0.17 to 0.39) | 26.38 (17.27 to 39.17) | 49.37% (-9.77% to 142.74%) | 0.24 (0.17 to 0.33) | 30.7 (22.11 to 41.51) | 1.57% (-27.06% to 50.53%) | 0.85 (0.55 to 1.12) | 118.4 (78.54 to 154.63) | 51.61% (6.61% to 123.17%) | 0.05(0.08 to 0.02) | 4.34(1.98 to 6.26) | 39.09%(135.43% to -39.52%) |
| Viet Nam | 24.73 (17.84 to 34.24) | 25.02 (18.29 to 34.42) | 67.21% (16.37% to 129.28%) | 31.99 (24.83 to 39.95) | 35.17 (27.6 to 43.28) | 19.48% (-10.46% to 62.52%) | 54.79 (41.44 to 78.42) | 66.14 (50.66 to 94.14) | 22.02% (-10.43% to 68.91%) | 6.84(10.51 to 4.33) | 6.48(4.14 to 9.86) | 49.33%(165.75% to -15.7%) |
| Fiji | 0.19 (0.14 to 0.24) | 22.67 (17.32 to 28.93) | 24.9% (-12.39% to 75.77%) | 0.34 (0.26 to 0.43) | 44.46 (35.16 to 55.39) | 22.19% (-11.04% to 69.49%) | 1 (0.44 to 1.46) | 160.54 (73.12 to 229.83) | -2.7% (-32.54% to 40.97%) | 0.15(0.22 to 0.1) | 17.76(12.28 to 24.93) | 18.49%(95.37% to -27.65%) |
| Kiribati | 0.07 (0.05 to 0.1) | 75.58 (54.3 to 105.43) | 14.31% (-20.84% to 70.14%) | 0.02 (0.02 to 0.03) | 34.91 (27.98 to 42.32) | -6.47% (-27.78% to 19.16%) | 0.06 (0.04 to 0.07) | 101.89 (75.9 to 133.36) | -19.67% (-38.18% to 3.42%) | 0.09(0.13 to 0.06) | 75.25(49.99 to 107.29) | 10.8%(86.08% to -34.35%) |
| Marshall Islands | 0.01 (0.01 to 0.02) | 25.3 (17.86 to 34.82) | 26.21% (-9.97% to 79.62%) | 0.02 (0.02 to 0.03) | 57.49 (43.95 to 74.33) | 13.39% (-13.98% to 50.12%) | 0.06 (0.04 to 0.08) | 248.74 (185.9 to 321.33) | 6.35% (-25.89% to 57.52%) | 0(0 to 0) | 3.4(2.32 to 4.74) | 15.26%(63.92% to -19.15%) |
| Micronesia (Federated States of) | 0.03 (0.01 to 0.04) | 30.23 (17.07 to 41.86) | 15.59% (-30.77% to 68.11%) | 0.04 (0.03 to 0.06) | 57.52 (42.37 to 75.28) | 15.82% (-18.69% to 60.61%) | 0.12 (0.08 to 0.17) | 219.91 (153.53 to 290.49) | 4.6% (-32.62% to 57.69%) | 0(0.01 to 0) | 3.36(1.9 to 5.28) | 9.35%(56.77% to -33.74%) |
| Papua New Guinea | 1.14 (0.79 to 1.63) | 14.81 (10.48 to 21.64) | 21.45% (-11.45% to 63.57%) | 1.73 (1.22 to 2.45) | 35.11 (24.84 to 48.32) | 24.79% (-5.92% to 62.89%) | 6.56 (4.46 to 9.09) | 185.48 (125.67 to 254.66) | 32.82% (-2.52% to 83.93%) | 0.26(0.37 to 0.18) | 2.71(1.9 to 3.86) | 11.28%(56.36% to -18.4%) |
| Samoa | 0.04 (0.03 to 0.05) | 23.56 (17.65 to 31.1) | -4.15% (-32.01% to 37.4%) | 0.06 (0.05 to 0.08) | 44.53 (35.86 to 55.97) | -1.98% (-24.86% to 29.69%) | 0.15 (0.11 to 0.18) | 111.64 (84.94 to 134.93) | -16.7% (-30.81% to 1.4%) | 0.03(0.05 to 0.02) | 14.71(7.54 to 24.89) | -6.43%(88.94% to -59.94%) |
| Solomon Islands | 0.11 (0.08 to 0.16) | 24.6 (17 to 34.91) | 24.88% (-9.84% to 74.32%) | 0.21 (0.14 to 0.29) | 59.78 (43.04 to 80.85) | 20% (-11.3% to 61.9%) | 0.53 (0.39 to 0.68) | 230.26 (173.75 to 288.06) | 2.74% (-27.45% to 54.54%) | 0.02(0.03 to 0.01) | 3.27(2.09 to 4.67) | 20.23%(64.32% to -13.49%) |
| Tonga | 0.02 (0.01 to 0.03) | 24.05 (16.1 to 33.48) | 36.87% (-1.92% to 85.76%) | 0.03 (0.02 to 0.04) | 36.81 (27.28 to 48.74) | 21.33% (-7.13% to 59.15%) | 0.18 (0.14 to 0.22) | 234.56 (178.16 to 286.48) | 1.87% (-19.15% to 30.2%) | 0.17(0.25 to 0.11) | 190.62(129.31 to 278.55) | 12.05%(81.85% to -32.61%) |
| Vanuatu | 0.05 (0.03 to 0.07) | 21.56 (14.32 to 30.94) | 29.33% (-11.07% to 84.57%) | 0.08 (0.06 to 0.11) | 44.73 (32.71 to 59.52) | 35.42% (-1.88% to 86.3%) | 0.33 (0.24 to 0.44) | 227.22 (167.05 to 300.5) | 10.02% (-22.68% to 62.35%) | 0.01(0.01 to 0) | 2.79(1.74 to 3.93) | 23.44%(82.2% to -16.72%) |
| Armenia | 2.58 (2.15 to 3.08) | 65.31 (54.84 to 77.39) | 223.75% (161.54% to 300.8%) | 4.35 (3.58 to 5.21) | 104.25 (85.78 to 124.24) | -5.99% (-26.64% to 19.6%) | 5.33 (3.17 to 6.65) | 127.71 (75.84 to 158.7) | 60.74% (4.85% to 103.52%) | 0.36(0.46 to 0.28) | 11.82(9.21 to 16.03) | 0.16%(40.67% to -38.98%) |
| Azerbaijan | 11.22 (8.57 to 14.94) | 101.94 (78.65 to 134.88) | 8.69% (-16.78% to 40.54%) | 5.74 (4.66 to 7.09) | 58.62 (48.23 to 71.35) | 8.17% (-15.44% to 37.85%) | 8.83 (6.84 to 11.75) | 107.66 (83.52 to 143.4) | 29.25% (0.55% to 65.31%) | 0.82(1.13 to 0.56) | 7.24(5 to 9.96) | 15.62%(90.92% to -32.75%) |
| Georgia | 4.54 (3.71 to 5.41) | 85.27 (70.23 to 101.33) | 45.81% (8.89% to 93.11%) | 5.87 (4.9 to 6.93) | 100.42 (83.43 to 118.77) | 43.12% (14.37% to 78.09%) | 8.91 (5.17 to 11) | 147.75 (84.56 to 182.55) | 63.91% (9.74% to 111.17%) | 1.31(1.65 to 1.05) | 32.53(25.84 to 41.53) | 54.94%(120.23% to -9.04%) |
| Kazakhstan | 16.87 (14.39 to 19.71) | 90.06 (77.18 to 104.79) | 8.6% (-18.99% to 43.9%) | 8.72 (7.37 to 10.36) | 49.94 (42.1 to 59.12) | -7.19% (-28.81% to 21.02%) | 13.08 (10.18 to 17.39) | 78.9 (61.78 to 105.2) | 15.04% (-3.53% to 37.75%) | 1.89(2.9 to 1.51) | 10.2(8.19 to 15.24) | 5.46%(43.65% to -18.13%) |
| Kyrgyzstan | 2.84 (2.4 to 3.37) | 53.04 (45.04 to 62.61) | 67.87% (29.94% to 113.1%) | 1.46 (1.23 to 1.73) | 31.67 (26.57 to 37.14) | -19.15% (-33.53% to -0.61%) | 2.18 (1.62 to 2.73) | 53.62 (39.09 to 66.3) | -17.93% (-34.9% to 36.96%) | 0.48(0.58 to 0.39) | 7.59(6.12 to 9.11) | -8.32%(21.45% to -37.45%) |
| Mongolia | 1.68 (1.29 to 2.21) | 63.28 (49.28 to 82.34) | 110.63% (52.25% to 188.63%) | 0.64 (0.49 to 0.82) | 27.47 (21.46 to 34.4) | -45.4% (-58.98% to -26.85%) | 1.14 (0.87 to 1.57) | 57.19 (44.42 to 78.15) | 1.54% (-24.06% to 36.82%) | 0.06(0.08 to 0.03) | 1.69(1.01 to 2.46) | 228.84%(416.6% to 84%) |
| Tajikistan | 4.07 (3.25 to 5.06) | 61.65 (49.1 to 76.01) | 17.99% (-25.83% to 63.46%) | 1.55 (1.25 to 1.93) | 32.92 (27.08 to 40.12) | 21.34% (-8.91% to 73.17%) | 2.81 (2.09 to 4.25) | 75.92 (51.35 to 112.79) | 27.56% (-4.05% to 83.65%) | 0.04(0.05 to 0.03) | 0.4(0.32 to 0.5) | 17.26%(52.77% to -10.3%) |
| Turkmenistan | 4.95 (3.98 to 6.31) | 105.97 (85.18 to 134.11) | 126.85% (70.45% to 203.01%) | 1.33 (1.04 to 1.72) | 32.24 (24.84 to 41.2) | 59.07% (22.65% to 104.41%) | 2.39 (1.79 to 3.99) | 64.38 (47.89 to 112.79) | 14.64% (-8.98% to 45.62%) | 0.88(1.47 to 0.62) | 17.47(12.42 to 28.66) | 5.64%(48.73% to -23.05%) |
| Uzbekistan | 15.11 (12.46 to 18) | 54.74 (45.57 to 64.65) | 99.83% (44.32% to 193.54%) | 9.53 (7.78 to 11.49) | 44.38 (36.8 to 52.76) | 50.88% (6.32% to 113.85%) | 9.11 (7.06 to 12.81) | 63.56 (42.74 to 78.42) | 47.95% (17.73% to 124.66%) | 3.05(3.83 to 2.42) | 9.11(7.28 to 11.36) | 15.54%(50.52% to -9.69%) |
| Albania | 2.26 (1.66 to 2.94) | 57.83 (43.26 to 74.44) | 52.33% (12.81% to 98.64%) | 0.88 (0.66 to 1.15) | 20.7 (15.62 to 27.07) | -12.47% (-34.9% to 14.35%) | 6.64 (4.1 to 11.75) | 150.23 (94.11 to 262.47) | 4.68% (-22.32% to 39.33%) | 0.51(0.73 to 0.35) | 18.68(13 to 26.7) | 25.01%(88.79% to -15.46%) |
| Bosnia and Herzegovina | 5.08 (3.89 to 6.41) | 90.92 (70.56 to 115.02) | 71.28% (30.99% to 120.83%) | 5.77 (4.49 to 7.22) | 94.53 (73.83 to 118.02) | 38.77% (7.45% to 75%) | 8.04 (5.52 to 10.55) | 130.68 (90.14 to 170.86) | 43% (0.23% to 93.31%) | 0.52(0.68 to 0.39) | 15.33(11.41 to 20.18) | 99.08%(176.5% to 40.92%) |
| Bulgaria | 10.28 (7.96 to 13.04) | 86.24 (67.13 to 109.94) | 158.96% (98.89% to 234.07%) | 14.81 (11.51 to 18.5) | 105.05 (81.28 to 131.23) | 34.8% (4.6% to 69.63%) | 23.33 (16.39 to 29.89) | 150.44 (104.91 to 192.32) | 32.66% (-14.66% to 77.18%) | 1.84(2.4 to 1.41) | 26.6(20.09 to 34.93) | -19.72%(6.14% to -40.15%) |
| Croatia | 8.24 (6.3 to 10.54) | 103.57 (78.96 to 133.17) | 161.43% (96.96% to 246.24%) | 8.45 (6.64 to 10.53) | 94.35 (74.16 to 118.44) | -0.25% (-22.13% to 27.97%) | 15.35 (10.75 to 19.79) | 162.24 (113.37 to 209.24) | 9.08% (-14.91% to 38.46%) | 0.76(1 to 0.57) | 18.3(13.68 to 24.46) | 14.85%(59.1% to -18.44%) |
| Czechia | 28.55 (22.99 to 35.75) | 144.85 (116.62 to 181.41) | 10.18% (-11.68% to 38.15%) | 18.22 (14.54 to 22.19) | 85.22 (67.59 to 104.17) | -22.25% (-38.87% to -3.75%) | 33.7 (24.9 to 43.8) | 151.01 (111.86 to 196.77) | 7.22% (-22.11% to 31.69%) | 1.94(2.51 to 1.49) | 18.26(13.89 to 24.67) | -34.34%(-8.01% to -50.52%) |
| Hungary | 20.31 (16.53 to 25.12) | 117.51 (94.88 to 145.3) | -5.14% (-23.69% to 18.4%) | 20.7 (16.87 to 25.17) | 108.74 (88.96 to 132.44) | -1.25% (-20.4% to 19.55%) | 27.75 (21.09 to 36.17) | 135.91 (103.01 to 176.71) | -9.59% (-26.31% to 9.17%) | 2.32(2.9 to 1.81) | 24.42(18.88 to 31.09) | -26.7%(-4.07% to -45.76%) |
| North Macedonia | 1.77 (1.37 to 2.25) | 57.5 (44.93 to 72.78) | 144.22% (87.52% to 213.8%) | 3.86 (3.03 to 4.91) | 118.36 (93.87 to 149.42) | 18.8% (-8.16% to 51.64%) | 5.4 (4.14 to 7.18) | 169.91 (131.01 to 223.81) | 36.64% (-13.03% to 107.05%) | 0.57(0.81 to 0.39) | 24.21(16.55 to 34.23) | -12.89%(34.67% to -43.43%) |
| Montenegro | 0.77 (0.63 to 0.92) | 81.22 (66.66 to 97.57) | 27.32% (0.68% to 62%) | 0.9 (0.74 to 1.1) | 90.38 (74.2 to 109.55) | 6.69% (-18.23% to 42.12%) | 1.88 (1.46 to 2.36) | 185.8 (144.82 to 232.87) | 24.23% (-4.32% to 63.48%) | 0.13(0.19 to 0.09) | 21.97(14.91 to 32.37) | -2.53%(66.37% to -40.93%) |
| Poland | 82.17 (68.51 to 97.65) | 125.84 (104.54 to 149.65) | 157.32% (113.59% to 205.32%) | 89.63 (74.51 to 108.32) | 127.55 (105.8 to 154.36) | 9.75% (-9.27% to 32.82%) | 112.7 (70.84 to 142.25) | 154.04 (97.02 to 194.52) | 26.89% (-16.31% to 64.21%) | 8.8(10.8 to 7.07) | 22.32(17.94 to 27.17) | -10.25%(12.42% to -28.82%) |
| Romania | 27.43 (22.27 to 33.71) | 88.13 (71.9 to 107.55) | 55.36% (24.82% to 89.71%) | 37.28 (30.29 to 46.14) | 102.51 (83.38 to 127.33) | 20.12% (-2.09% to 48.76%) | 47.18 (32.02 to 58.13) | 120.64 (80.91 to 149.12) | 32.37% (-8.94% to 67.52%) | 3.49(4.34 to 2.76) | 18.51(14.52 to 23.55) | -5.18%(22.46% to -29.54%) |
| Serbia | 14.11 (10.94 to 17.88) | 96.3 (74.66 to 122.79) | 33.42% (-0.21% to 77.01%) | 18.79 (14.81 to 23.53) | 116.72 (92.18 to 146.27) | 12.13% (-14.91% to 48.29%) | 26.25 (18.73 to 33.46) | 154.52 (111.81 to 196.7) | 28.36% (-4.36% to 73.61%) | 1.92(2.59 to 1.42) | 21.88(16.02 to 29.78) | 2.5%(44.2% to -29.59%) |
| Slovakia | 10.89 (8.32 to 13.98) | 123.08 (93.94 to 157.28) | 55.29% (18.13% to 98.84%) | 7.88 (6.27 to 9.98) | 84.31 (66.7 to 106.44) | -11.84% (-30.61% to 12.18%) | 13.05 (8.64 to 17.46) | 136.77 (90.61 to 182.44) | 10.97% (-22.13% to 50.21%) | 1.28(1.88 to 0.81) | 22.67(14.18 to 33.16) | 11.31%(71.92% to -33.21%) |
| Slovenia | 3.6 (2.73 to 4.74) | 91.89 (69.64 to 120.93) | 49.69% (3.27% to 116.82%) | 3.36 (2.56 to 4.29) | 76.3 (57.8 to 98.6) | -11.66% (-38.74% to 25.64%) | 8.54 (5.56 to 11.31) | 186.7 (123.82 to 248.35) | 37.42% (-10.58% to 96.03%) | 0.28(0.37 to 0.21) | 13.68(9.99 to 18.72) | -9.39%(28.74% to -36.75%) |
| Belarus | 18.1 (13.63 to 23.51) | 120.65 (91.39 to 157.5) | 215.73% (134.37% to 320.31%) | 8.66 (6.68 to 11.24) | 54.14 (41.84 to 70.57) | -20.97% (-38.77% to 3.35%) | 22.96 (17.05 to 34.26) | 142.1 (105.47 to 210.55) | 47.54% (5.08% to 99.79%) | 1.15(1.56 to 0.78) | 11.83(8.14 to 16.07) | 74.3%(149.79% to 15.81%) |
| Estonia | 3.07 (2.35 to 3.95) | 128.5 (98.03 to 166.15) | 141.42% (82.12% to 212.21%) | 1.98 (1.53 to 2.5) | 73.68 (56.73 to 93.54) | -7.31% (-28.77% to 20.54%) | 5.22 (2.74 to 6.88) | 189.05 (101.58 to 249.89) | 52.09% (2.25% to 101.01%) | 0.11(0.15 to 0.08) | 8.51(6.27 to 11.86) | -29.17%(0.57% to -49.35%) |
| Latvia | 4.41 (3.55 to 5.41) | 125.34 (100.61 to 154.87) | 167.01% (115.3% to 235.2%) | 3.73 (2.95 to 4.66) | 94.09 (74.4 to 117.73) | 14.57% (-9.96% to 45.03%) | 7.26 (4.18 to 9.42) | 174.79 (102.13 to 228.6) | 49.86% (1.79% to 97.18%) | 0.27(0.35 to 0.2) | 13.69(10.01 to 18.24) | -0.41%(33.87% to -28.65%) |
| Lithuania | 6.76 (5.45 to 8.29) | 134.17 (108.22 to 164.1) | 144.73% (95.6% to 205.33%) | 4.59 (3.62 to 5.71) | 79.54 (62.21 to 99.7) | -10.41% (-30.11% to 12.43%) | 10.51 (5.86 to 13.29) | 176.98 (100.57 to 226.17) | 50.68% (-7.83% to 97.1%) | 0.29(0.37 to 0.22) | 10.61(8.13 to 13.68) | 41.37%(83.46% to 2.92%) |
| Republic of Moldova | 4.36 (3.73 to 5.1) | 83.54 (72.55 to 96.76) | 41.28% (21.04% to 63.25%) | 3.92 (3.29 to 4.72) | 67.43 (56.8 to 80.95) | -6.35% (-21.63% to 10.15%) | 6.56 (4.52 to 7.98) | 110.9 (76.24 to 135.05) | 35.34% (-0.43% to 66.14%) | 0.44(0.53 to 0.36) | 11.22(9.26 to 14.21) | 12.04%(44.55% to -22.34%) |
| Russian Federation | 253.08 (218.25 to 291.65) | 112.81 (97.33 to 129.96) | 6.74% (-6.73% to 21.69%) | 150.97 (127.55 to 175.54) | 64.12 (54.21 to 74.5) | -9.64% (-22.63% to 4.21%) | 272.5 (175.14 to 335.13) | 112.49 (72.84 to 138.64) | 70.57% (9.66% to 115.88%) | 16.75(20.29 to 13.91) | 10.98(9.13 to 13.48) | -13.35%(4.29% to -26.95%) |
| Ukraine | 87.15 (71.9 to 105.2) | 128.35 (106.82 to 154.54) | 41.6% (3.93% to 96.08%) | 57.68 (47.05 to 69.37) | 76.6 (62.64 to 92.39) | 23.74% (-9.32% to 64.04%) | 104.01 (80.15 to 151.17) | 133.57 (102.83 to 191.4) | 27.85% (1.2% to 59.4%) | 8.1(10.34 to 6.21) | 17.19(13.19 to 21.94) | 82.03%(150.24% to 36.22%) |
| Brunei Darussalam | 0.3 (0.26 to 0.35) | 91.49 (77.72 to 105.7) | 30.23% (-4.17% to 64.2%) | 0.16 (0.14 to 0.19) | 67.26 (57.87 to 78.57) | -21.77% (-42.52% to 0.82%) | 0.27 (0.2 to 0.33) | 125.12 (85.79 to 158.4) | 12.75% (-18.36% to 52.31%) | 0.05(0.07 to 0.03) | 9.73(6.75 to 14.06) | -14.97%(42.95% to -48.86%) |
| Japan | 129.82 (116.75 to 138.1) | 43.46 (40.38 to 45.94) | 1.64% (-3.51% to 6.08%) | 148.85 (128.23 to 164.04) | 39.35 (34.9 to 43.1) | -17.96% (-24.22% to -12.77%) | 245.08 (191.46 to 321.32) | 60.6 (48.83 to 82.42) | -1.38% (-14.15% to 5.64%) | 5.22(6.15 to 4.5) | 4.6(3.81 to 5.77) | -36.31%(-20.45% to -46.05%) |
| Republic of Korea | 34.64 (30.65 to 39.07) | 40.85 (36.3 to 45.81) | 54.93% (34.49% to 78.96%) | 34.46 (30.43 to 38.81) | 38.91 (34.32 to 43.73) | -26.59% (-35.23% to -16.18%) | 48.81 (38.72 to 62.26) | 54.6 (43.28 to 69.17) | 10.84% (-31.45% to 29.16%) | 0.87(1.13 to 0.64) | 1.74(1.19 to 2.38) | -6.05%(35.56% to -39.07%) |
| Singapore | 3.58 (3.2 to 4.05) | 45.93 (41.23 to 51.87) | -3.43% (-15.93% to 11.05%) | 1.99 (1.69 to 2.32) | 26.19 (22.31 to 30.7) | -48.05% (-56.43% to -38.95%) | 4.58 (3.52 to 5.99) | 61.43 (46.93 to 79.92) | -6.09% (-33.22% to 9.12%) | 0.11(0.16 to 0.09) | 1.92(1.43 to 2.98) | -57.92%(-34.24% to -69.19%) |
| Australia | 29.41 (26.33 to 32.49) | 77.27 (69.78 to 85.09) | -11.92% (-20.42% to -2.21%) | 21.68 (19.14 to 24.47) | 50.46 (44.77 to 56.67) | -31.78% (-38.6% to -24.09%) | 78.47 (64.11 to 112.13) | 178.6 (145.71 to 255.1) | -12.52% (-25.01% to 12.69%) | 1.76(2.33 to 1.39) | 7.31(5.49 to 10.15) | -23.22%(0% to -39.63%) |
| New Zealand | 5.74 (5.16 to 6.32) | 81.11 (73.69 to 88.83) | -5.46% (-16.71% to 6.67%) | 4.17 (3.69 to 4.65) | 52.6 (46.7 to 58.41) | -30.44% (-38.16% to -22.18%) | 15.19 (12.66 to 21.64) | 185.15 (154.67 to 265.43) | -19.96% (-30.86% to 5.72%) | 0.5(0.58 to 0.43) | 12(10.23 to 14.31) | -23.05%(-7.47% to -34.62%) |
| Andorra | 0.11 (0.09 to 0.15) | 84 (63.15 to 108.67) | 5.09% (-31.75% to 56.49%) | 0.11 (0.08 to 0.13) | 74.5 (56.73 to 94.77) | -21.16% (-45.59% to 13.45%) | 0.23 (0.17 to 0.33) | 162.5 (121.76 to 232.09) | -10.79% (-38.3% to 21.43%) | 0.01(0.01 to 0.01) | 9.26(6.15 to 13.12) | -28.55%(16.44% to -57.78%) |
| Austria | 11.41 (10.47 to 12.34) | 69.91 (64.66 to 75.48) | -38.42% (-43.33% to -32.82%) | 11.62 (10.48 to 13.02) | 63.78 (57.48 to 71.32) | -27.51% (-34.14% to -19.97%) | 27.8 (22.98 to 44.1) | 147.57 (122.33 to 234.77) | -7.49% (-22.14% to 31.06%) | 0.99(1.21 to 0.81) | 11.12(8.83 to 14.39) | -20.69%(5.36% to -38.34%) |
| Belgium | 15.74 (14.11 to 17.59) | 76.3 (68.25 to 84.83) | -11.3% (-21.49% to -0.33%) | 20.68 (18.42 to 23.14) | 88.92 (79.03 to 99.21) | -30.06% (-37.24% to -22.14%) | 39.2 (32.04 to 65.87) | 160.11 (130.67 to 271.09) | -25.36% (-40.19% to 19.67%) | 0.65(0.88 to 0.51) | 5.86(4.35 to 8.65) | -37.29%(-11.24% to -52.87%) |
| Cyprus | 0.85 (0.73 to 0.97) | 46.04 (39.38 to 52.83) | 75.7% (37.96% to 118.88%) | 1.69 (1.46 to 1.95) | 85.71 (73.8 to 99.02) | -2.82% (-20.81% to 20.09%) | 3.31 (2.73 to 4.13) | 165.71 (136.67 to 205.15) | 2.72% (-21.03% to 29.41%) | 0.06(0.09 to 0.04) | 4.49(3.2 to 6.16) | -42.32%(-7.14% to -62.32%) |
| Denmark | 9.41 (8.4 to 10.55) | 90.52 (81.19 to 100.63) | 38.02% (22.52% to 55.93%) | 12.31 (10.81 to 13.89) | 105.01 (92.61 to 117.95) | -9.18% (-19.68% to 2.75%) | 27.7 (21.62 to 35.46) | 225.14 (174.64 to 285.59) | -7.04% (-18.3% to 7.98%) | 0.67(0.85 to 0.55) | 11.55(9.18 to 15.53) | -15.36%(6.33% to -32.9%) |
| Finland | 9.49 (8.51 to 10.61) | 86.18 (77.28 to 96.16) | -13.02% (-22.59% to -1.51%) | 5.8 (5.1 to 6.58) | 45.95 (40.53 to 51.88) | -34.91% (-42.73% to -26.01%) | 22.39 (18.3 to 31.9) | 169.99 (139.21 to 240.62) | 4.15% (-11.5% to 36.49%) | 0.37(0.46 to 0.3) | 7.35(5.65 to 10.04) | -8.45%(22.72% to -32.67%) |
| France | 100.29 (89.31 to 111.98) | 85.18 (75.98 to 94.56) | -9.62% (-19.19% to 0.98%) | 124.74 (110.19 to 141.73) | 91.48 (81.14 to 103.81) | -23.57% (-31.44% to -13.69%) | 215.94 (175.63 to 342.89) | 145.6 (118.51 to 235.8) | -30.4% (-43.03% to 7.41%) | 6.9(8.47 to 5.64) | 10.88(8.69 to 13.97) | -20.82%(-1.14% to -38.17%) |
| Germany | 150.48 (134.77 to 166.66) | 87.08 (78.26 to 95.43) | -10.28% (-20.43% to -0.41%) | 139.99 (125.54 to 154.72) | 71.87 (64.59 to 79.2) | -30.38% (-37.41% to -23.2%) | 331.7 (254.78 to 426.68) | 162.12 (123.64 to 204.57) | 8.21% (-9.25% to 23.58%) | 10.23(12.42 to 8.6) | 12.09(9.76 to 15.32) | -37.5%(-19.73% to -49.48%) |
| Greece | 14.62 (13.31 to 16.02) | 72.99 (66.89 to 79.6) | 8.59% (-1.76% to 20.16%) | 27.75 (25.17 to 30.6) | 116.13 (105.53 to 128.04) | -18.79% (-25.81% to -10.81%) | 37.84 (31.72 to 54.46) | 140.87 (118.59 to 207.46) | -11.15% (-22.12% to 2.32%) | 1.08(1.36 to 0.91) | 11.13(8.81 to 14.82) | -21.95%(0.84% to -39.12%) |
| Iceland | 0.58 (0.5 to 0.66) | 113.24 (98.84 to 130.03) | -6.14% (-20.71% to 10.79%) | 0.36 (0.31 to 0.42) | 64.64 (55.81 to 74.06) | -30.38% (-40.8% to -18.2%) | 1.03 (0.82 to 1.32) | 177.63 (142.77 to 227.31) | -19.45% (-31.3% to -7.25%) | 0.01(0.01 to 0.01) | 2.59(1.66 to 4.33) | -61.88%(-33.36% to -74.44%) |
| Ireland | 5.97 (5.27 to 6.69) | 84.36 (74.76 to 93.98) | 12.64% (-2.32% to 28.32%) | 4.91 (4.18 to 5.7) | 64.71 (55.22 to 74.93) | -18.31% (-31.54% to -4.67%) | 13.37 (10.94 to 18.51) | 172.59 (141.21 to 239.4) | -15.55% (-29.15% to 11.48%) | 0.36(0.48 to 0.29) | 7.75(5.94 to 10.65) | -36.34%(-13.92% to -52.02%) |
| Israel | 7.23 (6.44 to 8.07) | 65.7 (58.55 to 72.9) | -1.32% (-13.72% to 12.9%) | 9.12 (8.09 to 10.18) | 77.75 (69.34 to 86.74) | -8.68% (-18.15% to 2.57%) | 12.1 (9.81 to 20.17) | 100.79 (82.12 to 167.29) | -27.16% (-39.81% to -1.08%) | 0.46(0.62 to 0.37) | 5.01(3.97 to 6.77) | -3.81%(26.46% to -25.45%) |
| Italy | 95.45 (88.51 to 101.22) | 78.03 (73.3 to 82.21) | -12.1% (-16.72% to -7.41%) | 140.26 (126.59 to 154.55) | 94.91 (87.09 to 104.57) | -32.88% (-36.89% to -28.26%) | 179.81 (150.1 to 265.65) | 115.23 (97.45 to 172.22) | -14.64% (-23.78% to 4.08%) | 5.99(7.1 to 5.15) | 10.57(8.79 to 13.17) | -2.87%(16.43% to -20.86%) |
| Luxembourg | 0.37 (0.32 to 0.43) | 39.27 (33.71 to 46.51) | -26.05% (-37.38% to -11.18%) | 0.79 (0.67 to 0.93) | 78.17 (65.87 to 92.54) | -29.31% (-40.06% to -17.29%) | 1.37 (1.07 to 2.25) | 132.84 (104.06 to 220.53) | -29.09% (-45.42% to 20.69%) | 0.06(0.09 to 0.05) | 9.77(7.5 to 13.94) | -46.92%(-22.86% to -60.6%) |
| Malta | 0.56 (0.47 to 0.66) | 67.88 (57.42 to 79.75) | -2.98% (-19.13% to 17.64%) | 0.71 (0.6 to 0.84) | 75.92 (63.81 to 88.76) | -32.97% (-44.24% to -20.2%) | 0.98 (0.8 to 1.5) | 98.7 (79.75 to 150.84) | -23.99% (-38.37% to -1.68%) | 0.05(0.07 to 0.04) | 12.66(9.96 to 16.78) | -2.83%(27.01% to -25.04%) |
| Netherlands | 29.99 (26.78 to 33.17) | 95.18 (85.36 to 105.05) | 1.28% (-9.48% to 12.66%) | 33.61 (29.94 to 37.59) | 97.79 (87.39 to 109.15) | -13.99% (-22.46% to -4.18%) | 69.06 (52.59 to 84.88) | 190.32 (145.04 to 235.92) | 1.7% (-14.24% to 15.7%) | 1.59(1.98 to 1.3) | 9.67(7.55 to 12.83) | -12.31%(11.73% to -30.1%) |
| Norway | 7.76 (7.24 to 8.28) | 87.63 (81.96 to 93.5) | -2.32% (-8.48% to 3.93%) | 7.18 (6.45 to 7.98) | 73.29 (65.97 to 81.42) | -30.21% (-36.45% to -23.29%) | 21.94 (17.86 to 29.33) | 216.49 (178.06 to 292.13) | -8.7% (-21.42% to 16.62%) | 0.44(0.56 to 0.37) | 8.43(6.84 to 11.1) | -39.05%(-24.3% to -47.94%) |
| Portugal | 10.22 (9.16 to 11.4) | 51.95 (46.41 to 57.77) | -9.04% (-19.4% to 2.67%) | 19.97 (17.95 to 22.14) | 82.86 (74.64 to 92.09) | -9.77% (-19.19% to 0.11%) | 40.97 (34.2 to 60.99) | 156.32 (130.31 to 234.52) | -17.81% (-30.28% to 10.9%) | 0.81(0.98 to 0.68) | 7.65(6.19 to 10.14) | -14.19%(13.39% to -36.09%) |
| Spain | 58.88 (52.75 to 64.73) | 71.64 (64.74 to 78.81) | 17.02% (5.45% to 29.11%) | 106.16 (95.08 to 119.59) | 109.64 (98.04 to 123.29) | -23.85% (-31.05% to -14.06%) | 125.14 (104.65 to 185.47) | 120.55 (100.81 to 181.84) | -22.99% (-35.22% to 5.7%) | 2.63(3.23 to 2.15) | 5.85(4.57 to 7.7) | -22.74%(-0.6% to -40.8%) |
| Sweden | 15.81 (14.47 to 17.07) | 83.56 (77.34 to 89.59) | -31.99% (-37.51% to -26.75%) | 15.33 (13.91 to 16.77) | 70.61 (64.56 to 77.01) | -4.02% (-11.53% to 3.9%) | 47.8 (39.96 to 63.31) | 210.46 (175.35 to 275.3) | -11.63% (-20.97% to 8.05%) | 0.66(0.84 to 0.54) | 6.82(5.27 to 9.27) | -25.13%(-4.18% to -39.99%) |
| Switzerland | 9.14 (8.15 to 10.07) | 57.72 (51.54 to 63.87) | 33.59% (18.87% to 49.82%) | 11.01 (9.8 to 12.4) | 62.1 (55.55 to 69.99) | 14.58% (2.05% to 30.46%) | 30.35 (24.54 to 48.35) | 163.3 (131.38 to 260.14) | -34.38% (-47.78% to 15.38%) | 0.83(1.02 to 0.68) | 9.32(7.41 to 12.14) | -20.82%(1.99% to -38.68%) |
| United Kingdom | 99.09 (94.25 to 103.48) | 88.59 (84.51 to 92.4) | 3.34% (-1.26% to 8%) | 110.47 (102.54 to 116.77) | 85.73 (80.18 to 90.44) | -31.24% (-34.52% to -28.11%) | 248.19 (204.32 to 325.41) | 183.72 (152.37 to 241.5) | -1.1% (-12.92% to 17.78%) | 4.92(6.43 to 4.25) | 7.21(6.06 to 9.87) | -51.06%(-29.84% to -56.3%) |
| Argentina | 67.22 (60.33 to 74.4) | 130.01 (116.68 to 144) | -19.03% (-27.91% to -9.02%) | 39.12 (35.79 to 42.56) | 72.3 (66.27 to 78.61) | -24.35% (-31.15% to -16.77%) | 106.6 (91.05 to 150.68) | 192.08 (164.06 to 270.81) | -1.98% (-11.63% to 9.42%) | 14.9(17.45 to 12.64) | 31.97(26.96 to 37.74) | 15.81%(45.45% to -6.53%) |
| Chile | 24.5 (22.25 to 26.93) | 104.31 (94.65 to 114.3) | -3.08% (-12.52% to 7.67%) | 12.72 (11.29 to 14.08) | 52.74 (46.82 to 58.38) | -5.88% (-17.71% to 6.29%) | 45.85 (34.23 to 54.66) | 189.55 (141.46 to 226.09) | 9.25% (-11.47% to 22.7%) | 7.17(8.84 to 5.99) | 38.26(31.46 to 48.55) | -13.17%(10.42% to -30.26%) |
| Uruguay | 7.87 (7.05 to 8.71) | 166.63 (149.92 to 184.54) | -3.72% (-14.1% to 8.15%) | 5.12 (4.58 to 5.68) | 94.68 (84.93 to 105.25) | -20.22% (-29.36% to -10.22%) | 13.27 (10.86 to 16.85) | 231.3 (188.45 to 293.85) | -1.16% (-11.12% to 10.91%) | 0.82(0.99 to 0.68) | 24.21(19.84 to 29.74) | -13.76%(8.98% to -31.83%) |
| Canada | 46.78 (41.8 to 52.1) | 73.98 (66.25 to 81.63) | 13.75% (2.01% to 26.61%) | 49.54 (44.46 to 54.9) | 71.05 (64 to 78.5) | -27.8% (-34.1% to -20.6%) | 106.89 (90.2 to 157.89) | 148 (125.2 to 219.22) | -31.25% (-42.13% to -3.31%) | 2.66(3.32 to 2.12) | 7.81(5.89 to 10.28) | -12.44%(9.65% to -30.93%) |
| United States of America | 434.45 (409.38 to 456.57) | 84.02 (79.34 to 88.17) | -10.93% (-16.89% to -6.02%) | 384.24 (359.03 to 406.94) | 68.13 (63.98 to 72.08) | -1.69% (-5.76% to 2.53%) | 926.63 (801.16 to 1360.98) | 160 (138.59 to 234.77) | -24.56% (-33.62% to 3.49%) | 26.56(30.56 to 23.53) | 8.11(7.03 to 9.8) | -20.97%(-13.04% to -27.77%) |
| Antigua and Barbuda | 0.05 (0.04 to 0.06) | 52.94 (43.59 to 63.79) | -17.06% (-33.4% to 3%) | 0.05 (0.04 to 0.06) | 51.96 (43.4 to 61.24) | 8.67% (-10.8% to 30.9%) | 0.54 (0.41 to 0.67) | 575.1 (439.78 to 713.02) | 13.91% (-6.35% to 40.14%) | 0.01(0.01 to 0) | 5.79(4.46 to 7.28) | 271.24%(392.68% to 158.26%) |
| Bahamas | 0.25 (0.2 to 0.32) | 62.08 (49.57 to 77.49) | -23.16% (-39.55% to -2.22%) | 0.14 (0.11 to 0.17) | 36.22 (29.31 to 44.01) | -5.1% (-24.44% to 18.96%) | 1.82 (1.4 to 2.45) | 512.56 (397.28 to 693.82) | 7.51% (-12.59% to 32.52%) | 0(0.01 to 0) | 1.26(0.95 to 1.63) | 126.71%(212.01% to 45.95%) |
| Barbados | 0.33 (0.26 to 0.4) | 76.07 (60.79 to 92.54) | -23.1% (-39.34% to -6.13%) | 0.25 (0.2 to 0.29) | 49.92 (40.68 to 59.63) | 10.32% (-12.11% to 33.49%) | 2.93 (2.15 to 3.59) | 578.95 (424.59 to 707.79) | 34.58% (9.19% to 62.39%) | 0.01(0.01 to 0.01) | 3.05(2.31 to 3.87) | 220.14%(331.73% to 130.69%) |
| Belize | 0.19 (0.16 to 0.22) | 55.62 (46.89 to 65.29) | -5.83% (-22.83% to 15.65%) | 0.11 (0.1 to 0.13) | 41.9 (35.94 to 48.5) | 30.62% (8.05% to 56.22%) | 0.8 (0.63 to 1.04) | 328.96 (258.31 to 430.1) | 50.57% (5.17% to 98.83%) | 0.02(0.03 to 0.02) | 5.48(4.25 to 6.94) | 413.61%(621.43% to 226.39%) |
| Cuba | 9.04 (7.19 to 11.24) | 53.87 (42.99 to 66.81) | -28.54% (-42.83% to -11.47%) | 14.58 (11.63 to 18.06) | 76.45 (60.92 to 94.65) | 7.91% (-12.97% to 34.49%) | 56.06 (33.33 to 72.32) | 286.62 (170.16 to 370.7) | 19.37% (-21.22% to 53.34%) | 0.77(0.98 to 0.54) | 6.38(4.48 to 8.21) | 224.54%(344.09% to 119.91%) |
| Dominica | 0.06 (0.05 to 0.07) | 72.89 (56.41 to 92.42) | -8% (-29.99% to 20.34%) | 0.06 (0.05 to 0.08) | 71.24 (58.81 to 88.01) | 10.42% (-11.43% to 37.96%) | 0.82 (0.61 to 1.02) | 888.79 (662.33 to 1108.44) | 41.61% (4.69% to 86.02%) | 0(0 to 0) | 4.3(2.97 to 6.03) | 305.49%(501.02% to 173.58%) |
| Dominican Republic | 7.36 (5.02 to 10) | 72.84 (50.01 to 98.35) | 48.5% (1.48% to 106.65%) | 2.53 (1.84 to 3.45) | 27.63 (20.17 to 37.51) | 33.34% (-5.93% to 88.35%) | 28.83 (18.9 to 47.68) | 328.02 (216.77 to 539.35) | 36.8% (-6.24% to 104.34%) | 0.26(0.43 to 0.16) | 2.36(1.47 to 3.88) | 50.8%(125.66% to -5.84%) |
| Grenada | 0.06 (0.06 to 0.07) | 55.83 (48.6 to 63.29) | -3.12% (-17.45% to 14.52%) | 0.07 (0.06 to 0.08) | 60.97 (53.47 to 69.06) | 13.67% (-5.32% to 34.92%) | 0.73 (0.49 to 0.85) | 675.78 (452.79 to 782.34) | 36.27% (-6.46% to 67.94%) | 0(0 to 0) | 3.19(2.64 to 3.93) | 89.23%(139.12% to 36.46%) |
| Guyana | 0.46 (0.34 to 0.61) | 63.93 (48.04 to 84.32) | -12.67% (-36.17% to 16.81%) | 0.24 (0.19 to 0.31) | 38.81 (29.99 to 49.54) | 0.34% (-24.86% to 32.66%) | 2.43 (1.8 to 3.42) | 444.01 (332.63 to 617.83) | 3.88% (-20.26% to 34.28%) | 0.06(0.08 to 0.04) | 7.77(5.38 to 10.45) | 279.25%(455.66% to 123.79%) |
| Haiti | 3.99 (2.85 to 5.44) | 39.6 (28.02 to 54.56) | -22.33% (-46.62% to 7.24%) | 3.82 (2.35 to 5.63) | 56.66 (35.33 to 82.8) | -12.2% (-34.48% to 19.6%) | 25.36 (17.33 to 35.66) | 444.54 (307.86 to 608.52) | 8.79% (-17.99% to 44.96%) | 0.49(0.78 to 0.3) | 3.86(2.38 to 5.88) | 53.81%(124.9% to 6.33%) |
| Jamaica | 1.05 (0.78 to 1.37) | 35.91 (27.02 to 46.62) | -28.89% (-48.13% to -5.04%) | 1.38 (1.07 to 1.74) | 46.47 (36 to 58.56) | -6.54% (-27.83% to 18.29%) | 14.09 (7.84 to 18.29) | 478.29 (263.2 to 622.22) | 86.23% (17.19% to 144.84%) | 0.09(0.12 to 0.06) | 2.89(2.05 to 4.38) | 214.27%(329.18% to 89.95%) |
| Saint Lucia | 0.1 (0.08 to 0.12) | 46.64 (38.01 to 56.62) | -27.42% (-41.93% to -10.73%) | 0.14 (0.11 to 0.16) | 64.09 (53.58 to 76.12) | -11.81% (-27.53% to 6.06%) | 1.14 (0.92 to 1.53) | 542.87 (439.08 to 731.6) | 11.57% (-7.22% to 33.9%) | 0.02(0.02 to 0.01) | 10.56(8.04 to 13.52) | 260.64%(382.94% to 157.09%) |
| Saint Vincent and the Grenadines | 0.07 (0.05 to 0.08) | 50.05 (42.06 to 58.9) | -28.65% (-40.83% to -14.34%) | 0.07 (0.06 to 0.08) | 52.72 (45.81 to 61.08) | 11.77% (-5.53% to 32.15%) | 0.81 (0.63 to 1.01) | 617.4 (481.52 to 767.73) | 45.92% (9.42% to 83.29%) | 0.01(0.01 to 0) | 4.62(3.61 to 6.06) | 247.72%(353.82% to 129.68%) |
| Suriname | 0.32 (0.26 to 0.39) | 52.54 (41.88 to 64.48) | -14.19% (-32.65% to 10.32%) | 0.22 (0.18 to 0.27) | 37.34 (30.8 to 44.79) | 13.43% (-8.08% to 38.84%) | 1.67 (1.24 to 2.09) | 295.78 (220.39 to 368.5) | 24.9% (-12.86% to 68.76%) | 0.05(0.08 to 0.03) | 9.26(6.01 to 13.85) | 240.45%(503.8% to 92.73%) |
| Trinidad and Tobago | 0.9 (0.66 to 1.21) | 53.82 (39.7 to 71.41) | -42.25% (-57.77% to -22.76%) | 0.65 (0.48 to 0.87) | 35.05 (25.97 to 46.51) | -17.67% (-40.21% to 9.34%) | 7.08 (4.66 to 9.52) | 387.25 (255.49 to 518.04) | -3.43% (-28.53% to 25.8%) | 0.08(0.12 to 0.05) | 7.04(4.49 to 9.94) | 228.3%(383.1% to 60.45%) |
| Bolivia (Plurinational State of) | 5.27 (3.96 to 6.87) | 54.61 (41.34 to 70.76) | 38.49% (-1.49% to 88.55%) | 4.11 (3.18 to 5.2) | 48.34 (37.74 to 60.7) | -0.09% (-24.43% to 33.66%) | 22.45 (16.49 to 29) | 281.11 (208.1 to 361.17) | 28.35% (-2.94% to 70.71%) | 2.02(2.88 to 1.21) | 16.37(10.06 to 23.22) | 51.46%(146.79% to -22.83%) |
| Ecuador | 8.36 (6.54 to 10.68) | 52.63 (41.31 to 67.13) | 33.16% (3.88% to 71.55%) | 4.37 (3.42 to 5.55) | 29.72 (23.36 to 37.59) | 0.91% (-21.02% to 27.59%) | 26.7 (20.05 to 36.24) | 189.15 (141.34 to 255.32) | 5.8% (-20.21% to 37.24%) | 3.26(4.49 to 2.3) | 18.15(12.9 to 24.69) | 255.21%(435.24% to 140.68%) |
| Peru | 17.5 (12.63 to 23.43) | 53.83 (39 to 72.42) | -9.1% (-35.62% to 26.5%) | 7.82 (5.85 to 10.4) | 24.5 (18.37 to 32.5) | -22.02% (-43.05% to 7.95%) | 51.88 (37.35 to 71.11) | 165.16 (118.42 to 226.36) | -1.68% (-32.32% to 39.13%) | 5.48(8.25 to 3.28) | 15.64(9.49 to 23.63) | -20.86%(50.18% to -60.38%) |
| Colombia | 20.23 (15.35 to 26.27) | 40.1 (30.53 to 51.84) | 16.51% (-12.12% to 51.97%) | 13.4 (10.25 to 17.37) | 25.37 (19.35 to 32.83) | -36.79% (-51.91% to -18.52%) | 75.32 (54.13 to 116.51) | 142.91 (103.01 to 220.38) | -21.06% (-41.87% to 6.38%) | 7.37(9.99 to 5.37) | 14.72(10.64 to 19.97) | 28.04%(75.36% to -8.97%) |
| Costa Rica | 2.9 (2.19 to 3.8) | 56.43 (42.7 to 74.01) | 56.01% (16.85% to 107.33%) | 1.9 (1.45 to 2.44) | 37.45 (28.63 to 48.06) | -9.08% (-31.04% to 16.55%) | 9.5 (5.96 to 12.77) | 189.63 (119.39 to 254.95) | 26.29% (-12.55% to 69.14%) | 0.75(1.02 to 0.52) | 14.95(10.38 to 20.73) | 94.54%(168.22% to 31.89%) |
| El Salvador | 2.3 (1.7 to 2.99) | 38.95 (28.8 to 50.67) | 35.27% (-1.22% to 78.04%) | 1.24 (0.94 to 1.6) | 20.82 (15.78 to 26.87) | 9.95% (-18.52% to 43.42%) | 8.69 (6.27 to 11.39) | 143.84 (103.58 to 189.17) | 36.14% (-9.58% to 96.54%) | 0.52(0.78 to 0.33) | 7.93(5.19 to 11.84) | 97.29%(235.31% to 14.14%) |
| Guatemala | 5.74 (4.43 to 7.43) | 43.93 (34.1 to 56.62) | 32.36% (0.58% to 73.65%) | 2.31 (1.79 to 2.93) | 20.77 (16.25 to 26.33) | -9.37% (-31.21% to 20.19%) | 20.38 (15.2 to 28.08) | 196.78 (146.21 to 266.93) | 42.71% (-26.52% to 120.29%) | 3.58(4.71 to 2.57) | 18.86(13.61 to 24.64) | 525.47%(837.17% to 282.36%) |
| Honduras | 2.84 (2.09 to 3.86) | 42.02 (31.05 to 58.64) | 62.89% (19.96% to 125.82%) | 1.72 (1.3 to 2.47) | 29.76 (22.71 to 42.88) | 39.55% (3.58% to 99.25%) | 9.19 (6.77 to 13.4) | 170.79 (125.57 to 250.9) | 36.04% (-0.47% to 106.07%) | 0.38(0.62 to 0.22) | 3.93(2.35 to 6.44) | -15.83%(44.98% to -51.78%) |
| Mexico | 94.01 (79.44 to 109.88) | 77.55 (65.61 to 90.34) | 32.54% (11.83% to 54.6%) | 33.73 (28.73 to 39.19) | 29.15 (24.88 to 33.78) | -5.36% (-18.72% to 10.34%) | 161.55 (122.76 to 219.51) | 146.22 (111.6 to 198.3) | -1.33% (-18.26% to 19.06%) | 36.62(49.88 to 28.54) | 28.28(21.97 to 40.15) | 47.9%(79.83% to 11.01%) |
| Nicaragua | 2.28 (1.82 to 2.84) | 45.79 (36.57 to 56.63) | 48.02% (12.05% to 90.39%) | 0.92 (0.76 to 1.11) | 21.89 (18.13 to 26.09) | 6.33% (-14.96% to 37.03%) | 8.58 (6.6 to 11.21) | 222.85 (173.07 to 292.11) | 25.4% (-6.13% to 63.62%) | 0.68(1.01 to 0.44) | 10.17(6.77 to 14.93) | 141.28%(311.08% to 42.99%) |
| Panama | 2.17 (1.61 to 2.83) | 52.4 (39.07 to 68.18) | 65.82% (23.35% to 121.17%) | 1 (0.75 to 1.28) | 24.09 (18.19 to 30.93) | -20.24% (-40.39% to 4.08%) | 8.03 (5.37 to 10.75) | 195.66 (130.77 to 262.03) | 16.82% (-14.65% to 56.18%) | 0.43(0.59 to 0.29) | 10.18(6.92 to 14.13) | 278.87%(492.02% to 105.66%) |
| Venezuela (Bolivarian Republic of) | 21.84 (15.96 to 29.07) | 72.89 (53.41 to 96.79) | -5.54% (-31.19% to 26.23%) | 11.11 (8.29 to 14.35) | 38.62 (29.09 to 49.8) | 5.42% (-21.49% to 38.33%) | 77.07 (50.48 to 103.62) | 277.48 (184.06 to 372.52) | 41.43% (-5.99% to 94.94%) | 2.55(3.35 to 1.8) | 8.97(6.33 to 11.74) | 339.28%(516.04% to 181.16%) |
| Brazil | 124.14 (117.59 to 130.54) | 53.46 (50.59 to 56.2) | 19.88% (11.62% to 28.36%) | 107.76 (99.75 to 114.56) | 46.04 (42.47 to 49.01) | -16.33% (-21.87% to -10.81%) | 413.23 (359.72 to 605.64) | 180.23 (156.51 to 263.47) | -7.9% (-13.65% to -1.89%) | 23.19(25.91 to 21.52) | 10.2(9.4 to 12.03) | 19.87%(34.42% to -8.45%) |
| Paraguay | 2.77 (2.01 to 3.64) | 46.56 (33.76 to 60.88) | 10.01% (-22.22% to 52.01%) | 1.46 (1.1 to 1.9) | 26.8 (20.29 to 34.67) | 18.2% (-15.43% to 65.56%) | 9.91 (6.45 to 13.59) | 190.5 (123.55 to 260.63) | 47.71% (-5.91% to 114.06%) | 1.33(1.89 to 0.89) | 18.02(12.06 to 25.29) | 314.21%(545.02% to 162.35%) |
| Algeria | 8.71 (6.84 to 10.87) | 23.4 (18.3 to 29.08) | 22.88% (-9.16% to 65.18%) | 12.2 (9.61 to 15.23) | 39.42 (31.35 to 48.96) | -22.53% (-45.27% to 4.94%) | 22.2 (16.36 to 30.44) | 78.24 (58.69 to 106.97) | -13.12% (-34.5% to 14.42%) | 1.29(1.74 to 0.94) | 2.93(2.14 to 3.91) | -2.16%(50.1% to -36.81%) |
| Bahrain | 0.58 (0.44 to 0.74) | 48.81 (37.69 to 61.35) | -11.64% (-35.3% to 18.39%) | 0.59 (0.44 to 0.77) | 82.2 (63.71 to 102.99) | -43.3% (-58.34% to -22.71%) | 0.83 (0.61 to 1.15) | 126.94 (95.54 to 164.99) | -7.79% (-33% to 24.48%) | 0.01(0.02 to 0.01) | 1.16(0.85 to 1.58) | 58.12%(130.82% to 9.9%) |
| Egypt | 22.79 (16.24 to 31.57) | 29.26 (20.69 to 41.41) | 53.35% (7.34% to 121.74%) | 146.01 (94.86 to 213.43) | 201.75 (132.14 to 294.39) | 4.63% (-31.99% to 56.97%) | 35.59 (25.03 to 49.72) | 68.51 (49.61 to 95) | 31.9% (-3.35% to 84.7%) | 2.79(4.18 to 1.81) | 2.73(1.78 to 4.04) | 13.02%(87.03% to -31.83%) |
| Iran (Islamic Republic of) | 26.59 (24.23 to 28.54) | 34.36 (31.11 to 36.96) | 28.07% (0.33% to 83.86%) | 35.67 (33.01 to 38.86) | 50.18 (46.19 to 54.68) | 0.71% (-16.3% to 33.23%) | 67.73 (50.16 to 76.61) | 104.12 (77.61 to 117.95) | 16.86% (-2.81% to 44.84%) | 4.94(5.76 to 4.11) | 5.67(4.68 to 6.64) | 176.13%(294.22% to 91.92%) |
| Iraq | 13.89 (10.45 to 17.72) | 49.21 (37.05 to 62.46) | 39.86% (-12.01% to 138.48%) | 28.58 (21.82 to 35.37) | 132.62 (103.08 to 161.3) | 23.37% (-14.84% to 86.71%) | 16.16 (12.16 to 20.98) | 85.87 (64.79 to 112.97) | 25.39% (-9.43% to 75.76%) | 1.98(3.05 to 1.32) | 4.65(3.18 to 6.97) | 14%(92.8% to -32.82%) |
| Jordan | 2.69 (2.17 to 3.27) | 34.19 (27.57 to 41.54) | 64.22% (15.7% to 122.03%) | 4.15 (3.29 to 5.24) | 67.73 (54.12 to 84.66) | -11.33% (-34.95% to 25.13%) | 4.85 (3.42 to 6.32) | 94.3 (66.14 to 122.01) | 10.3% (-17.32% to 48.62%) | 0.74(1.03 to 0.52) | 6.27(4.5 to 8.55) | 9.25%(80.91% to -32.12%) |
| Kuwait | 1.16 (0.92 to 1.45) | 37.57 (29.82 to 47.2) | 3.69% (-17.84% to 31.8%) | 1.49 (1.18 to 1.86) | 66.1 (53.24 to 82.19) | -14.41% (-32.76% to 8.87%) | 1.93 (1.42 to 2.6) | 99.64 (73.35 to 132.41) | 34.52% (7.04% to 67.23%) | 0.08(0.13 to 0.06) | 1.89(1.26 to 3.05) | -52.33%(-20.5% to -66.94%) |
| Lebanon | 2.94 (2.18 to 3.98) | 56.61 (42.13 to 76.93) | 71.99% (16.32% to 167.68%) | 10.05 (7.74 to 13.26) | 192.75 (147.89 to 254.9) | -14.51% (-37.92% to 25.48%) | 9.81 (6.23 to 13.91) | 186.45 (119.37 to 264.69) | 11.58% (-21.19% to 55.69%) | 0.39(0.61 to 0.25) | 7.21(4.61 to 11.15) | 53%(163.14% to -12.33%) |
| Libya | 3.04 (1.86 to 4.13) | 55.41 (33.88 to 75.39) | 39.6% (-37.42% to 169.69%) | 5.05 (3.71 to 6.58) | 104.45 (77.27 to 135.24) | 0.58% (-34.22% to 67.79%) | 3.73 (2.74 to 4.88) | 86.32 (63.9 to 112.6) | 8.32% (-23.73% to 66.81%) | 0.22(0.33 to 0.14) | 2.96(1.99 to 4.39) | 13.58%(97.51% to -34.59%) |
| Morocco | 7.65 (5.57 to 9.8) | 22.69 (16.57 to 28.82) | 60.04% (14.74% to 120.91%) | 13.63 (10.17 to 17.52) | 45.58 (34.71 to 58.1) | 18.65% (-15% to 63.92%) | 21.44 (15.51 to 27.94) | 79.6 (58.29 to 104.48) | 31.17% (-3.23% to 69.78%) | 0.88(1.37 to 0.56) | 2.43(1.53 to 3.67) | 14.03%(95.91% to -34.05%) |
| Palestine | 1.2 (1.01 to 1.43) | 40.3 (33.75 to 48.09) | 35.25% (-7.38% to 149.3%) | 1.52 (1.28 to 1.83) | 68.92 (58.14 to 82.7) | -10.72% (-37.29% to 41.06%) | 2.35 (1.59 to 2.97) | 122.61 (81.12 to 155.75) | -6.05% (-33.41% to 43.84%) | 0.07(0.09 to 0.05) | 1.41(1.05 to 1.82) | 102.77%(227.17% to 24.24%) |
| Oman | 0.77 (0.58 to 0.94) | 33 (25.99 to 38.74) | 80.28% (11.88% to 188.19%) | 0.68 (0.53 to 0.87) | 45.48 (37.8 to 54.4) | 0.06% (-30.97% to 60.26%) | 1.23 (0.97 to 1.65) | 99.35 (80.62 to 127.48) | 18.51% (-15.39% to 84%) | 0.09(0.13 to 0.06) | 1.76(1.13 to 2.45) | 31.26%(114.18% to -17.34%) |
| Qatar | 0.71 (0.49 to 1.01) | 72.83 (49.97 to 100.16) | 17.15% (-29.8% to 107.6%) | 0.38 (0.26 to 0.52) | 78.21 (58.16 to 102.65) | 27.37% (-13.79% to 92.29%) | 0.85 (0.56 to 1.28) | 202.5 (146.59 to 273.99) | 74.83% (26.63% to 151.95%) | 0.08(0.12 to 0.05) | 2.41(1.51 to 3.53) | 9.29%(76.26% to -32.57%) |
| Saudi Arabia | 10 (7.41 to 13.45) | 40.64 (31.42 to 52.31) | 118.89% (40.96% to 299.46%) | 5.81 (4.42 to 7.72) | 32.93 (26.21 to 41.74) | -11.28% (-40.57% to 55.53%) | 11.35 (8.49 to 16.85) | 87.57 (66.19 to 131.22) | -2.89% (-33.89% to 100.95%) | 1.16(1.73 to 0.76) | 2.84(1.95 to 4.05) | 92.76%(224.35% to 17.52%) |
| Syrian Arab Republic | 2.4 (1.69 to 3.28) | 17.66 (12.45 to 24.04) | 40.03% (-12.36% to 122.91%) | 4.79 (3.51 to 6.46) | 40.1 (29.65 to 53.06) | 4.1% (-30.68% to 65.24%) | 8.71 (6 to 13.82) | 85.36 (58.91 to 137.04) | 6.81% (-27.42% to 66.49%) | 0.3(0.44 to 0.2) | 2.26(1.52 to 3.27) | 14.38%(90.4% to -31.38%) |
| Tunisia | 3.72 (2.6 to 5.12) | 29.27 (20.57 to 40) | 46.86% (-0.39% to 123.46%) | 9.65 (6.9 to 13.46) | 78.52 (56.45 to 108.69) | -5.31% (-37.66% to 47.85%) | 8.22 (5.84 to 11.75) | 69.71 (49.58 to 99.17) | 1.13% (-31% to 50.83%) | 0.26(0.39 to 0.17) | 2.33(1.5 to 3.41) | 15.7%(86.84% to -31.37%) |
| Turkey | 46.26 (36.45 to 58.27) | 52.75 (41.81 to 65.84) | 8.78% (-23.18% to 52.4%) | 65.17 (51.66 to 79.85) | 74.31 (59.18 to 90.76) | -14.89% (-36.37% to 20.03%) | 89.47 (63.88 to 116.48) | 106.5 (75.93 to 137.19) | -12.33% (-35.95% to 18.16%) | 11.98(16.48 to 8.68) | 14.55(10.6 to 20.27) | -11.97%(39.99% to -44%) |
| United Arab Emirates | 9.42 (3.8 to 15.7) | 123.66 (47.09 to 197.48) | 63.87% (-26.89% to 198.66%) | 5.5 (2.98 to 9.35) | 114.98 (61.28 to 197.5) | -22.98% (-57.94% to 42.05%) | 4 (2.15 to 7.47) | 149.16 (86.34 to 289.01) | 20.42% (-15.86% to 67.47%) | 0.77(1.52 to 0.41) | 6.38(3.85 to 11.33) | 59.7%(206.91% to -6.68%) |
| Yemen | 3.92 (2.56 to 5.62) | 20.44 (13.44 to 29.26) | 36.24% (-16.68% to 125.66%) | 7.4 (5.26 to 10.05) | 57.82 (41.32 to 77.83) | -3.06% (-35.19% to 61.39%) | 10.18 (7.4 to 13.69) | 92.84 (68.03 to 123.65) | 40.31% (4.36% to 102.93%) | 0.74(1.1 to 0.47) | 2.41(1.51 to 3.48) | 87.28%(221.72% to 16.33%) |
| Afghanistan | 6.02 (3.96 to 8.71) | 27.25 (17.98 to 38.97) | 9.15% (-24.78% to 58.56%) | 9.15 (6.29 to 12.73) | 74.6 (51.27 to 100.25) | -13.82% (-37.92% to 18.25%) | 9.39 (7.11 to 11.93) | 99.64 (74.44 to 130.04) | -5.45% (-27.23% to 20.5%) | 1.08(1.59 to 0.7) | 2.82(1.85 to 4.18) | 60.36%(155.35% to -4.15%) |
| Bangladesh | 21.74 (14.4 to 29.84) | 15.62 (10.34 to 21.47) | 12.81% (-19.93% to 67.55%) | 28.32 (19.77 to 38.14) | 22.22 (15.61 to 29.76) | -16.24% (-41.57% to 12.34%) | 74.56 (42.73 to 115.07) | 62.59 (36.42 to 96.04) | -17.58% (-40.71% to 9.31%) | 8.45(12.26 to 4.45) | 5.06(2.72 to 7.25) | -23.93%(23.11% to -55.38%) |
| Bhutan | 0.14 (0.07 to 0.21) | 23.4 (12.27 to 34.3) | 66.52% (13.99% to 167.2%) | 0.15 (0.1 to 0.19) | 27.33 (19.08 to 35.77) | 15.06% (-17.67% to 61.06%) | 0.38 (0.21 to 0.62) | 77.11 (42.78 to 123.11) | 48.19% (4.3% to 100.41%) | 0.05(0.08 to 0.02) | 5.54(2.69 to 8.74) | -2.24%(83.29% to -46.9%) |
| India | 244.17 (207.58 to 289.85) | 20.01 (17.02 to 23.74) | 47.85% (14.24% to 97.17%) | 313.59 (268.64 to 366.2) | 28.2 (24.23 to 32.91) | -0.87% (-17.86% to 21.36%) | 580.13 (465.81 to 761.35) | 56.57 (45.74 to 74.15) | -11.48% (-31.49% to 19.87%) | 91.5(109.05 to 75.94) | 6.23(5.19 to 7.41) | -9.53%(14.58% to -29.93%) |
| Nepal | 4.68 (2.73 to 6.93) | 19.47 (11.27 to 28.83) | 70.43% (18.09% to 130.53%) | 5.71 (4.04 to 7.33) | 26.85 (19.11 to 34.59) | 10.65% (-17.87% to 49.29%) | 12.9 (7.66 to 19.62) | 66.18 (39.58 to 100.21) | 19.66% (-15.56% to 67.55%) | 1.15(1.62 to 0.73) | 3.68(2.32 to 5.11) | -23.13%(18.11% to -51.92%) |
| Pakistan | 44.13 (33.81 to 58.93) | 30.95 (23.5 to 41.47) | 76.07% (26.64% to 169.84%) | 139.94 (110.28 to 178.03) | 129.29 (102.16 to 164.53) | 18.1% (-11.84% to 62.55%) | 95.91 (68.73 to 127.16) | 104.52 (76.74 to 136.75) | 7.55% (-23.31% to 62.27%) | 45.89(64.45 to 32.6) | 21.06(14.99 to 29.52) | 36.23%(105.92% to -5.71%) |
| Angola | 5.45 (3.62 to 7.95) | 30.18 (20.95 to 40.97) | 26.92% (-16.57% to 99.64%) | 6.86 (4.7 to 11.88) | 64.91 (44.23 to 114.08) | -10.71% (-33.88% to 30.91%) | 18.44 (12.83 to 23.82) | 214.25 (149.5 to 275.61) | 7.83% (-16.62% to 44.66%) | 1.76(2.27 to 1.25) | 6.86(4.83 to 8.87) | 19.61%(92.86% to -22.97%) |
| Central African Republic | 0.77 (0.53 to 1.07) | 22.83 (15.6 to 31.81) | 3.05% (-30.71% to 39.54%) | 1.26 (0.86 to 1.8) | 58.74 (40.3 to 79.92) | -21.93% (-40.92% to 2.93%) | 3.3 (2.19 to 4.4) | 190.89 (130.24 to 255.02) | -8.4% (-26.36% to 13.08%) | 0.24(0.4 to 0.14) | 5.14(2.98 to 7.93) | 3.19%(53.67% to -29.19%) |
| Congo | 1.28 (0.84 to 1.99) | 37.9 (25.78 to 56.71) | 10.01% (-30.99% to 63.68%) | 1.9 (1.36 to 2.88) | 76.74 (55.23 to 115.4) | -16.93% (-40.26% to 14.82%) | 4.82 (3.33 to 6.17) | 236.44 (163.76 to 299.69) | 5.01% (-14.67% to 30.37%) | 0.33(0.48 to 0.22) | 6.95(4.83 to 9.86) | 11.92%(91.53% to -35.18%) |
| Democratic Republic of the Congo | 10.13 (6.34 to 14.42) | 19.77 (11.87 to 30.01) | -6% (-45.51% to 37.06%) | 21.94 (12.87 to 31.46) | 63.78 (36.59 to 91.87) | -26.41% (-46.5% to 5.44%) | 51.53 (35.16 to 69.01) | 176.63 (120.54 to 236.88) | -13.55% (-33.27% to 10.79%) | 4.11(5.6 to 2.92) | 5.45(3.79 to 7.41) | 13.49%(62.24% to -24.84%) |
| Equatorial Guinea | 0.28 (0.14 to 0.5) | 41.93 (22.7 to 71.01) | 112.84% (-1.48% to 348.48%) | 0.31 (0.2 to 0.44) | 67.98 (45.12 to 94.66) | 9.98% (-27.03% to 78.91%) | 0.84 (0.56 to 1.14) | 218.95 (146.33 to 290.91) | 24.87% (-10.44% to 75.11%) | 0.09(0.15 to 0.05) | 7.14(4.3 to 11.55) | 61.43%(222.64% to -14.36%) |
| Gabon | 0.69 (0.45 to 1.05) | 55.4 (36.65 to 83.32) | 49.82% (-16.99% to 126.63%) | 0.93 (0.62 to 1.7) | 92.61 (62.53 to 167.8) | -8.78% (-38.24% to 35.44%) | 2.28 (1.45 to 3.1) | 251.87 (162.81 to 336.97) | 22.52% (-5.87% to 55.58%) | 0.14(0.2 to 0.09) | 8.06(5.54 to 11.53) | 19.29%(82.18% to -21.21%) |
| Burundi | 1.78 (1.14 to 2.8) | 25.12 (16.91 to 38.2) | 2.87% (-38.76% to 68.34%) | 2.1 (1.48 to 2.94) | 47.14 (33.9 to 65.11) | -32.09% (-61.4% to 3.13%) | 8.99 (5.49 to 12.88) | 232.02 (143.53 to 329.22) | 5.86% (-30.7% to 56.5%) | 0.54(0.76 to 0.36) | 4.8(3.25 to 6.78) | 11.52%(79.46% to -39.49%) |
| Comoros | 0.18 (0.13 to 0.25) | 33.39 (23.67 to 45.41) | 33.12% (-16.42% to 159.11%) | 0.22 (0.15 to 0.3) | 45.77 (30.74 to 62.75) | -8.07% (-32.62% to 57.65%) | 0.99 (0.65 to 1.32) | 220.25 (143.87 to 292.99) | -3.85% (-29.39% to 54.26%) | 0.04(0.05 to 0.02) | 5.17(2.99 to 7.37) | 33.37%(447.19% to -27.73%) |
| Djibouti | 0.35 (0.21 to 0.51) | 43.25 (28.08 to 63.19) | 75.39% (9.43% to 161.43%) | 0.34 (0.23 to 0.5) | 59.98 (42.41 to 83.63) | 6.9% (-20.87% to 46.46%) | 1.47 (0.93 to 2.1) | 296.62 (189.14 to 412.67) | 24.63% (-8.26% to 72.29%) | 0.09(0.13 to 0.05) | 6.98(4.22 to 10.29) | 36.09%(118.36% to -14.65%) |
| Eritrea | 1.24 (0.78 to 1.87) | 32.41 (21.08 to 46.52) | 63.31% (8.64% to 133.9%) | 1.56 (1.15 to 2.1) | 59.01 (44.09 to 78.38) | 4.9% (-25.59% to 47.34%) | 4.39 (2.43 to 6.01) | 191.43 (106.04 to 261.51) | 10.04% (-15.87% to 93.19%) | 0.4(0.63 to 0.23) | 5.88(3.4 to 9.24) | 55.98%(171.16% to -3.14%) |
| Ethiopia | 15.94 (9.49 to 22.07) | 30.07 (16.9 to 43.48) | 2.29% (-30.62% to 63.17%) | 18.47 (12.52 to 24.1) | 47.02 (31.68 to 61.48) | -20.54% (-45.38% to 9.87%) | 43.41 (22.75 to 72.73) | 121.34 (64.24 to 200.25) | 14.49% (-25.32% to 62.97%) | 5.11(6.67 to 3.25) | 4.96(2.98 to 6.52) | 14.39%(90.45% to -41.1%) |
| Kenya | 5.52 (4.17 to 6.96) | 19.7 (14.55 to 24.84) | 87.63% (44.1% to 149.61%) | 6.75 (5.49 to 8.28) | 31.95 (26.25 to 38.82) | 36.2% (-7.25% to 82.07%) | 39.15 (25.39 to 57.79) | 206.47 (136.58 to 296.74) | 44.49% (1.97% to 110.3%) | 0.81(1 to 0.65) | 1.56(1.26 to 1.95) | 35.8%(84.46% to 2.04%) |
| Madagascar | 3.75 (2.6 to 5.19) | 23.83 (16.21 to 32.98) | 13.22% (-23.85% to 57.07%) | 4.63 (3.44 to 6.17) | 43.13 (32.67 to 57.08) | -23.27% (-58.44% to 17.5%) | 16.76 (9.98 to 24.32) | 187.51 (112.25 to 265.9) | -12.32% (-34.66% to 17.01%) | 1.15(1.57 to 0.82) | 4.4(3.16 to 6.07) | -6.97%(44.74% to -41.03%) |
| Malawi | 6.73 (4.84 to 9.12) | 64.44 (47.56 to 84.38) | 23.47% (-16% to 77.9%) | 11.46 (6.58 to 15.54) | 160.24 (93.72 to 213.52) | -5.2% (-48.26% to 32.58%) | 9.22 (7.17 to 12.96) | 137.99 (108.52 to 195.92) | 13.73% (-12.39% to 56.36%) | 0.95(1.29 to 0.68) | 5.42(3.88 to 7.35) | 23.19%(94.85% to -21.81%) |
| Mauritius | 0.61 (0.49 to 0.76) | 37.71 (30.21 to 46.65) | 63.25% (29.82% to 101.89%) | 0.72 (0.58 to 0.91) | 41.53 (33.54 to 51.64) | -39.56% (-52.28% to -23.55%) | 1.75 (1.19 to 2.24) | 104.06 (70.62 to 132.29) | 25.07% (-10.93% to 63.44%) | 0.05(0.06 to 0.04) | 3.6(2.82 to 4.65) | 7.54%(39.52% to -18.61%) |
| Mozambique | 5.44 (3.65 to 7.61) | 27.68 (19.67 to 39.06) | 72.6% (14.89% to 152.51%) | 6.44 (4.74 to 8.34) | 60.23 (45.74 to 76.59) | -3.81% (-53.6% to 56.71%) | 8.35 (5.55 to 14.36) | 87.83 (60.4 to 146.04) | 21% (-12.28% to 74.59%) | 1.57(2.12 to 1.08) | 5.73(3.95 to 7.72) | 58.34%(142.53% to 5.02%) |
| Rwanda | 2.61 (1.9 to 3.5) | 32.84 (24.74 to 43.97) | 16.05% (-20.36% to 87.82%) | 2.86 (2.21 to 3.69) | 49.26 (38.73 to 62.07) | -29.99% (-49.32% to -1.01%) | 11.21 (7.26 to 15.31) | 212.37 (138.76 to 286.88) | -7.2% (-40.34% to 42.69%) | 0.75(1.04 to 0.52) | 5.84(4.02 to 8.11) | 27.22%(118.97% to -36.69%) |
| Seychelles | 0.06 (0.05 to 0.07) | 48.18 (40.77 to 56.82) | 56.63% (29.57% to 88.75%) | 0.09 (0.08 to 0.11) | 88.37 (76.43 to 102.76) | -6.65% (-22.65% to 10.57%) | 0.41 (0.31 to 0.51) | 407.49 (303.85 to 494.72) | 52.35% (-4.69% to 128.5%) | 0.01(0.02 to 0.01) | 8.91(5.66 to 13.93) | 15.59%(109.59% to -35.15%) |
| Somalia | 2.46 (1.11 to 4.08) | 21.73 (9.62 to 35.6) | 12.57% (-32.98% to 73.79%) | 3.17 (1.85 to 5.44) | 48.04 (28.29 to 81.66) | -12.34% (-37.64% to 18.73%) | 10.13 (5.89 to 15.26) | 175.72 (104 to 262.61) | -17.41% (-41.24% to 15.29%) | 0.79(1.24 to 0.48) | 4.1(2.46 to 6.48) | 17.62%(86.91% to -28.03%) |
| United Republic of Tanzania | 14.95 (11.13 to 21.04) | 40.6 (31.03 to 58.43) | 54.84% (15.01% to 134.05%) | 12.69 (9.64 to 16.95) | 53.17 (40.95 to 70.08) | -10.53% (-40.85% to 18.67%) | 54.78 (34.46 to 75.94) | 253.08 (160.02 to 350.78) | 0.57% (-23.1% to 26.76%) | 3.36(4.6 to 2.43) | 6.03(4.12 to 8.42) | 23.72%(86.33% to -19.6%) |
| Uganda | 9.21 (6.81 to 12.63) | 41.76 (31.75 to 55.47) | 113.04% (54.54% to 199.63%) | 7.32 (5.54 to 9.18) | 54.12 (41.54 to 66.83) | 8.7% (-20.66% to 47.28%) | 50.92 (39.56 to 63.41) | 414.88 (326.48 to 514.48) | 18.09% (-12.09% to 57.76%) | 2.26(3.45 to 1.45) | 5.69(3.65 to 8.56) | 62.92%(178.04% to -1.57%) |
| Zambia | 4.63 (3.04 to 6.52) | 46.52 (30.57 to 67.39) | 40.74% (-2.88% to 99.48%) | 4.08 (3.02 to 5.5) | 61.94 (47.07 to 81.17) | -8.83% (-32.42% to 23.38%) | 14.58 (8.63 to 20.11) | 258.43 (152.91 to 355.67) | -10.47% (-32.64% to 23.29%) | 1.18(1.65 to 0.81) | 6.57(4.6 to 9.22) | 34.13%(103.24% to -9.7%) |
| Botswana | 0.7 (0.5 to 0.96) | 41.15 (30.26 to 55.27) | 81.57% (22.46% to 184.41%) | 0.86 (0.61 to 1.14) | 61.08 (44.92 to 79.31) | 4.29% (-26.83% to 44.62%) | 4.18 (2.88 to 5.42) | 346.91 (240.75 to 443.27) | 13.65% (-18.48% to 55.58%) | 0.17(0.25 to 0.12) | 7.53(5.17 to 10.63) | 24.44%(96.69% to -22.24%) |
| Lesotho | 0.53 (0.37 to 0.74) | 35.65 (25.24 to 48.83) | 114.82% (48.53% to 225.69%) | 0.7 (0.48 to 0.95) | 54.85 (38.3 to 72.92) | 38.25% (0.98% to 93.48%) | 4.03 (2.98 to 5.1) | 343.6 (256.93 to 429.53) | 39.33% (6.77% to 91.92%) | 0.13(0.17 to 0.09) | 6.23(4.54 to 8.55) | 36.89%(152.4% to -11.55%) |
| Namibia | 0.7 (0.5 to 0.95) | 43.83 (32.58 to 58.54) | 61.23% (12.98% to 136.94%) | 0.55 (0.42 to 0.7) | 39.78 (31.16 to 50.25) | 8.54% (-19.43% to 46.4%) | 3.81 (2.55 to 4.91) | 293.8 (198.27 to 374.52) | 46.06% (-10.93% to 119.72%) | 0.21(0.34 to 0.12) | 8.55(4.97 to 13.77) | 29.78%(180.48% to -35.57%) |
| South Africa | 16.39 (14.79 to 17.9) | 33.93 (30.57 to 37) | 9.97% (-8.46% to 22.32%) | 19.65 (17.65 to 21.64) | 44.11 (39.68 to 48.44) | -8.39% (-18.71% to 5.61%) | 104.78 (84.45 to 122.93) | 254 (200.48 to 293.43) | 13.88% (-4.58% to 45.75%) | 3.72(4.28 to 3.2) | 6.78(5.89 to 7.72) | -12.19%(2.55% to -25.14%) |
| Eswatini | 0.37 (0.24 to 0.56) | 49.97 (33.13 to 74.1) | 66.5% (16.51% to 146.64%) | 0.33 (0.24 to 0.45) | 56.74 (42.31 to 74.41) | 6.11% (-22.8% to 45.26%) | 1.59 (1.13 to 2.07) | 303.16 (214.62 to 389.61) | 9.37% (-15.63% to 50.45%) | 0.07(0.1 to 0.04) | 6.24(4.15 to 9.62) | 32.12%(104.52% to -15.82%) |
| Zimbabwe | 2.3 (1.73 to 3.01) | 25.23 (18.97 to 32.83) | 41.87% (4.24% to 88.54%) | 12.57 (8.12 to 16.51) | 179.06 (116.71 to 231.87) | 9.64% (-29.26% to 48.72%) | 25.01 (18.6 to 30.86) | 410.21 (307.28 to 500.18) | 14.44% (-19.9% to 55.77%) | 0.71(1.04 to 0.48) | 4.76(3.19 to 6.95) | 53.67%(140.65% to 0.72%) |
| Benin | 3.9 (2.06 to 6.61) | 34.55 (23.07 to 50.82) | 43.52% (-5.89% to 107.34%) | 2.4 (1.8 to 3.1) | 48.61 (36.66 to 61.39) | -36.61% (-64.22% to -10.93%) | 9.37 (5.63 to 12.71) | 226.18 (135.36 to 306.1) | 38.36% (-19.75% to 101.36%) | 0.37(0.8 to 0.2) | 2.35(1.36 to 4.47) | -41.99%(-10.94% to -63.65%) |
| Burkina Faso | 6.35 (3.38 to 10.67) | 29.36 (20.18 to 42.74) | 51.28% (6.09% to 112.29%) | 4.26 (2.75 to 5.84) | 46.32 (30.7 to 62.73) | -37.89% (-72.81% to -8.04%) | 16.64 (10.3 to 22.1) | 217.15 (134.16 to 286.87) | 43.82% (-14.4% to 110.99%) | 0.63(1.59 to 0.32) | 2.17(1.22 to 4.6) | -33.45%(1.22% to -57.11%) |
| Cameroon | 4.23 (2.83 to 6.11) | 21.5 (15.17 to 29.67) | 29.72% (-16.73% to 91.13%) | 7.82 (4.6 to 10.86) | 64.17 (38.42 to 87.33) | -20.6% (-47.97% to 8.84%) | 27.1 (14.53 to 41.03) | 271.76 (148.14 to 406.42) | 55.09% (-16.23% to 135.63%) | 0.88(1.93 to 0.49) | 2.65(1.53 to 5.44) | -37.9%(-3.4% to -62.35%) |
| Cabo Verde | 0.31 (0.24 to 0.38) | 64.31 (51.44 to 78.75) | 349.08% (259.58% to 468.64%) | 0.27 (0.23 to 0.32) | 63.14 (53.23 to 74.49) | 206.75% (152.43% to 272.76%) | 1.73 (1.16 to 2.11) | 439.43 (294.67 to 535.07) | 202.2% (69.26% to 352.77%) | 0.01(0.02 to 0.01) | 2.28(1.43 to 3.79) | 34.16%(100.03% to -13.29%) |
| Chad | 3.34 (1.9 to 5.52) | 22.79 (17.16 to 31.77) | 40% (-0.93% to 96.44%) | 3.27 (2.32 to 4.31) | 58.13 (41.74 to 76.3) | -10.43% (-30.84% to 16.3%) | 12.04 (7.47 to 16.39) | 254.9 (158.91 to 342.94) | 92.43% (12.69% to 187.85%) | 0.43(0.91 to 0.26) | 2.14(1.4 to 3.58) | -35.99%(-3.52% to -58.36%) |
| C?te d'Ivoire | 6.81 (3.92 to 10.3) | 33.77 (22.68 to 46.63) | 28.8% (-13.56% to 83.86%) | 6.35 (4.69 to 8.36) | 58.49 (44.47 to 75.22) | -33.99% (-57.79% to -8.24%) | 21.73 (12.38 to 31.19) | 257.41 (147.24 to 366.49) | 39.33% (-19.37% to 102.08%) | 0.75(1.36 to 0.44) | 2.49(1.53 to 4.17) | -47.5%(-23.79% to -67.16%) |
| Gambia | 0.33 (0.23 to 0.48) | 23.71 (16.65 to 33.49) | 42.79% (-10.49% to 123.29%) | 0.37 (0.26 to 0.49) | 39.45 (28.94 to 52.62) | 19.2% (-19.64% to 74.68%) | 0.71 (0.52 to 0.96) | 82.86 (60.54 to 111.48) | 20.87% (-16.1% to 68.73%) | 0.11(0.18 to 0.06) | 4.82(2.82 to 7.92) | 9.07%(89.62% to -39.26%) |
| Ghana | 7.37 (4.94 to 10.36) | 30.71 (21.48 to 42.17) | 0.87% (-30.53% to 47.05%) | 10.77 (5.66 to 14.5) | 60.17 (32.9 to 79.44) | -20.13% (-56.81% to 17.26%) | 42.63 (33.83 to 55.26) | 304.87 (244.47 to 393.6) | -10.85% (-38% to 35.37%) | 0.95(1.9 to 0.55) | 2.69(1.6 to 5.28) | -33.43%(-1.4% to -58.35%) |
| Guinea | 2.91 (1.75 to 4.69) | 26.41 (18.17 to 38.27) | 23.8% (-21.5% to 88.13%) | 6.54 (4.34 to 9.18) | 122.03 (81.75 to 169.48) | 9.76% (-28.17% to 63.64%) | 14.47 (7.61 to 22.3) | 298.42 (157.09 to 456.56) | 24.55% (-10.49% to 75.47%) | 0.8(1.42 to 0.47) | 6.41(3.85 to 10.43) | 9.77%(76.81% to -35.62%) |
| Guinea-Bissau | 0.38 (0.25 to 0.56) | 29.64 (21.43 to 40.47) | 4.2% (-32.46% to 69.81%) | 0.49 (0.36 to 0.66) | 65.6 (50.02 to 85.72) | -29.98% (-50.36% to -3.64%) | 1.5 (0.81 to 2.29) | 247.59 (136.6 to 368.77) | 34.36% (-19.9% to 95.82%) | 0.05(0.1 to 0.03) | 2.32(1.47 to 4.25) | -48%(-16.01% to -65.12%) |
| Liberia | 0.88 (0.49 to 1.38) | 27.15 (15 to 40.86) | -11.8% (-50.39% to 58.15%) | 1.09 (0.75 to 1.63) | 52.1 (37.51 to 77.1) | -42.24% (-64.43% to -10.92%) | 3.9 (2.38 to 5.55) | 237.8 (144.34 to 332.49) | 35.5% (-20.28% to 106.15%) | 0.09(0.15 to 0.06) | 1.81(1.12 to 2.66) | -57.56%(-29.36% to -76.79%) |
| Mali | 5.98 (3.18 to 10.21) | 28.15 (18.81 to 41.8) | 38.4% (-10.36% to 100.74%) | 16.4 (7.23 to 22.69) | 197.04 (86.49 to 270.59) | -2.66% (-57.82% to 40.37%) | 8.51 (6.51 to 11.13) | 116.02 (89.01 to 150) | 28.74% (2.42% to 65.62%) | 0.81(1.98 to 0.41) | 3.26(1.79 to 6.59) | 11.55%(85.64% to -32.47%) |
| Mauritania | 0.74 (0.47 to 1.08) | 26.83 (17.8 to 37.35) | 3.59% (-36.42% to 64.04%) | 0.96 (0.66 to 1.41) | 46.99 (32.53 to 67.15) | -43.8% (-60.54% to -20.52%) | 4.31 (2.69 to 5.7) | 236.95 (148.55 to 309.5) | 49.01% (-10.99% to 131.15%) | 0.08(0.13 to 0.04) | 1.83(1.05 to 2.94) | -58.08%(-29.33% to -76.61%) |
| Niger | 3.33 (1.84 to 5.51) | 16.95 (11.91 to 23.66) | -10.28% (-46.61% to 49%) | 3.39 (2.09 to 4.85) | 44.55 (28.17 to 63.41) | -34.47% (-57.94% to -9.72%) | 13.45 (8.37 to 19.37) | 214.24 (133.77 to 302.86) | 54.95% (-4.17% to 128%) | 0.56(1.27 to 0.31) | 1.89(1.17 to 3.28) | -47.71%(-17.55% to -66.46%) |
| Nigeria | 42.14 (26 to 63.99) | 25.31 (17.9 to 34.86) | 18% (-21.19% to 81.12%) | 16.93 (13.19 to 22.06) | 21.35 (16.92 to 27.26) | 9.3% (-25.35% to 65.94%) | 272.44 (123.95 to 417.16) | 395.36 (182.6 to 597.3) | 33.17% (-6.83% to 93.32%) | 2.97(8.17 to 1.44) | 1.03(0.54 to 2.62) | -21.57%(37.78% to -48.6%) |
| Sao Tome and Principe | 0.06 (0.03 to 0.1) | 34.73 (20.96 to 54.51) | 5.74% (-41.21% to 91.2%) | 0.11 (0.08 to 0.14) | 100.48 (77.01 to 132.92) | 33.7% (-0.63% to 81.12%) | 0.16 (0.12 to 0.21) | 178.77 (134.54 to 236.32) | 50.03% (13.88% to 104.92%) | 0.01(0.01 to 0) | 2.33(1.62 to 3.24) | -32.42%(21.31% to -61.08%) |
| Senegal | 3.06 (2.14 to 4.15) | 27.72 (20.42 to 35.9) | 9.14% (-28.45% to 72.09%) | 4.2 (2.64 to 5.65) | 56.33 (36.02 to 74.63) | -29.55% (-51.52% to -2.44%) | 17.7 (11.09 to 23.78) | 270.61 (168.14 to 361.85) | 49.27% (-10.77% to 125.18%) | 0.41(0.57 to 0.26) | 2.4(1.58 to 3.32) | -46.16%(-11.53% to -68.82%) |
| Sierra Leone | 2.15 (1.23 to 3.54) | 29.42 (19.69 to 42.94) | 16.86% (-29.26% to 97.96%) | 1.77 (1.13 to 2.44) | 48.61 (31.22 to 66.1) | -40.8% (-78.58% to -1.35%) | 7.14 (4.44 to 10.02) | 230.66 (142.84 to 321.03) | 42.35% (-16.1% to 117.13%) | 0.24(0.53 to 0.13) | 2.38(1.36 to 4.76) | -38.03%(-1.78% to -60.19%) |
| Togo | 1.52 (1.05 to 2.4) | 26.19 (18.75 to 37.52) | 22.81% (-14.11% to 68.21%) | 1.92 (1.11 to 2.64) | 51.19 (30.62 to 69.09) | -37.34% (-69.62% to -8.59%) | 6.48 (3.62 to 9.33) | 211.53 (119.89 to 298.19) | 33.96% (-25.71% to 108.67%) | 0.19(0.39 to 0.11) | 2.17(1.31 to 4.11) | -48.82%(-25.29% to -66.31%) |
| American Samoa | 0.01 (0.01 to 0.02) | 23.34 (18.51 to 29.35) | 5.94% (-24.32% to 40.47%) | 0.02 (0.02 to 0.03) | 48.88 (41.08 to 57.67) | 31.85% (5.75% to 62.39%) | 0.14 (0.12 to 0.17) | 338.55 (274.18 to 398.18) | -2.58% (-19.41% to 22.75%) | 0(0 to 0) | 0.92(0.67 to 1.28) | 114.15%(211.99% to 48.85%) |
| Bermuda | 0.07 (0.06 to 0.09) | 66.46 (53.74 to 82.5) | -46.26% (-57.36% to -32.18%) | 0.11 (0.09 to 0.13) | 84.61 (70.01 to 102.1) | -32.37% (-44.97% to -16.12%) | 0.46 (0.36 to 0.6) | 336.07 (260.67 to 437.39) | -0.6% (-23.44% to 30.18%) | 0(0 to 0) | 1.96(1.47 to 2.63) | 151.64%(256.21% to 63.89%) |
| Cook Islands | 0 (0 to 0.01) | 19.56 (15.37 to 24.45) | 11.11% (-21.89% to 55.95%) | 0.01 (0.01 to 0.02) | 61.51 (50.32 to 74.35) | -11.39% (-33.4% to 13.88%) | 0.11 (0.09 to 0.13) | 425.32 (350.55 to 515.47) | -23.67% (-36.52% to -8.57%) | 0(0 to 0) | 2.82(1.98 to 3.92) | -3.21%(45.13% to -38.95%) |
| Greenland | 0.11 (0.08 to 0.13) | 148.46 (117.09 to 182.15) | 47.77% (13.64% to 85.49%) | 0.06 (0.05 to 0.07) | 85.35 (69.5 to 105.37) | -26.67% (-41.71% to -8.93%) | 0.08 (0.06 to 0.1) | 121.2 (94.87 to 152.58) | 14.43% (-15.1% to 53.4%) | 0.01(0.01 to 0) | 12.13(7.65 to 19.12) | -55.74%(-17.59% to -76.64%) |
| Guam | 0.07 (0.06 to 0.09) | 38.57 (30.88 to 47.68) | -19.15% (-39.31% to 5.87%) | 0.07 (0.06 to 0.09) | 37.99 (31.13 to 46.23) | 5.29% (-16.37% to 32.5%) | 0.23 (0.19 to 0.3) | 126.48 (100.51 to 160.55) | -21.3% (-36.99% to -0.64%) | 0(0 to 0) | 1.64(1.21 to 2.18) | 138.96%(245.91% to 60.72%) |
| Monaco | 0.08 (0.06 to 0.1) | 98.25 (77.08 to 122.58) | 15.92% (-14.8% to 54.48%) | 0.17 (0.12 to 0.3) | 174.33 (126.67 to 311.57) | 18.87% (-16.61% to 99.05%) | 0.18 (0.14 to 0.25) | 173.55 (137.37 to 240.64) | 1.8% (-19.2% to 31.34%) | 0.01(0.02 to 0.01) | 33.17(21.21 to 49.59) | -22.39%(30.09% to -53.19%) |
| Nauru | 0 (0 to 0) | 41.23 (30.43 to 54.89) | 2.5% (-36.71% to 42.47%) | 0 (0 to 0) | 62.33 (47.47 to 79.7) | 8.36% (-14.18% to 38.23%) | 0.01 (0 to 0.01) | 259.72 (177.8 to 364.38) | 8.28% (-25.23% to 52.9%) | 0(0 to 0) | 4.03(2.63 to 6.73) | 3.07%(43.9% to -25.94%) |
| Niue | 0 (0 to 0) | 37.35 (27.47 to 50.75) | 29.51% (-11.25% to 82.56%) | 0 (0 to 0) | 45.49 (35.51 to 56.31) | 8.04% (-17.22% to 37.73%) | 0 (0 to 0.01) | 213.06 (160.27 to 258.6) | 21.82% (-8.89% to 61.21%) | 0(0 to 0) | 3.71(2.49 to 5.55) | 5.97%(71.69% to -31.68%) |
| Northern Mariana Islands | 0.02 (0.02 to 0.03) | 43.18 (33.74 to 53.3) | -3.75% (-31.6% to 32.6%) | 0.03 (0.02 to 0.03) | 54.52 (45.2 to 65.03) | 85.7% (45.91% to 128.18%) | 0.09 (0.07 to 0.11) | 212.92 (172.73 to 251.59) | 15.83% (-13.07% to 50.52%) | 0(0 to 0) | 1.02(0.76 to 1.42) | 102.65%(194.21% to 43.87%) |
| Palau | 0 (0 to 0.01) | 20.59 (15.85 to 26.43) | 29.44% (-11.31% to 82.6%) | 0 (0 to 0.01) | 21.85 (17.56 to 27.45) | 0.95% (-26.36% to 34.2%) | 0.05 (0.04 to 0.06) | 260.86 (206.43 to 341.84) | -4.51% (-25.9% to 23.07%) | 0(0 to 0) | 0.3(0.24 to 0.37) | 6.28%(38.61% to -18.58%) |
| Puerto Rico | 2.8 (2.13 to 3.68) | 48.42 (36.66 to 63.92) | -13.59% (-34.65% to 13.18%) | 3.08 (2.39 to 3.96) | 42.76 (33.04 to 55.2) | -6.89% (-29.34% to 21.08%) | 13.67 (10.15 to 20.56) | 176.47 (130.4 to 266.53) | -24.85% (-44.27% to 7.36%) | 0.31(0.41 to 0.22) | 9.29(6.52 to 12.53) | 199.68%(328.81% to 84.47%) |
| Saint Kitts and Nevis | 0.05 (0.04 to 0.07) | 77.1 (58.73 to 96) | -44.46% (-58.39% to -29.23%) | 0.04 (0.04 to 0.05) | 67.51 (56.94 to 79.65) | -13.67% (-29.72% to 5.18%) | 0.42 (0.34 to 0.52) | 714.91 (570.18 to 877.44) | 32.69% (10.47% to 59.37%) | 0.01(0.01 to 0) | 11.02(5.98 to 15.74) | 275.21%(488.69% to 92.4%) |
| San Marino | 0.03 (0.02 to 0.04) | 52.26 (33.75 to 76.84) | 13.26% (-30.3% to 74%) | 0.09 (0.06 to 0.13) | 141.85 (94.66 to 202.9) | -6.15% (-38.89% to 38.1%) | 0.13 (0.09 to 0.2) | 187.99 (122.66 to 292.69) | -0.16% (-36.7% to 50.35%) | 0(0 to 0) | 5.23(3.37 to 8.31) | -12.14%(45.71% to -45.98%) |
| Tokelau | 0 (0 to 0) | 23 (16.2 to 32.58) | 27.9% (-11.38% to 77.22%) | 0 (0 to 0) | 40.7 (31.31 to 54.43) | 4.64% (-22.6% to 41.38%) | 0 (0 to 0) | 174.94 (136 to 219.22) | 12.94% (-13.97% to 53.37%) | 0(0 to 0) | 2.77(1.79 to 3.99) | 7.43%(69.04% to -34.77%) |
| Tuvalu | 0 (0 to 0) | 24.64 (16.11 to 36.1) | 11.29% (-21.47% to 61.2%) | 0 (0 to 0.01) | 46.89 (36.35 to 61.2) | 2.18% (-24.02% to 38.43%) | 0.02 (0.01 to 0.02) | 206.07 (158.2 to 269.71) | 14.98% (-15.6% to 61.14%) | 0(0 to 0) | 3.14(2.1 to 4.46) | 13.33%(72.7% to -23.85%) |
| United States Virgin Islands | 0.17 (0.14 to 0.21) | 102.85 (80.5 to 128.12) | 23.01% (-10.93% to 68.33%) | 0.08 (0.07 to 0.1) | 41.96 (34.88 to 50.72) | 30.06% (-1.71% to 75.35%) | 1.22 (1 to 1.53) | 625.68 (513.05 to 780.49) | 28.23% (-1.38% to 73.97%) | 0(0 to 0) | 0.92(0.6 to 1.39) | 38.42%(130.81% to -16.32%) |
| South Sudan | 2.03 (1.35 to 3.81) | 33.42 (21.79 to 61.9) | 10.09% (-26.08% to 68.05%) | 1.88 (1.2 to 2.92) | 51.65 (33.98 to 78.24) | -11.02% (-35.41% to 23.53%) | 7.35 (4.6 to 10.57) | 232.28 (146.41 to 329.48) | -3.06% (-29.13% to 32.93%) | 0.38(0.55 to 0.26) | 4.37(2.99 to 6.43) | 4.37%(64.73% to -30.33%) |
| Sudan | 8.99 (5.05 to 14.96) | 33.39 (18.16 to 56.35) | 83.17% (11.5% to 161.1%) | 10.81 (7.07 to 19.11) | 61.03 (40.2 to 108.07) | -14.9% (-47.21% to 56.29%) | 15.39 (10.62 to 20.88) | 98.93 (68.39 to 133.42) | 27.97% (-2.37% to 73.73%) | 1.24(1.8 to 0.78) | 2.91(1.88 to 4.21) | 76.37%(219.52% to -7.75%) |
